# Synthesis of 2,2-difluoro-1,3-diketone and 2,2-difluoro-1,3-ketoester derivatives using fluorine gas

**DOI:** 10.3762/bjoc.20.41

**Published:** 2024-02-28

**Authors:** Alexander S Hampton, David R W Hodgson, Graham McDougald, Linhua Wang, Graham Sandford

**Affiliations:** 1 Durham University, Department of Chemistry, Lower Mountjoy, South Road, Durham, DH1 3LE, UK; 2 Syngenta, Huddersfield Manufacturing Centre, PO Box A38, Huddersfield, West Yorkshire, HD2 1FF, UK; 3 Syngenta USA, 410 Swing Road, Greensboro, North Carolina, NC 27409, USA

**Keywords:** difluorination, difluoromethylene, direct fluorination, electrophilic fluorination, organofluorine

## Abstract

Solutions of 1,3-diketones and 1,3-ketoester derivatives react with fluorine to give the corresponding 2,2-difluoro-1,3-dicarbonyl derivatives in the presence of quinuclidine. Quinuclidine reacts with fluorine in situ to generate a fluoride ion that facilitates limiting enolization processes, and an electrophilic N–F fluorinating agent that is reactive towards neutral enol species.

## Introduction

Fluorine is present in many agrochemical and pharmaceutical products owing to the beneficial properties imparted such as increased metabolic stability, lipophilicity and bioavailability of the bioactive entity [1–3]. In 2018, 30% of FDA approved drugs contained at least one fluorine atom, with an average of 2.7 fluorine atoms per fluorinated drug, and fluorine is also present in the structures of 50% of marketed agrochemicals [4]. In the context of the research reported here, the incorporation of difluoromethylene (CF_2_) units into life science products is growing in importance and a number of commercially significant pharmaceuticals [lubiprostone (constipation), maraviroc (HIV), tafluproct (anti-inflamatory), ledipasvir (hepatitis-C)] and agrochemicals [isopyrazam (fungicide), riodipine (calcium channel blocker), primisulfuron-methyl (pesticide)] owe their enhanced bioactivity, in part, to the presence of difluoromethylene units.

To meet the demands of synthetic chemists within the life science discovery and manufacturing arenas, many fluorination methods have been developed over the years to introduce difluoromethylene groups into organic systems. Approaches using nucleophilic fluorination include halogen exchange of *gem*-dihalo groups to corresponding CF_2_ derivatives using silver tetrafluoroborate [5] or mercury(II) fluoride [6], deoxyfluorination of carbonyl derivatives using diethylaminosulfur trifluoride (DAST) or related Deoxo-Fluor and Xtalfluor reagents [7–8]. Alternatively, oxidative fluorodesulfurizations of carbonyl derivatives using a combination of sources of halonium and fluoride ions such as 1,3-dibromo-5,5-dimethylhydantoin (DBH) and tetrabutylammonium dihydrogen trifluoride have been achieved [9–11].

The transformation of methylene to difluoromethylene using electrophilic fluorinating agents offers an alternative fluorination route, for example, the reactions of MeCN solutions of 1,3-diketones with electrophilic fluorinating agents such as Selectfluor eventually give the corresponding 2,2-difluoro-1,3-diketone derivatives [12]. Monofluorination of the 1,3-diketone substrates is rapid, but the second fluorination step occurs only after reaction for several days. In the solid phase, mechanical milling of the diketone substrate with solid Selectfluor in the presence of sodium carbonate [13–14], and reaction of ketones with a strong base and an N–F reagent give rise to the corresponding 2,2-difluoroketones [15]. In related kinetic studies concerning the electrophilic 2-fluorination of 1,3-diketones with Selectfluor [16–17], we demonstrated that the rate-determining step for difluorination was enolization of the intermediate 2-fluoro-1,3-diketone. Monofluorination of 1,3-diketones occurs rapidly because the substrates lie predominantly in their enol tautomeric forms. The resulting 2-difluoro-1,3-diketones, on the other hand, are formed in their keto-tautomeric forms. Thus, we found difluorination could only be achieved upon addition of water or a base to accelerate the enolization of the monofluoro-diketone intermediates. In addition, imines and α-diboryl ketone derivatives can also be transformed to 2,2-difluoroketones using an N–F electrophilic fluorinating reagent [18]. Alternatively, building blocks containing CF_2_ units such as ethyl bromodifluoroacetate and difluoromethylphenyl sulfoxide offer the possibility of transferring difluoromethylene groups directly into organic systems [19–25] and there is now a very extensive literature on carbon–carbon bond-forming reactions using these and other difluoromethylated building blocks [3,26–32].

Since profit margins in the life science industries are always under constant pressure, less expensive methods of introducing fluorine selectively into active intermediates for manufacture on the industrial scale are required and, as a relatively inexpensive strategy, direct fluorination of substrates using fluorine gas has been used successfully for the production of 5-fluorouracil (generic, anticancer) and voriconazole (V-FEND, Pfizer, antifungal) [33]. Methods have been developed for the selective monofluorination of 1,3-dicarbonyl derivatives by fluorine gas using batch and continuous flow techniques [34–36]. Difluorination occurs very slowly in comparison to monofluorination, although some difluorinated by-products are, in general, formed upon fluorination of dicarbonyl substrates and difluorinated products can be readily separated from monofluorinated systems [34]. Direct fluorination of diazo compounds using F_2_ [37] is the only report of a useful synthetic procedure to selectively prepare a difluoromethylene containing product using F_2_ but, in these cases, CFCs, now banned under the Montreal protocol, were used as the reaction medium.

Here, we demonstrate that the addition of quinuclidine to direct fluorination reactions of 1,3-diketone and 1,3-ketoester substrates using fluorine gas can give difluorinated products by a simple batch process, offering a potentially valuable route to the synthesis of difluoromethylene compounds that is suitable for inexpensive scale-up.

## Results

**Scheme 1:**
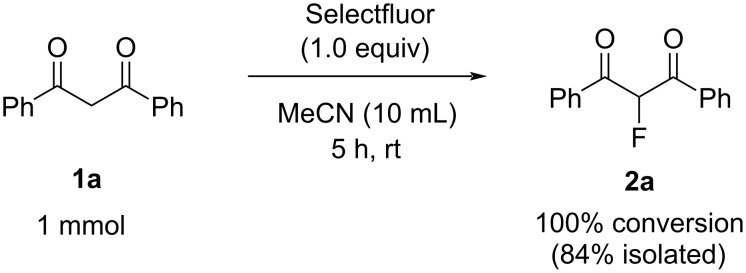
Monofluorination of 1,3-diphenylpropane-1,3-dione with Selectfluor.

2-Fluorinations of 1,3-diaryldiketone derivatives such as 1,3-diphenylpropane-1,3-dione (dibenzoylmethane, DBM, **1a**) using electrophilic fluorinating reagents such as Selectfluor, NFSI, and NFOBS under a range of conditions have been described extensively [3,12–13,30,38–43]. We confirmed that reaction of compound **1a** with Selectfluor in acetonitrile (MeCN) gave high yields of the monofluorinated product **2a** with no difluorinated product being observed by ^19^F NMR analysis of the product mixture after 5 h (Scheme 1).

In contrast, attempts to fluorinate **1a** with one equivalent of fluorine gas in MeCN gave no noticeable conversion on analysis by ^19^F NMR spectroscopy, and a large excess of fluorine led to formation of a dark brown tar from which no useful product could be isolated. On the bases of these failed attempts, coupled with our previous experiences with the DBM scaffold [16–17,36], we used the difluorination of **1a** with fluorine gas as a model process to assess how direct fluorination reactions could be achieved using reaction additives.

The lack of reactivity of **1a** towards one equiv of fluorine gas when compared with strong reactivity towards Selectfluor suggested the use of a cationic, electrophilic reagent to be important. Given the structural similarity of 1,4-diazabicyclo[2.2.2]octane (DABCO) to the Selectfluor system, a 10% v/v mixture of fluorine in nitrogen was passed through a solution of **1a** in acetonitrile containing one equivalent of DABCO, using a fluorination apparatus and gas flow controller equipment described previously [35]. Our aim was to form a N–F system in situ and thus mimic the successful monofluorination observed between **1a**-enol and Selectfluor. After purging the product mixture with nitrogen, a known quantity of α,α,α-trifluorotoluene was added to the product mixture and the crude yields of fluorinated products were estimated by ^19^F NMR integration (monofluoro product **2a**, δ_F_ −189.9 ppm; difluoro product **3a**, δ_F_ −102.7 ppm) (Table 1, entry 3).

**Table 1:** Screening conditions for the fluorination of 1,3-diphenylpropane-1,3-dione (**1a**).**^a^**

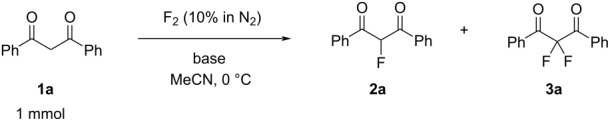						

Entry	Base additive	Equiv of additive	Equiv of F_2_	Crude yield by ^19^F NMR spectroscopy^a,b^		
				**1a** [%]	**2a** [%]	**3a** [%]

1	–	–	1	100	0	0
2	–	–	20	polyfluorinated tar		
3	DABCO	1	1	32	4	20
4	DABCO	1	2	1	1	37
5	DABCO	1	3	polyfluorinated tar		
6	DABCO	2	2	many fluorinated products		
7	DABCO	0.1	1	22	28	8
8	quinuclidine	1	1	42	10	43
9	quinuclidine	1.2	1	54	1	43
10	Et_3_N	1	1	56	25	6
11	Cs_2_CO_3_	1	1	0	4	14
12	NaCl	1	1	0	33	12

^a^Conversion levels determined by NMR spectroscopy by comparing the integrals (CF dp at −189.9 ppm, CF_2_ s at −102.7 ppm) to α,α,α-trifluorotoluene standard. ^b^The mass balances included mixtures of soluble, unidentified products, and insoluble materials.

Using excess fluorine or DABCO (entries 5 and 6 in Table 1) led to the formation of tars, while 0.1 equiv of DABCO (entry 7) gave only relatively low conversions to **2a** and **3a**. Other organic nitrogen bases were tested, and we found that quinuclidine (entries 8 and 9, Table 1) gave high conversion to difluorinated product **3a**, with very little monofluorinated product **2a** being observed. Suspensions of caesium carbonate or sodium chloride (entries 11 and 12 in Table 1) also gave some **2a** and **3a**, but also unwanted tar.

**Scheme 2:**
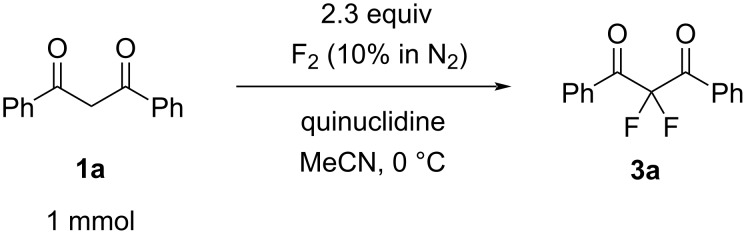
Synthesis of 2,2-difluoro-1,3-diphenylpropane-1,3-dione (**3a**).

**Figure 1:**
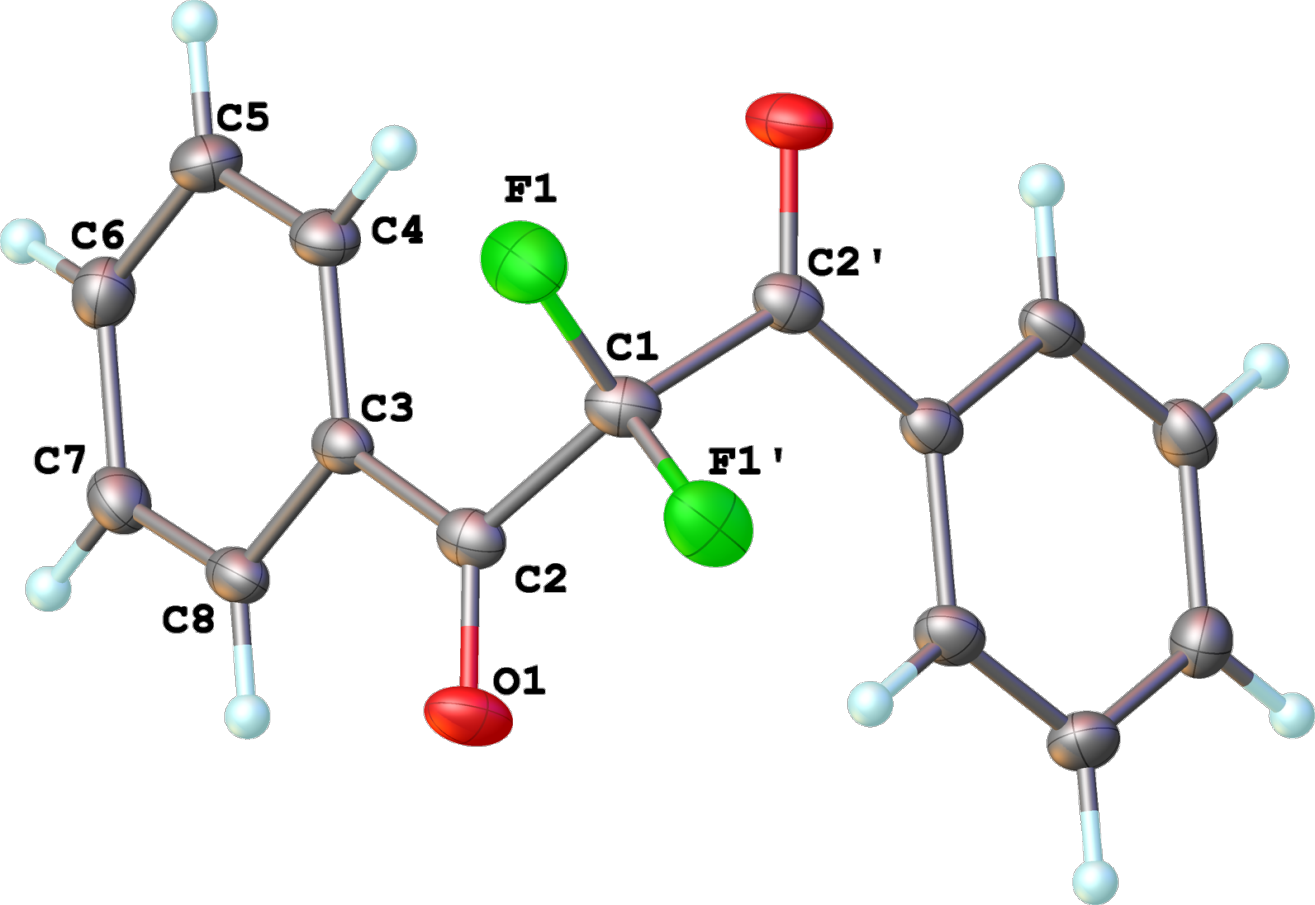
Molecular structure of 2,2-difluoro-1,3-diphenylpropane-1,3-dione (**3a**).

This set of reactions showed that the basic species we screened all facilitated mono- and difluorination to some degree. The quinuclidine-mediated fluorination of **1a** led to the highest conversion to difluorinated product **3a** so we next sought to optimize this process at a preparative scale by varying the reaction stoichiometry. We found that 2.3 equiv of fluorine and 1.1 equiv of quinuclidine gave 99% conversion of **1a** with **2a** and **3a** being the only products observed by ^19^F NMR spectroscopy in a 16:120 ratio (see Supporting Information File 1). To isolate the main difluorinated product **3a**, the reaction vessel was purged with nitrogen and the product mixture was partitioned between water and DCM to remove HF and salt by-products. Purification of **3a** by column chromatography gave **3a** as a white crystalline solid in 65% isolated yield (Scheme 2) and the structure was confirmed by NMR spectroscopy and X-ray crystallography (Figure 1).

To expand the substrate scope of this difluorination method, a range of DBM derivatives **1b**–**n** was synthesized from *para-*substituted acetophenones, *para-*substituted benzoyl chlorides and lithium hexamethyldisilazane following a literature procedure reported by Liu and co-workers (see Supporting Information File 1) [44]*.* Subsequently, difluorinations of DBM substrates **1b**–**n** were performed under conditions similar to those optimized for the preparation of **3a**. The desired difluorinated products **3b**–**n** were synthesized and isolated in good yields (Table 2).

**Table 2:** Difluorination of dibenzoylmethane derivatives **3a**–**n** using fluorine gas and quinuclidine.

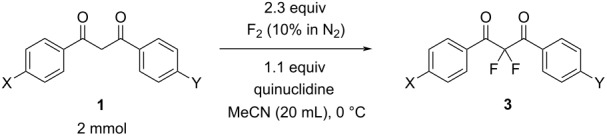				

Entry	1,3-Diketone	Product	Structure	Isolated yield [%]

1	**1a**	**3a**	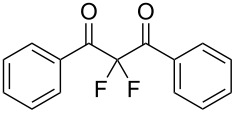	65
2	**1b**	**3b**	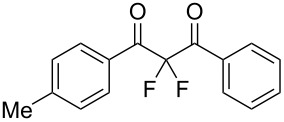	41^a^ 10^a^ (**7a**) 12^a^ (Ar–F)
3	**1c**	**3c**	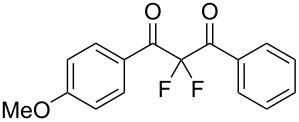	31^a^ 16^a^ (Ar–F)
4	**1d**	**3d**	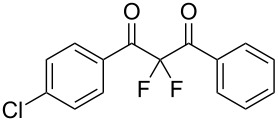	60
5	**1e**	**3e**	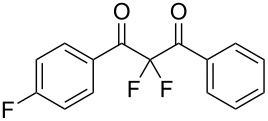	59
6	**1f**	**3f**	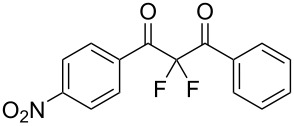	50
7	**1g**	**3g**	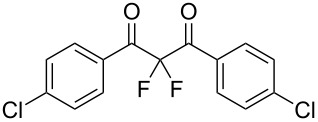	72
8	**1h**	**3h**	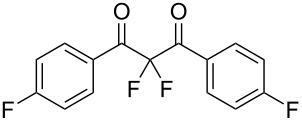	76
9	**1i**	**3i**	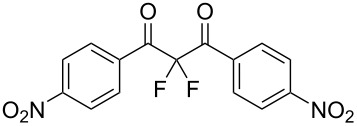	77

^a^Conversion estimated by NMR spectroscopy.

Unfortunately, substrates bearing electron-donating groups **1b** (–Me) and **1c** (–OMe) reacted with fluorine to give tarry materials and products arising from fluorination of both the desired enolic sites and the aryl rings. No products could be isolated from these complex mixtures and yields were estimated by ^19^F NMR spectroscopy.

**Figure 2:**
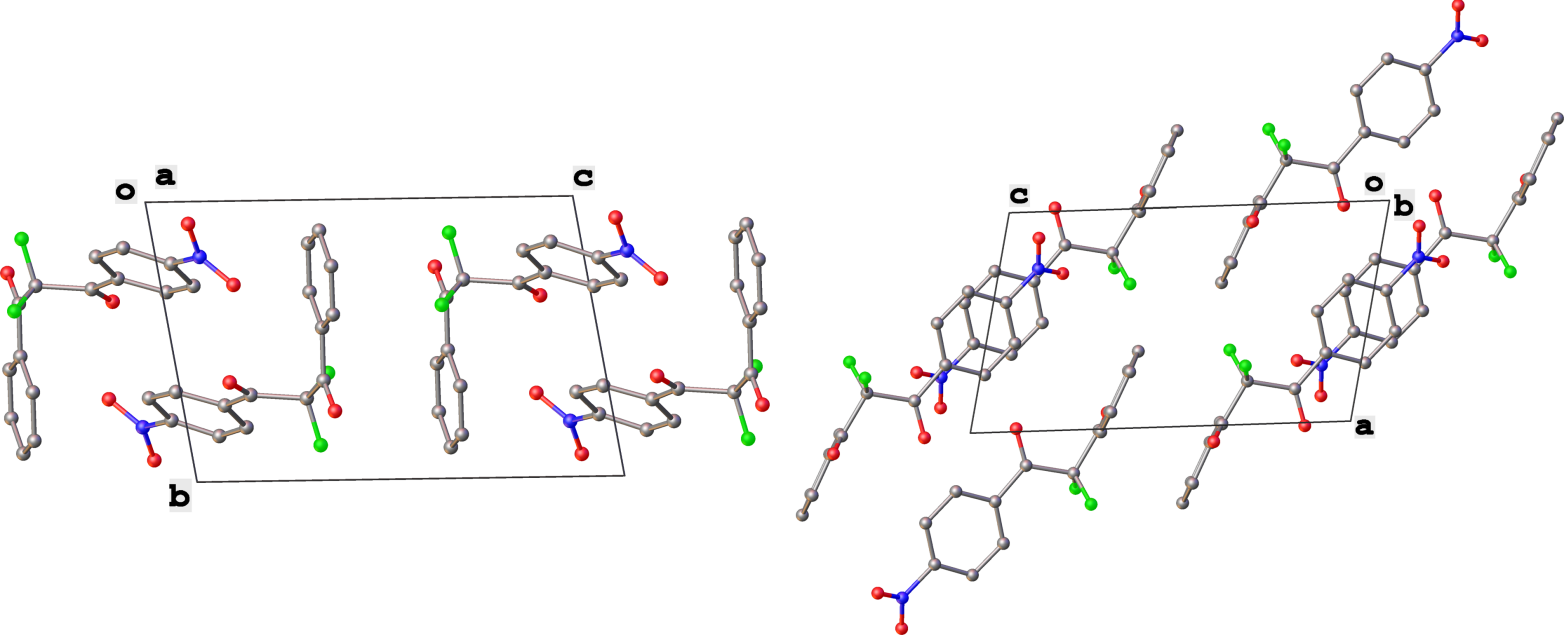
Crystal packing structure of **3f** as determined by SXRC.

In contrast, substrates bearing electron-withdrawing groups deactivated the aryl rings sufficiently to suppress competing ring fluorination and difluorinated products **3d**–**i** could be isolated in high yields. Again, purification by column chromatography gave the products **3** as white crystalline solids and the structures of compounds **3f** and **3i** were confirmed by X-ray crystallography (Figure 2 and Supporting Information File 1). Molecules **3a, f**, and **i** all exist in the solid state with the dicarbonyl moiety rotated to maximize the distances between the lone pairs of the electron-rich fluorine and oxygen atoms. Usually, one of the fluorine atoms lies in a *syn* orientation to an oxygen (e.g., **3f** has an F–C–C–O dihedral angle of 15.6°) creating a dipole. This dipole appears to aid crystal packing by forming weak intermolecular interactions with an aryl ring in an adjacent molecule. The two aryl rings within the molecule are near-perpendicular to each other and this conformation leads to enhanced, orthogonal π-stacking interactions.

We next turned our attention to difluorination of related 2-ketoester substrates. Monofluorination of 2-ketoesters using fluorine gas has been scaled up to the manufacturing level [33], whereas preparative methods for the synthesis of 2,2-difluoro-3-ketoesters using fluorine gas have not been realized. Ethyl benzoylacetate (**4a**) was used as a model system for the development of conditions for selective difluorination using fluorine gas. After screening basic additives as mediating agents and subsequent optimization (see Supporting Information File 1), we found that reaction of ethyl benzoylacetate (**4a**), quinuclidine (1.5 equiv), and fluorine (3 equiv) in acetonitrile gave the desired difluorinated product **5a** in 85% isolated yield. Purification of **5a** was achieved very readily by eluting the reaction mixture through a small quantity of silica gel with chloroform and evaporating the residual solvent to leave the crude product which could be further purified by recrystallization. Subsequently, a range of ethyl benzoylacetate derivatives was prepared (see Supporting Information File 1) [45–46] and successfully subjected to difluorination conditions (Table 3).

**Table 3:** Quinuclidine-mediated direct fluorination of ethyl benzoylacetate derivatives **4a**–**g**.

Entry	Product	Structure	Yield/%

1	**5a**	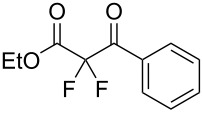	85
2	**5b**	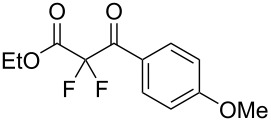	not isolated
3	**5c**	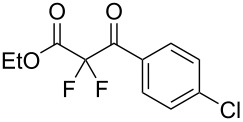	89
4	**5d**	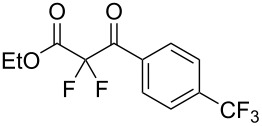	87
5	**5e**	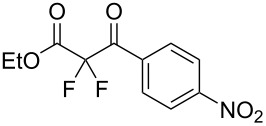	83
6	**5f**	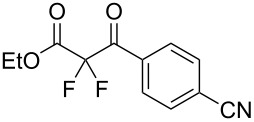	67
7	**5g**	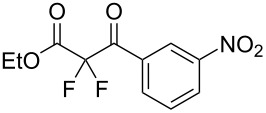	84
8	**5h**	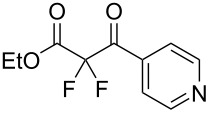	not isolated

**Figure 3:**
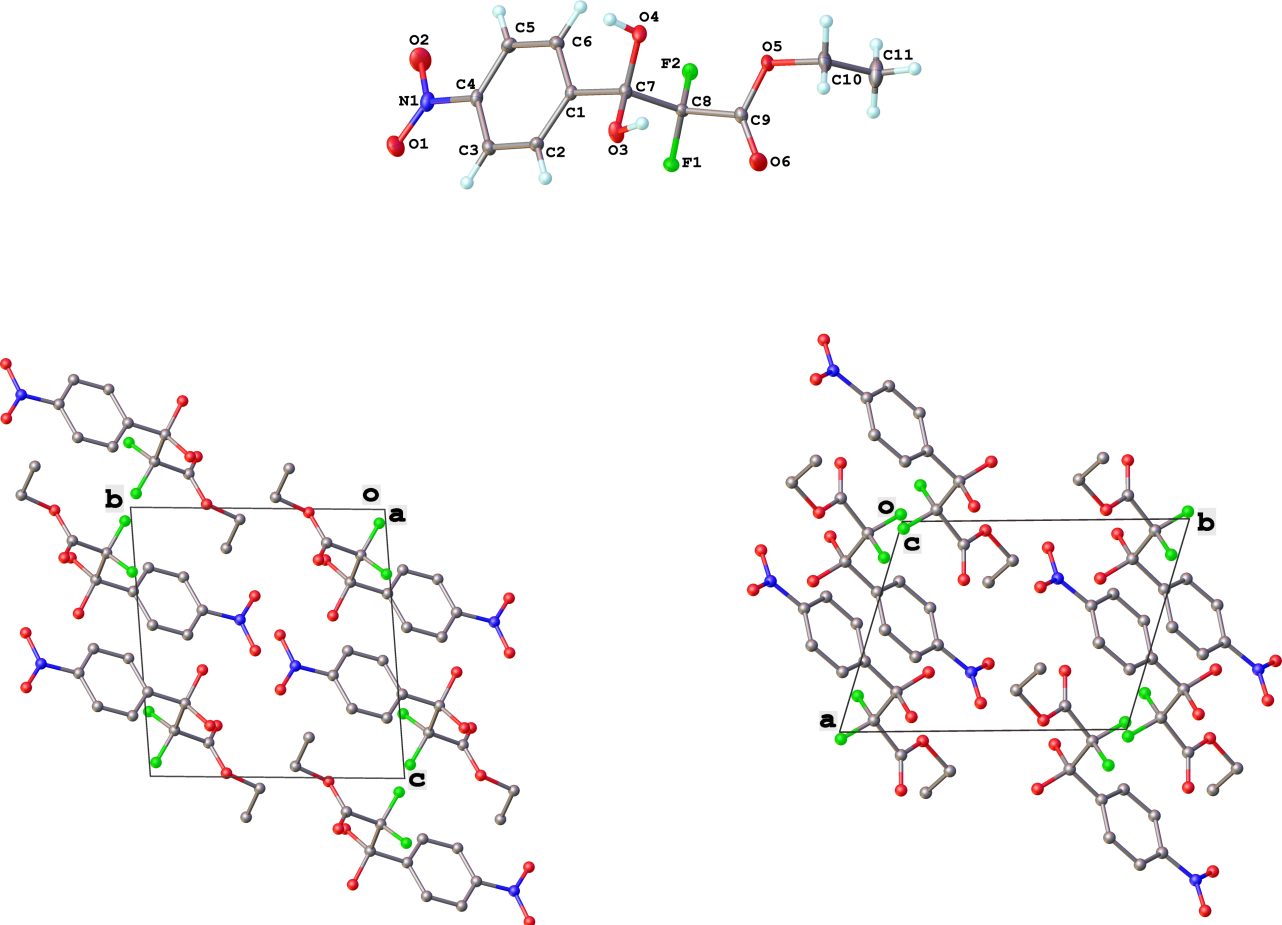
Molecular structure and crystal packing of **5e** as determined by SXRC.

Purification by column chromatography using the minimum amount of silica gel with chloroform as the eluent yielded **5c**–**g** in high yields. As was observed in attempted fluorination reaction of **1c** towards difluorodiketone **3c**, methoxy ketoester derivative **4b** gave substantial amounts of product arising from competing fluorination of the aromatic ring. Structures of difluorinated ketoesters **5a**–**h** were confirmed by NMR spectroscopy. The ^13^C{^1^H} NMR spectra contained signals supporting the presence of ketone (e.g., δ_C_ = 185.6 ppm for **5a**) and ester (δ_C_ = 161.9 ppm for **5a**) functionalities. Difluoroketoester products were found to hydrate readily to give *gem*-diol derivatives during aqueous work-up [39], thus reducing the efficiency of extraction. Indeed, attempts to grow a single crystal of **5e** from a mixture of EtOH and water led to the isolation of the corresponding *gem*-diol (Figure 3). There are very few examples of organic structures containing a C(OH)_2_–CF_2_–C fragment in the CCDC and only three acyclic examples (CSD 5.43 (Nov. 2021); ref codes IZICEA [47], XOPZEK and XOPZIO [48]) are known. Interestingly, in contrast to the previously described acyclic structures no OH···O(H) hydrogen bonds are present in structure **5e** – the molecules are linked by OH···O(NO_2_) interactions.

## Discussion

**Scheme 3:**
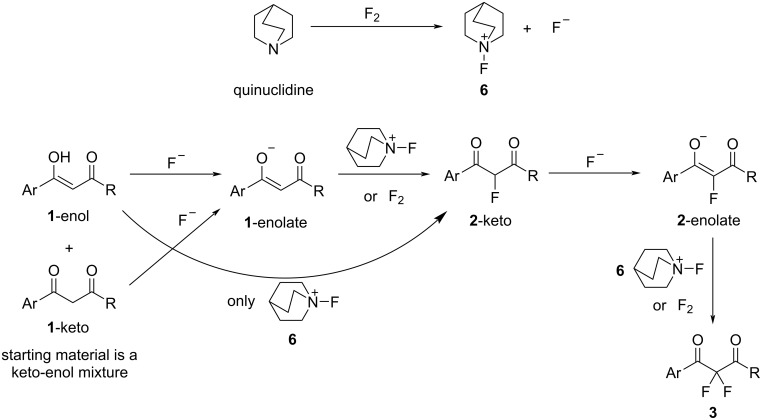
Proposed mechanism of the quinuclidine-mediated difluorination of 1,3-dicarbonyl substrates.

Keto–enol tautomer studies have shown that DBM **1a** and related systems **1b**–**i** exist almost entirely (ca. 90%) in their enolic forms in MeCN [17]. Our initial experiments showed **1a** to be unreactive towards 1 equiv of fluorine gas, suggesting that the neutral enol group and neutral, elemental fluorine do not react to give the desired 2-fluoro-1,3-diketone **2a**. Supplementation of the reaction mixture with either a tertiary amine or inorganic base led to varying mixtures of mono- and difluoro products **2a** and **3a**, respectively, with the tertiary amines proving most effective. Inorganic bases offer the possibility of deprotonating **1a**-enol to form a more reactive enolate **1a**-enolate. Nitrogen-centered bases react with fluorine gas to form *N*-fluoroammonium fluorides and fluoride ion [49]. Thus, on addition of tertiary amines, fluorine can react to generate basic fluoride ions and deliver reactive, electrophilic N–F species. Given that Selectfluor is sufficiently electrophilic to react with the neutral enol forms of dicarbonyls **1a**–**i**, we believe that *N*-fluoroammonium ion **6** (Scheme 3) reacts with **1a**–**i**-enol, whereas fluorine does not appear to react with neutral **1a**–**i**-enol to give 2-fluoro products **2a**–**i**. Conversely, fluorine could react directly with the anionic **1a**–**i**-enolate species in parallel with *N*-fluoroammonium ion **6**. Fluoride ions formed through the reactions between fluorine and quinuclidine or fluorine and enolate species, may deprotonate **1a**–**i**-enol, to form enolates of **1a**–**i** that are reactive towards both fluorine and *N*-fluoroammonium ion **6**. The fluorination of **1a**–**i** affords monofluoro products **2a**–**i** in their keto tautomeric forms. For difluorodiketones **3a**–**i** to be formed, enolization of **2a**–**i**-keto must occur through deprotonation at the 2-position, and this process is a key limiting factor [17]. The challenge posed by enolization of **2a**–**i**-keto may be estimated from p*K*_a_ differences between acidic species and potential base species. The p*K*_a_(MeCN) for dibenzoylmethane (**1a**) can be estimated from p*K*_a_(DMSO) [50], where p*K*_a_(MeCN) = p*K*_a_(DMSO) + 12.9 = 13.4 + 12.9 = 26.3. Mayr and co-workers have shown the 2-fluoro-substituted species to be only slightly less acidic than their non-fluorinated homologues owing to the dominant π-donor effect of the 2-fluoro group [51–52]. On this basis, quinuclidine with p*K*_aH_(MeCN) ≈ 18.0–19.5 (estimated using p*K*_aH_(water) = 11.0 and p*K*_aH_(DMSO) = 9.8), is not predicted to be sufficiently basic to offer significant acceleration of the enolization processes of residual **1a**–**i**-keto or, more critically, the 2-fluoro-keto intermediates **2a**–**i**-keto that are formed after monofluorination [50,53–54]. Consequently, we believe a stronger base must be formed during the fluorination process in the presence of quinuclidine, and it is this base that accelerates enolization of **2a**–**i**-keto to allow difluorination to occur. The fluoride ion is a relatively strong base (p*K*_a_(MeCN) of HF is ≈25 based on p*K*_a_(DMSO) [55–56]), especially when formed in situ under anhydrous conditions, where solvation of fluoride ion is not possible. Since the p*K*_a_(MeCN) of **1a**-keto is ≈26.3, and we expect a p*K*_a_(MeCN) of **2a**-keto to be similar in value [51–52], we suggest fluoride ion may be sufficiently basic to cause significant acceleration of the deprotonation of **2a**–**i**-keto and allow formation of **2a**–**i**-enolates, which then react rapidly with fluorine gas, or *N*-fluoroammonium ion **6**, to form difluoroketones **3a**–**i**. Quinuclidine hydrofluoride has independently been shown to be an effective form of soluble fluoride ion in a variety of carbon–fluorine bond-forming processes [57–58]. Enols are, in general, significantly more acidic than their isomeric keto forms, for example, the p*K*_a_(DMSO) of acetone is ≈26.5, whereas the p*K*_a_(DMSO) of acetone enol is ≈18.2 [59]. Thus, assuming a similar difference in p*K*_a_ values between **1a**-keto and **1a**-enol, we expect p*K*_a_(MeCN) of **1a**-enol to be ≈18. On this basis, quinuclidine with p*K*_aH_(MeCN) ≈ 18.0–19.5, could also be an effective base to facilitate the formation of **1a**-enolate from **1a**-enol and thus facilitate the initial monofluorination step by either fluorine or *N*-fluoroammonium ion **6**.

**Scheme 4:**
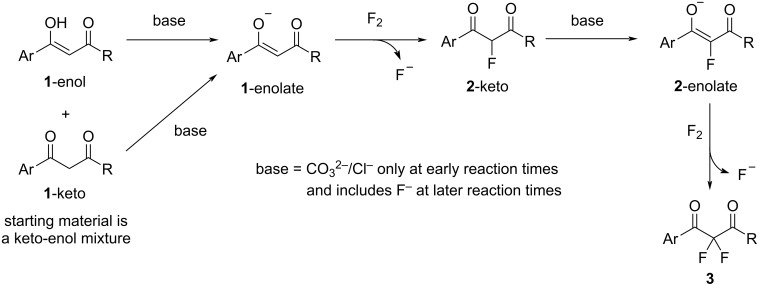
Proposed mechanisms of carbonate and chloride ion-mediated difluorination of 1,3-dicarbonyl substrates.

Carbonate ions are also expected to be highly basic in MeCN, however, their limited solubility is likely to inhibit their ability to act as an effective base for the formation of enolates of **1a** and **2a**, and this is reflected in the modest levels of formation of **3a** (Scheme 4). Chloride ion, on the other hand, is less basic (p*K*_a_(MeCN) of HCl is 10.30 [60]), however, its greater solubility seemingly allows some level of deprotonation of **1a**-enol to occur, where the enolate of **1a** can react with fluorine to afford **2a** and fluoride ion (Scheme 4). The resulting fluoride ion can then act as an additional, stronger base catalyst to facilitate further enolization processes and thus form **3a**. Similar arguments are also applicable to the fluorinations of ethyl benzoylacetate derivatives **4a**–**g**.

## Conclusion

From our experiments, we conclude that quinuclidine is the most effective mediating agent for the difluorination of 1,3-dicarbonyl species using fluorine. Difluorinations of 1,3-diketones **1** and 1,3-ketoesters **4** were achieved by the addition of two equivalents of quinuclidine. We propose that the fluoride ion, generated in situ, deprotonates enolic forms of 1,3-dicarbonyls and accelerates the rate-limiting enolization of 2-fluoro-1,3-dicarbonyl intermediates. The resulting enolates are nucleophilic and could react with fluorine or in situ-generated *N*-fluoroammonium ion **7** to form 2-fluoro- and 2,2-difluoro-1,3-dicarbonyl products.

## Supporting Information

Associated CDCC submission numbers: 2288841–2288848.

File 1 Experimental procedures, characterization data, and copies of ^1^H, ^19^F and ^13^C{^1^H} NMR spectra.

## Data Availability

All data that supports the findings of this study are available in the published article and/or the supporting information to this article.
