# Supplementary material for: Synthesis of 2,2-difluoro-1,3-diketone and 2,2-difluoro-1,3-ketoester derivatives using fluorine gas
Source: Beilstein J Org Chem. 2024 Feb 28;20:460–9. doi: 10.3762/bjoc.20.41 (PMC10910478; doi:10.3762/bjoc.20.41)
Supplement: File 1 — Experimental procedures, characterization data, and copies of 1H, 19F and 13C{1H} NMR spectra. [file Beilstein_J_Org_Chem-20-460-s001.pdf]

BEILSTEIN JOURNAL OF ORGANIC CHEMISTRY

# Supporting Information

for

# Synthesis of 2,2-difluoro-1,3-diketone and 2,2-difluoro-1,3-ketoester derivatives using fluorine gas

Alexander S. Hampton, David R. W. Hodgson, Graham McDougald, Linhua Wang and Graham Sandford

*Beilstein J. Org. Chem.* **2024,** *20,* 460–469. doi:10.3762/bjoc.20.41

# Experimental procedures, characterization data, and copies of $^{1}H$, $^{19}F$ and $^{13}C\{^{1}H\}$ NMR spectra

License and Terms: This is a supporting information file under the terms of the Creative Commons Attribution License (https://creativecommons.org/licenses/by/4.0). Please note that the reuse, redistribution and reproduction in particular requires that the author(s) and source are credited and that individual graphics may be subject to special legal provisions.
The license is subject to the *Beilstein Journal of Organic Chemistry* terms and conditions: (https://www.beilstein-journals.org/bjoc/terms)

# Table of contents


1 Experimental ........ S3

1.1 General experimental and instrumentation ........ S3

1.2 Synthesis of **1b–i** ........ S4

General procedure 1 (GP1)—synthesis of compounds **1b–h** ........ S4

1.2.1 1-(4-Methylphenyl)-3-phenylpropane-1,3-dione (**1b**) ........ S4

1.2.2 1-(4-Methoxyphenyl)-3-phenylpropane-1,3-dione (**1c**) ........ S5

1.2.3 1-(4-Chlorophenyl)-3-phenylpropane-1,3-dione (**1d**) ........ S5

1.2.4 1-(4-Fluorophenyl)-3-phenylpropane-1,3-dione (**1e**) ........ S6

1.2.5 1-(4-Nitrophenyl)-3-phenylpropane-1,3-dione (**1f**) ........ S7

1.2.6 1,3-Bis(4-chlorophenyl)-propane-1,3-dione (**1g**) ........ S7

1.2.7 1,3-Bis(4-fluorophenyl)-propane-1,3-dione (**1h**) ........ S8

1.2.8 1,3-Bis(4-nitrophenyl)-propane-1,3-dione (**1i**) ........ S8

1.3 Screening conditions for the base-mediated direct fluorination of dibenzoylmethane ........ S10

1.4 Screening conditions for the quinuclidine-mediated direct fluorination of dibenzoyl-methane ........ S11

1.5 Experimental to Table 2: Synthesis of **3a–i** ........ S12

General procedure 2 (GP2)—synthesis of **3a–i** ........ S12

1.5.1 2,2-Difluoro-1,3-diphenylpropane-1,3-dione (**3a**) ........ S12

1.5.2 2,2-Difluoro-1-(4-chlorophenyl)-3-phenylpropane-1,3-dione (**3d**) ........ S13

1.5.3 2,2-Difluoro-1-(4-fluorophenyl)-3-phenylpropane-1,3-dione (**3e**) ........ S13

1.5.3 2,2-Difluoro-1-(4-nitrophenyl)-3-phenylpropane-1,3-dione (**3f**) ........ S14

1.5.5 2,2-Difluoro-1,3-bis(4-chlorophenyl)propane-1,3-dione (**3g**) ........ S14

1.5.6 2,2-Difluoro-1,3-bis(4-fluorophenyl)propane-1,3-dione (**3h**) ........ S15

1.5.7 2,2-Difluoro-1,3-bis(4-nitrophenyl)-propane-1,3-dione (**3i**) ........ S15

1.6 Synthesis of **4b–h** ........ S16

General procedure 3 (GP3)– synthesis of compounds **4b–h** ........ S16

1.6.1 Ethyl (4-methoxy)benzoylacetate (**4b**) ........ S16

1.6.2 Ethyl (4-chloro)benzoylacetate (**4c**) ........ S17

1.6.3 Ethyl (4-trifluoromethyl)benzoylacetate (**4d**) ........ S17

1.6.4 Ethyl (4-nitro)benzoylacetate (**4e**) ........ S18

1.6.5 Ethyl (4-cyano)benzoylacetate (**4f**) ........ S18

1.6.6 Ethyl (3-nitro)benzoylacetate (**4g**) ........ S19

1.6.7 Ethyl 3-oxo-3-(pyridin-4-yl)propanoate (**4h**) ........ S20

1.7 Screening vonditions for the base-mediated direct fluorination of ethyl benzoylacetate ........ S21

1.8 Synthesis of **5a–g** ........ S22

General procedure 4 (GP4) — synthesis of compounds **5a–g** ........ S22

1.8.1 Ethyl 2,2-difluoro-3-oxo-3-phenylpropanoate (**5a**) ........ S22

1.8.2 Ethyl 2,2-difluoro-3-(4-chlorophenyl)-3-oxo-propanoate (**5c**) ........ S23

1.8.3 Ethyl 2,2-difluoro-3-(4-(trifluoromethyl)phenyl)-3-oxo-propanoate (**5d**) ........ S23

1.8.4 Ethyl 2,2-difluoro-3-(4-(nitrophenyl)-3-oxo-propanoate (**5e**) ........ S24

1.8.5 Ethyl 2,2-difluoro-3-(4-(cyanophenyl)-3-oxo-propanoate (**5f**) ........ S24

1.8.6 Ethyl 2,2-difluoro-3-(3-(nitrophenyl)-3-oxo-propanoate (**5g**) ........ S25

2 NMR Spectra ........ S26

2.1 1-(Methylphenyl)-3-phenylpropane-1,3-dione (**1b**) ........ S26

2.2 1-(Methoxyphenyl)-3-phenylpropane-1,3-dione (**1c**) ........ S27

2.3 1-(Chlorophenyl)-3-phenylpropane-1,3-dione (**1d**) ........ S28
2.4 1-(Fluorophenyl)-3-phenylpropane-1,3-dione (**1e**) ........ S29
2.5 1-(Nitrophenyl)-3-phenylpropane-1,3-dione (**1f**) ........ S31
2.6 1,3-Bis(4-chlorophenyl)-propane-1,3-dione (**1g**) ........ S32
2.7 1,3-Bis(fluorophenyl)-propane-1,3-dione (**1h**) ........ S33
2.8 1,3-Bis(nitrophenyl)-propane-1,3-dione (**1i**) ........ S35
2.9 2,2-Difluorophenylpropane-1,3-dione (**3a**) ........ S36
2.10 2,2-Difluoro-1-(4-chlorophenyl)-3-phenylpropane-1,3-dione (**3d**) ........ S38
2.11 2,2-Difluoro-1-(4-fluorophenyl)-3-phenylpropane-1,3-dione (**3e**) ........ S40
2.12 2,2-Difluoro-1-(nitrophenyl)-3-phenylpropane-1,3-dione (**3f**) ........ S42
2.13 2,2-Difluoro-1,3-bis(chlorophenyl)propane-1,3-dione (**3g**) ........ S44
2.14 2,2-Difluoro-1,3-bis(fluorophenyl)propane-1,3-dione (**3h**) ........ S46
2.15 2,2-Difluoro-1,3-bis(nitrophenyl)propane-1,3-dione (**3i**) ........ S48
2.16 Ethyl (4-methoxy)benzoylacetate (**4b**) ........ S50
2.17 Ethyl (4-chloro)benzoylacetate (**4c**) ........ S51
2.18 Ethyl (4-trifluoromethyl)benzoylacetate (**4d**) ........ S52
2.19 Ethyl (4-nitro)benzoylacetate (**4e**) ........ S54
2.20 Ethyl (4-cyano)benzoylacetate (**4f**) ........ S55
2.21 Ethyl (3-nitro)benzoylacetate (**4g**) ........ S56
2.22 Ethyl 3-oxo-3-(pyridin-4-yl)propanoate (**4h**) ........ S57
2.23 Ethyl 2,2-difluoro-3-oxo-3-phenylpropanoate (**5a**) ........ S58
2.24 Ethyl 2,2-difluoro-3-(4-chlorophenyl)-3-oxopropanoate (**5c**) ........ S60
2.25 Ethyl 2,2-difluoro-3-(4-trifluoromethylphenyl)-3-oxopropanoate (**5d**) ........ S62
2.26 Ethyl 2,2-difluoro-3-(4-nitrophenyl)-3-oxopropanoate (**5e**) ........ S64
2.27 Ethyl 2,2-difluoro-3-(4-cyanophenyl)-3-oxopropanoate (**5f**) ........ S66
2.28 Ethyl 2,2-difluoro-3-(3-nitrophenyl)-3-oxopropanoate (**5g**) ........ S68
3 Single crystal X-ray crystallography ........ S70
3.1 Crystallographic data for **1h** (polymorph A) ........ S71
3.2 Crystallographic data for **1h** (polymorph B) ........ S72
3.3 Crystallographic data for **3a** ........ S73
3.4 Crystallographic data for **3f** ........ S74
3.5 Crystallographic data for **3i** ........ S75
3.6 Crystallographic data for **4c** ........ S76
3.7 Crystallographic data for **4g** ........ S77
3.8 Crystallographic data for **5e** ........ S78
4 References ........ S79

# 1 Experimental

## 1.1 General experimental and instrumentation

Chemicals were purchased from Fisher Scientific, Apollo Scientific, Fluorochem or Sigma Aldrich and, unless otherwise stated, were used without any further purification. All column chromatography was carried out using Silicagel LC60A (40-63 micron) purchased from Fluorochem. Proton, carbon and fluorine nuclear magnetic resonance spectra ($^{1}H$ NMR, $^{13}C\{^{1}H\}$ NMR and $^{19}F$ NMR) were recorded on Bruker 400 Ultrashield ($^{1}H$ NMR at 400 MHz; $^{13}C\{^{1}H\}$ NMR at 101 MHz; $^{19}F$ NMR at 376 MHz), Varian DD2-500 (H NMR at 500 MHz; $^{13}C\{^{1}H\}$ NMR at 126 MHz), Varian VNMR-600 Ultrashield ($^{1}H$ NMR at 600 MHz; $^{13}C\{^{1}H\}$ NMR at 151 MHz) spectrometers or a Varian VNMRS-700 ($^{1}H$ NMR at 700 MHz; $^{13}C\{^{1}H\}$ NMR at 176 MHz) spectrometer with residual solvent peaks as the internal standard. $^{1}H$, $^{13}C\{^{1}H\}$ and $^{19}F$ NMR spectroscopic data are reported as follows: chemical shift (ppm), integration, multiplicity (s = singlet, d = doublet, t = triplet, q = quartet, p = pentet, m = multiplet), coupling constant (Hz). Accurate mass analysis was achieved with a QtoF Premier mass spectrometer (Waters Ltd, UK) or an LCT Premier XE mass spectrometer (Waters Ltd, UK) equipped with an atmospheric solids analysis probe (ASAP). Infrared (IR) spectra were recorded on a Perkin Elmer FTIR Spectrum Two™ fitted with an ATR probe. Melting points were measured with a Gallenkamp apparatus at atmospheric pressure and are uncorrected.

Fluorinations were carried out in a borosilicate glass fluorination reactor (100 mL) unless otherwise stated. The reactor was built from a standard glass bottle with a GL 45 thread joint and a PTFE screw cap equipped with a gas inlet/outlet head built of stainless steel, PTFE and FEP Swagelok components. The flow rates were controlled with a Brooks Instrument gas mass flow controller.

## 1.2 Synthesis of 1b–i

### General procedure 1 (GP1)—synthesis of compounds 1b–h

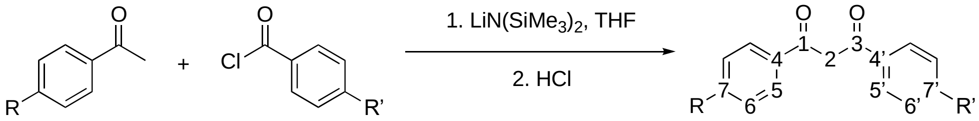


The acetophenone derivative (10 mmol), lithium hexamethyldisilizane (1 M in tetrahydrofuran, 20 mmol, 20 mL) and anhydrous tetrahydrofuran (20 mL) were stirred at −78 °C for 30 min under argon. The corresponding acid chloride (10 mmol) was added over the course of a few minutes, and the reaction mixture was allowed to warm to room temperature overnight and stirred for 48 h. Where the acid chloride was a solid, the amount was added to a round-bottomed flask, purged with argon, and dissolved in anhydrous tetrahydrofuran (10 mL) before being transferred to the reaction mixture via cannula. The reaction mixture was quenched with 36% hydrochloric acid (3 mL), then partitioned between water (10 mL) and ethyl acetate (30 mL). The aqueous layer was extracted with ethyl acetate (2 × 30 mL) and the combined organic phases were washed with sodium bicarbonate (30 mL) and water (30 mL). The organic layer was dried over magnesium sulphate and the solvent was removed under reduced pressure to yield the crude product, which was further purified by recrystallization or column chromatography, if required.

### 1.2.1 1-(4-Methylphenyl)-3-phenylpropane-1,3-dione (1b)

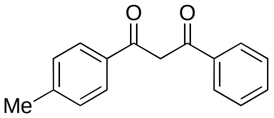

Prepared according to **GP1**, 4-methylacetophenone (1.34 mL, 10.04 mmol) and benzoyl chloride (1.16 mL, 9.99 mmol) after purification by column chromatography with ethyl acetate 15% v/v in hexane solvent mixture as the eluent gave a mixture of keto- and enol-tautomers of *1-(4-methylphenyl)-3-phenyl-1,3-propanedione* (2.408 g, 100%) as a white solid; Mp 85–86 °C [lit. Mp 84–85 °C[1]; 86-89 °C[2]]. *Enol tautomer* (92%) $^1$H NMR (700 MHz, CDCl$_3$) δ 2.44 (3 H, s, C**8**H$_3$), 6.84 (1 H, s, C**2**H), 7.28–7.31 (2 H, m, C**6**H), 7.47–7.51 (2 H, m, C**6’**H), 7.53–7.57 (1 H, m, C**7’**H), 7.88–7.92 (2 H, m, C**5**H), 7.97–7.99 (2 H, m, C**5’**H); $^{13}$C{$^1$H} NMR (176 MHz, CDCl$_3$) δ 21.8 (C**8**), 93.0 (C**2**), 127.2 (C**5’**), 127.4 (C**5**), 128.8 (C**6’**), 129.6 (C**6**), 132.4 (C**7’**), 133.0 (C**4**), 135.8 (C**4’**), 143.4 (C**7**), 185.3 (C**3**), 186.2 (C**1**); *ketone tautomer* (8%) $^1$H NMR (700 MHz, CDCl$_3$)

δ 4.61 (2H, s, C**2**H), 2.42 (3 H, s, - C**8**H); *m/z* ($EI^+$) 238.2 ($[M]^+$, 92%), 223.1 ($[C_{15}H_{11}O_2]^+$, 11%), 161.1 ($[C_{10}H_9O_2]^+$, 22%), 147.1 ($[C_9H_7O_2]^+$, 16%), 119.1 ($[C_8H_7O]^+$, 87%), 105.1 ($[C_7H_5O]^+$, 44%), 91.1 ($[C_7H_7]^+$, 43%), 77.1 ($[C_6H_5]^+$, 39%), 69.0 ($[C_5H_9]^+$, 59%). HRMS (ASAP) *m/z* calculated for $[M+H]^+$ $C_{16}H_{15}O_2$ 239.1072; found 239.1067. IR (neat, $cm^{-1}$) 2923, 1459, 1185, 1018, 767, 687, 582. Crystals suitable for X-ray diffraction were grown by slow evaporation from hexane. Data consistent with those previously reported in the literature.[2]

### 1.2.2 1-(4-Methoxyphenyl)-3-phenylpropane-1,3-dione (1c)

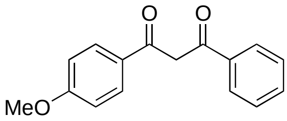


Prepared according to **GP1**, acetophenone (1.17 mL, 10.03 mmol) and 4-methoxybenzoyl chloride (1.35 mL, 9.97 mmol) after recrystallisation from ethyl acetate afforded a mixture of keto- and enol-tautomers of *1-(4-methoxyphenyl)-3-phenyl-1,3-propanedione* (2.399 g, 95%) as colourless crystals; Mp 127–128 °C [lit. Mp 127–128 °C;[3] 128 °C;[4] 129 °C[1]]. *Enol tautomer* (90%) $^1$H NMR (700 MHz, $CDCl_3$) δ 3.89 (3 H, s, C**8**$H_3$), 6.80 (1 H, s, C**2**H), 6.97–7.00 (2 H, m, C**6**H), 7.47–7.50 (2 H, m, C**6’**H), 7.53–7.56 (1 H, m, C**7’**H), 7.96–7.98 (2 H, m, C**5’**H), 7.98–7.99 (2 H, m, C**5**H); $^{13}$C{$^1$H} NMR (176 MHz, $CDCl_3$) δ 55.6 (C**8**), 92.5 (C**2**), 114.1 (C**6**), 127.1 (C**5’**), 128.4 (C**4**), 128.8 (C**6’**), 129.5 (C**5**), 132.3 (C**7’**), 135.7 (C**4’**), 163.4 (C**7**), 184.2 (C**3**), 186.3 (C**1**); *ketone tautomer* (10%) $^1$H NMR (700 MHz, $CDCl_3$) δ 4.59 (2H, s, C**2**H), 3.87 (3 H, s, -C**8**H); *m/z* ($EI^+$) 254.1 ($[M^+]$, 100%), 177.1 ($[C_{10}H_9O_3]^+$, 19%), 135.1 ($[C_8H_7O_2]^+$, 95%), 108.1 ($[C_7H_8O]^+$, 60%), 105.1 ($[C_7H_5O]^+$, 30%), 77.1 ($[C_6H_5]^+$, 56%), 69.0 ($[C_5H_9]^+$, 31%), 51.1 ($[C_4H_3]^+$, 9%). HRMS (ASAP) *m/z* calculated for $[M+H]^+$ $C_{16}H_{15}O_3$ 255.1021; found 255.1024. IR (neat $cm^{-1}$) 2970, 1452, 1298, 1182, 1020, 843, 766, 582, 502. Data consistent with those previously reported in the literature.[3-5]

### 1.2.3 1-(4-Chlorophenyl)-3-phenylpropane-1,3-dione (1d)

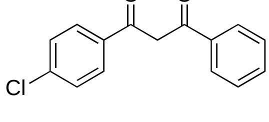


Prepared according to **GP1**, acetophenone (1.17 mL, 10.03 mmol) and 4-chlorobenzoyl chloride (1.28 mL, 9.98 mmol) after recrystallisation from hexane afforded a mixture of keto- and enol-tautomers of *1-(4-chlorophenyl)-3-phenyl-1,3-propanedione* (2.314 g, 90%) as a pale yellow solid; Mp 85–87 °C [lit. Mp 87 °C;[4] 86.9-87.6°C;[5b] 86–88 °C[6]].

*Enol tautomer* (94%) $^1$H NMR (600 MHz, $CDCl_3$) δ 6.81 (1 H, s, C**2**H), 7.47 (2 H, dt, $^3J_{HH}$ 8.7, $^4J_{HH}$ 2.4, C**6**H), 7.48–7.51 (2 H, m, C**5'**H), 7.57 (1 H, tt, $^3J_{HH}$ 7.4, $^4J_{HH}$ 1.4, C**7'**H), 7.93 (2 H, dt, $^3J_{HH}$ 8.7, $^4J_{HH}$ 2.4, C**5**H), 7.97–8.00 (2 H, m, C**6'**H); $^{13}$C{$^1$H} NMR (151 MHz, $CDCl_3$) δ 93.2 (C**2**), 127.3 (C**6'**), 128.7 (C**5**), 128.9 (C**5'**), 129.1 (C**6**), 132.8 (C**7'**), 134.1 (C**4**), 135.5 (C**4'**), 138.9 (C**7**), 184.7 (C**1**), 185.9 (C**3**); *ketone tautomer* (6%) $^1$H NMR (600 MHz, $CDCl_3$) δ 4.61 (2H, s, C**2**H); *m/z* (EI$^+$) 258.1 ([M]$^+$, 79%), 181.0 ([$C_9H_6ClO_2$]$^+$, 22%), 147.1 ([$C_9H_7O_2$]$^+$, 21%), 139.1 ([$C_7H_4ClO$]$^+$, 43%), 111.1 ([$C_6H_4Cl$]$^+$, 20%), 105 ([$C_7H_5O$]$^+$, 56.7%), 77.1 ([$C_6H_5$]$^+$, 96%). HRMS (ASAP) *m/z* calculated for [M+H]$^+$ $C_{15}H_{12}ClO_2$ 259.0526; found 259.0531. IR (neat cm$^{-1}$) 2924, 1516, 1282, 1093, 1012, 843, 757, 682. Data consistent with those previously reported in the literature.[4-5]

### 1.2.4 1-(4-Fluorophenyl)-3-phenylpropane-1,3-dione (1e)

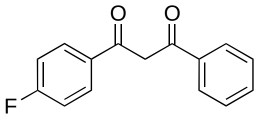

Prepared according to **GP1**, 4-fluoroacetophenone (1.21 mL, 9.97 mmol) and benzoyl chloride (1.16 mL, 9.99 mmol) after recrystallisation from hexane afforded a mixture of keto- and enol-tautomers of *1-(4-fluorophenyl)-3-phenyl-1,3-propanedione* (2.417 g, 100%) as a beige solid; Mp 78–79 °C [lit. Mp 88 °C[7]]. *Enol tautomer* (93%) $^1$H NMR (700 MHz, $CDCl_3$) δ 6.81 (1 H, s, C**2**H), 7.14–7.20 (2 H, m, C**6**H), 7.47–7.52 (2 H, m, C**5'**H), 7.56 (1 H, tt, $^3J_{HH}$ 7.4, $^4J_{HH}$ 1.2, C**7'**H), 7.97–7.99 (2 H, m, C**6'**H), 8.00–8.02 (2 H, m, C**5**H); $^{19}$F NMR (376 MHz, $CDCl_3$) δ − 106.2 (tt, $^3J_{HF}$ 8.4, $^4J_{HF}$ 5.4); $^{13}$C{$^1$H} NMR (176 MHz, $CDCl_3$) δ 93.0 (C**2**), 116.0 (d, $^2J_{CF}$ 21.9, C**6**), 127.3 (C**6'**), 128.9 (C**5'**), 129.8 (d, $^3J_{CF}$ 9.1, C**5**), 132.6 (C**7'**), 135.4 (C**4'**), 165.6 (d, $^1J_{CF}$ 253.9, C**7**), 185.26 (C**1**) 185.27 (C**3**); *ketone tautomer* (7%) $^1$H NMR (700 MHz, $CDCl_3$) δ 4.61 (2H, s, C**2**H); $^{19}$F NMR (376 MHz, $CDCl_3$) δ −103.9 (tt, $^3J_{HF}$ 8.3, $^4J_{HF}$ 5.3); *m/z* (EI$^+$) 242.0 ([M]$^+$, 19%), 223.1 ([$C_{15}H_{11}O_2$]$^+$, 3%), 147.1 ([$C_9H_7O_2$]$^+$, 10%), 123.1 ([$C_7H_4FO$]$^+$, 10%), 119.1 ([$C_8H_7O$]$^+$, 4%), 105.1 ([$C_7H_5O$]$^+$, 20%), 95.0 ([$C_6H_4F$]$^+$, 10%), 91.1 ([$C_7H_7$]$^+$, 7%), 77.0 ([$C_6H_5$]$^+$, 29%), 69.0 ([$C_5H_9$]$^+$, 15%), 51.0 ([$C_4H_3$]$^+$, 12%). HRMS (ASAP) *m/z* calculated for [M+H]$^+$ $C_{15}H_{12}FO_2$ 243.0821; found 243.0826. IR (neat cm$^{-1}$) 3112, 1729, 1520, 1348, 1284, 1108, 955, 764, 688, 562. Data consistent with those previously reported in the literature.[7]

### 1.2.5 1-(4-Nitrophenyl)-3-phenylpropane-1,3-dione (1f)

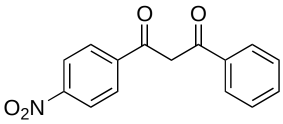


Prepared according to **GP1**, acetophenone (1.17 mL, 10.03 mmol) and 4-nitrobenzoyl chloride (1.860 g, 10.02 mmol) without further purification gave a mixture of keto- and enol-tautomers of *1-(4-nitrophenyl)-3-phenyl-1,3-propanedione* (2.677 g, 99%) as a yellow solid; Mp 145–146 °C [lit. Mp 160–161 °C[1]]; *enol tautomer* (95%) $^{1}$H NMR (700 MHz, $CDCl_3$) δ 6.90 (1 H, s, C**2**H), 7.51–7.54 (2 H, m, C**5'**H), 7.59–7.62 (1 H, C**5**H), 8.00–8.03 (2 H, m, C**6'**H), 8.14 (2 H, dt, *J* 9.0, 2.3, C**6**H), 8.34 (2 H, dt, *J* 9.0, 2.3, C**5**H); $^{13}$C{$^{1}$H} NMR (176 MHz, $CDCl_3$) δ 94.4 (C**2**), 124.0 (C**2**), 127.6 (C**6'**), 128.2 (C**6**), 129.0 (C**5'**), 133.3 (C**7'**), 135.3 (C**4'**), 141.1 (C**2**), 150.1 (C**7**), 181.8 (C**1**), 188.0 (C**3**); *ketone tautomert* (5%) $^{1}$H NMR (700 MHz, $CDCl_3$) δ 4.69 (2 H, s, C**2**H); *m/z* ($EI^+$) 269.1 ($[M]^+$, 77%), 222.1 ($[C_{15}H_{10}O_2]^+$, 15%), 192.0 ($[C_9H_6NO_4]^+$, 17%), 147.1 ($[C_9H_7O_2]^+$, 24%), 120.1 ($[C_8H_8O]^+$, 7%), 105.1 ($[C_7H_5O]^+$, 76%), 91.1 ($[C_7H_7]^+$, 4%), 77.1 ($[C_6H_5]^+$, 49%), 69.0 ($[C_5H_9]^+$, 51%), 51.0 ($[C_4H_3]^+$, 11%). HRMS (ASAP) *m/z* calculated for $[M+H]^+$ $C_{15}H_{12}NO_4$ 270.0766; found 270.0757. IR (neat cm-1) 3121, 2981, 1692, 1513, 1343, 1103, 855, 744. Data consistent with that previously reported in the literature.[5a]

### 1.2.6 1,3-Bis(4-chlorophenyl)-propane-1,3-dione (1g)

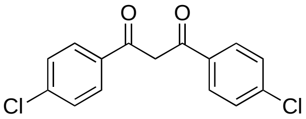


Prepared according to GP1, 4-chloroacetophenone (1.30 mL, 10.03 mmol) and 4-chlorobenzoyl chloride (1.28 mL, 9.98 mmol) after recrystallisation from hexane and ethyl acetate afforded a mixture of keto- and enol-tautomers of *1,3-bis(4-chlorophenyl)propane-1,3-dione* (2.882 g, 99%) as a very pale yellow solid; Mp 151–153 °C [lit. Mp 160.5-161.9 °C;[5b] 160–161 °C]. *Enol tautomer* (94%) $^{1}$H NMR (700 MHz, $CDCl_3$) δ 6.77 (1 H, s, C**2**H), 7.46–7.48 (4 H, m, C**4**H), 7.91–7.93 (4 H, m, C**5**H); $^{13}$C{$^{1}$H} NMR (176 MHz, $CDCl_3$) δ 93.0 (C**2**), 128.7 (C**4**), 129.2 (C**5**), 133.9 (C**6**), 139.1 (C**3**), 184.8 (C**1**); *ketone tautomer* (6%) $^{1}$H NMR (700 MHz, $CDCl_3$) δ 4.58 (2 H, s, C**2**H); *m/z* ($EI^+$) 292.1 ($[M]^+$, 68%), 223.1 ($[C_{15}H_{11}O_2]^+$, 11%), 181.0 ($[C_9H_6ClO_2]^+$, 40%), 139.1 ($[C_7H_4ClO]^+$, 100%), 111.1 ($[C_6H_4Cl]^+$, 54%), 69.0 ($[C_5H_9]^+$, 54%). HRMS (ASAP) *m/z* calculated for $[M+H]^+$ $C_{15}H_{11}O_2Cl_2$ 293.0136; found 293.0147. IR (neat cm–

$^{1}$) 2981, 1582, 1503, 1397, 1279, 1177, 970, 775. Data consistent with those previously reported in the literature.[5b]

### 1.2.7 1,3-Bis(4-fluorophenyl)-propane-1,3-dione (1h)

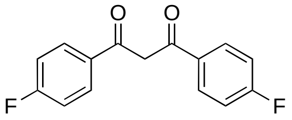

Prepared according to **GP1**, 4-fluoroacetophenone (1.21 mL, 9.97 mmol) and 4-fluorobenzoyl chloride (1.18 mL, 9.99 mmol) after recrystallisation from hexane and ethyl acetate afforded a mixture of keto- and enol-tautomers of *1,3-bis(4-fluorophenyl)propane-1,3-dione* (2.099 g, 81%) as a beige solid; Mp 107–108 °C [lit. Mp 109 °C;[8] 137.8-139.1 °C[5b]]. *Enol tautomer* (92%) $^{1}$H NMR (700 MHz, $CDCl_3$) δ 6.74 (1 H, s, C**2**H), 7.14–7.20 (4 H, m, C**5**H), 7.98–8.02 (4 H, m, C**4**H); $^{19}$F NMR (376 MHz, $CDCl_3$) δ −106.1 (tt, $^{3}J_{HF}$ 8.4, $^{4}J_{HF}$ 5.4); $^{13}$C{$^{1}$H} NMR (176 MHz, $CDCl_3$) δ 92.6 (C**2**), 116.0 (d, $^{2}J_{CF}$ 21.9, C**5**), 129.7 (d, $^{3}J_{CF}$ 9.2, C**4**), 131.8 (d, $^{4}J_{CF}$ 3.1, C**3**), 165.6 (d, $^{1}J_{CF}$ 254.1, C**6**), 184.6 (s, C**1**); *ketone tautomer* (8%) $^{1}$H NMR (700 MHz, $CDCl_3$) δ 4.58 (2 H, s, C**2**H); $^{19}$F NMR (376 MHz, $CDCl_3$) δ –103.6 (tt, $^{3}J_{HF}$ 8.3, $^{4}J_{HF}$ 5.3); *m/z* ($EI^+$) 260.1 ($[M]^+$, 58%), 223.1 ($[C_{15}H_{11}O_2]^+$, 11%), 165.1 ($[C_9H_6FO_2]^+$, 30%), 123.1 ($[C_7H_4FO]^+$, 100%), 95.1 ($[C_6H_4F]^+$, 47%), 69.0 ($[C_5H_9]^+$, 32%). HRMS (ASAP) *m/z* calculated for $[M+H]^+$. $C_{15}H_{11}O_2F_2$ 293.0727; found 261.0718. Crystals suitable for X-ray diffraction were grown by slow evaporation from hexane—see section 3.1 and 3.2. IR (neat $cm^{-1}$) 3082, 1596, 1467, 1301, 1221, 1012, 859, 856, 784, 505. Data consistent with those previously reported in the literature.[5b]

### 1.2.8 1,3-Bis(4-nitrophenyl)-propane-1,3-dione (1i)

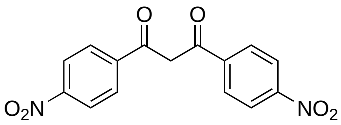

4-Nitroacetophenone (1.648 g, 9.97 mmol) and $LiN(SiMe_3)_2$ (1 M in tetrahydrofuran, 20 mmol, 20 mL, 2 equiv) in anhydrous tetrahydrofuran (20 mL) were stirred at −78 °C for 30 min under argon. 4-Nitrobenzoyl chloride (1.856 g, 10.0 mmol) was dissolved in anhydrous tetrahydrofuran (10 mL) and added to the reaction mixture which was left to stir for 48 hours. The reaction mixture was quenched with 36% hydrochloric acid (3 mL) before partitioned between water (10 mL) and dichloromethane (500 mL). The aqueous layer was extracted with dichloromethane (2 × 50 mL) and the combined organic phases

washed with sodium bicarbonate (30 mL) and water (20 mL). The organic layer was dried over magnesium sulphate and the solvent removed under reduced pressure to yield the crude product. Recrystallisation from acetone gave the enol tautomer of *1,3-(4-nitrophenyl)propane-1,3-dione* (3.098 g, 99%) as a yellow solid; Mp 240–242 °C [lit. Mp 249.1-250.9 °C[5b]]. *Enol tautomer* (100%) $^{1}$H NMR (700 MHz, $CDCl_3$) δ 6.93 (1 H, s, C**2**H), 8.15–8.18 (4 H, m, C**5**H), 8.35–8.38 (4 H, m, C**4**H); $^{13}$C{$^{1}$H} NMR (176 MHz, $CDCl_3$) δ 95.2 (C**2**), 124.2 (C**4**), 128.5 (C**5**), 140.5 (C**3**), 150.4 (C**6**), 184.1 (C**1**); *m/z* ($ES^–$) 313.2 ($[M-H]^-$, 100%. HRMS (ASAP) *m/z* calculated for $[M-H]^+$. $C_{15}H_9NO_4$ 313.0461; found 313.0434. IR (neat $cm^{–1}$) 3095, 1511, 1344, 1224, 1093, 1009, 855, 747, 450. Data consistent with those previously reported in the literature.[5b, 9]

## 1.3 Screening conditions for the base-mediated direct fluorination of dibenzoylmethane

Dibenzoylmethane (0.449 g, 2.00 mmol) and the mediating agent were added to a SIMAX glass bottle and dissolved in acetonitrile (20 mL). The reaction vessel was cooled to 0 °C, stirred rapidly and purged with nitrogen for 10 minutes before fluorine gas, as a 10% mixture in nitrogen (v/v) was passed through the reaction mixture at a prescribed flow rate, (15 mL $min^{-1}$) that was controlled by a mass flow controller. After purging with nitrogen for 10 minutes, the reaction vessel was disconnected and the solvent removed under reduced pressure. A known mass of α,α,α-trifluorotoluene was added as a reference standard, and the organic products were analysed by NMR spectroscopy.

**Table S1:** Screening conditions for the base mediated fluorination of DBM

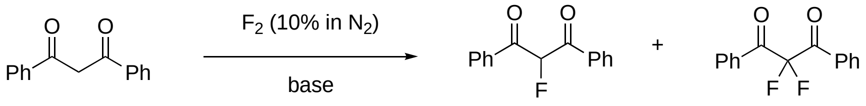


**1a** **2a** **3a**

| Entry | Base | Equiv. of base | Equiv. of $F_2$ | Yield by $^{19}F$ NMR | | |
|---|---|---|---|---|---|---|
| | | | | **1a**/% | **2a**/% | **3a**/% |
| 1 | - | - | 1 | 100 | 0 | 0 |
| 2 | - | - | 20 | Polyfluorinated Tar | | |
| 3 | DABCO | 1 | 1 | 32 | 4 | 20 |
| 4 | DABCO | 1 | 2 | 1 | 1 | 37 |
| 5 | DABCO | 1 | 3 | Polyfluorinated tar | | |
| 6 | DABCO | 2 | 2 | Many fluorinated products | | |
| 7 | DABCO | 0.1 | 1 | 22 | 28 | 8 |
| 8 | Quinuclidine | 1 | 1 | 42 | 10 | 43 |
| 9 | Quinuclidine | 1.2 | 1 | 54 | 1 | 43 |
| 10 | $Et_3N$ | 1 | 1 | 56 | 25 | 6 |
| 11 | $Cs_2CO_3$ | 1 | 1 | 0 | 4 | 14 |
| 12 | NaCl | 1 | 1 | 0 | 33 | 12 |

*NMR yield calculated by comparing the integrals (CF dp at −189.9 ppm, $CF_2$ s at −102.7 ppm) to a known amount of α,α,α-trifluorotoluene.*

## 1.4 Screening conditions for the quinuclidine-mediated direct fluorination of dibenzoylmethane

Dibenzoylmethane (0.449 g, 2.00 mmol) and quinuclidine were added to a SIMAX glass bottle and dissolved in acetonitrile (20 mL). The reaction vessel was cooled to 0 °C, stirred rapidly and purged with nitrogen for 10 minutes before fluorine gas, as a 10% mixture in nitrogen (*v/v*) was passed through the reaction mixture at a prescribed flow rate, (15 mL $min^{-1}$) that was controlled by a mass flow controller. After purging with nitrogen for 10 minutes, the reaction vessel was disconnected and the solvent removed under reduced pressure. The organic products were analysed by NMR spectroscopy as described above.

**Table S2:** Screening conditions for the quinuclidine mediated direct fluorination of DBM

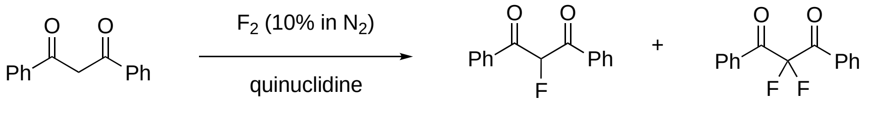


**1a** **2a** **3a**

| Entry | Equiv. of quinuclidine | Equiv. of $F_2$ | Ratio of products by NMR | | |
|---|---|---|---|---|---|
| | | | **1a** | **2a** | **3a** |
| 1 | 1 | 1 | 3 | 2 | 4 |
| 2 | 1.1 | 2.1 | 1 | 5 | 47 |
| 3 | 1.1 | 2.3 | 1 | 16 | 120 |

## 1.5 Experimental to Table 2: Synthesis of 3a–i

### General procedure 2 (GP2)—synthesis of 3a–i

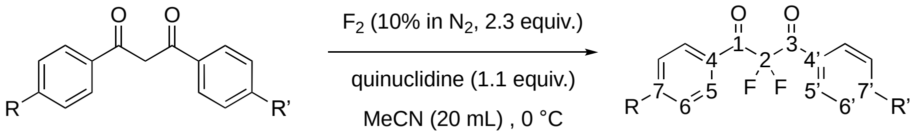


The corresponding 1,3-diketone (2 mmol, 1 equiv) and quinuclidine (0.245 g, 2.2 mmol, 1.1 equiv) were added to a SIMAX glass bottle and dissolved in acetonitrile (20 mL). The reaction vessel was cooled to 0 °C, stirred rapidly and purged with nitrogen for 10 minutes before fluorine, as a 10% mixture in nitrogen (v/v), was passed through the reaction mixture at a prescribed flow rate, (4.6 mmol, 2.3 equiv, 15 mL min$^{-1}$) that was controlled by a mass flow controller for 75 min. After purging with nitrogen for 20 minutes, the reaction vessel was removed, and the solvent diluted with water (20 mL) and dichloromethane (90 mL). The two layers were separated, and the aqueous layer was extracted with dichloromethane (3 × 30 mL). The organic fractions were combined, washed with saturated sodium bicarbonate solution (20 mL) and dried over magnesium sulphate. The solvent was removed under reduced pressure to yield the crude product which was purified by column chromatography or recrystallisation.

### 1.5.1 2,2-Difluoro-1,3-diphenylpropane-1,3-dione (3a)

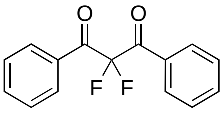

Prepared according to **GP2**, 1,3-diphenyl-1,3-propanedione (0.448 g, 2.00 mmol) after column chromatography with hexane/dichloromethane (1:1) as the eluent yielded *2,2-difluroro-1,3-diphenyl-1,3-propanedione* (0.338 g, 65%) as a white solid; Mp 56– 57 °C [lit. Mp 56–57 °C[10]]. $^1$H NMR (600 MHz, CDCl$_3$) δ 7.50 (4 H, td, $^3J_{HH}$, $^4J_{HH}$ 2.0, C**6**H), 7.62–7.68 (2 H, m, C**7**H), 8.09 (4 H, d, $^3J_{HH}$ 7.4, C**5**H); $^{19}$F NMR (376 MHz, CDCl$_3$) δ −102.7 (s); $^{13}$C{$^1$H} NMR (151 MHz, CDCl$_3$) δ 112.8 (t, $^1J_{CF}$ 265.8, C**2**), 129.1 (C**6**), 130.4 (t, $^4J_{CF}$ 2.7, C**5**), 135.2 (C**7**), 187.5 (t, $^2J_{CF}$ 27.0, C**1**); *m/z* (EI$^+$) 105.2 ([$C_7H_5O_2$]$^+$, 87%), 77.1 ([$C_6H_5$]$^+$, 43%). HRMS (ASAP) *m/z* calculated for [M+H]$^+$ $C_{15}H_{11}F_2O_2$ 261.0727; found 261.0723. Crystals suitable for X-ray diffraction were grown by slow evaporation from hexane, see section 3.3. IR (neat cm$^{-1}$) 3073, 1694, 1594, 1449, 1251, 1135, 887, 679. 523. Data consistent with those previously reported in the literature.[10,11]

### 1.5.2 2,2-Difluoro-1-(4-chlorophenyl)-3-phenylpropane-1,3-dione (3d)

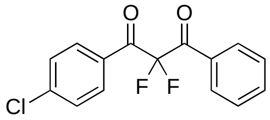


Prepared according to **GP2**, 1-(4-chlorophenyl)-3-phenyl-1,3-propanedione (0.514 g, 1.99 mmol) ) after column chromatography with hexane and dichloromethane (1:1) as the eluent yielded *2,2-diflurorо-1-(4-chlorophenyl)-3-phenyl-1,3-propanedione* (0.352 g, 60%) as a white solid; Mp 63–64 °C. $^1$H NMR (700 MHz, $CDCl_3$) δ 7.44–7.49 (2 H, m, C**6**H), 7.50–7.53 (2 H, m, C**6'**H), 7.67 (1 H, tt, $^3J_{HH}$ 7.5, $^4$JHH 1.2, C**7'**H), 8.03 (2 H, d, $^3J_{HH}$ 8.8, C**5**H), 8.08 (2 H, dd, $^3J_{HH}$ 8.5, $^4J_{HH}$ 1.0, C**5'**H); $^{19}$F NMR (376 MHz, $CDCl_3$) δ −102.7 (p, $^5J_{HF}$ 0.9); $^{13}$C{$^1$H} NMR (176 MHz, $CDCl_3$) δ 112.8 (t, $^1J_{CF}$ 266.0, C**2**), 129.1 (C**6'**), 129.5 (C**6**), 130.1 (C**4**), 130.4 (t, $^4J_{CF}$ 2.7, C**5'**), 131.6 (C**4'**), 131.8 (t, *J* 2.8, C**5**), 135.3 (C**7'**), 142.0 (C**7**), 186.5 (t, $^2J_{CF}$ 27.1, C**1**), 187.4 (t, $^2J_{CF}$ 26.9, C**3**); *m/z* (EI$^+$) 294.1 ([M]$^+$, 1%), 139.1 ([$C_7H_4ClO$]$^+$, 62%), 77.1 ([$C_6H_5$]$^+$, 96%), 51.1 ([$C_3H_4$]$^+$, 12%). HRMS (ASAP) *m/z* calculated for [M+H]$^+$ $C_{15}H_{10}ClF_2O_2$ 295.0337; found 295.0335. IR (neat cm$^{-1}$) 1692, 1588, 1449, 1250, 1138, 884, 682, 561.

### 1.5.3 2,2-Difluoro-1-(4-fluorophenyl)-3-phenylpropane-1,3-dione (3e)

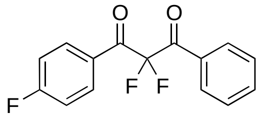


Prepared according to **GP2**, 1-(4-fluorophenyl)-3-diphenyl-1,3-propanedione (0.484 g, 2.00 mmol) after column chromatography with hexane and dichloromethane (1:1) as the eluent yielded *2,2-diflurorо-1-(4-fluorophenyl)-3-phenyl-1,3-propanedione* (0.327 g, 59%) as a white solid; Mp 36–37 °C. $^1$H NMR (700 MHz, $CDCl_3$) δ 7.16–7.19 (2 H, m, C**6**H), 7.50–7.52 (2 H, m, C**6'**H), 7.66 (1 H, tt, $^3J_{HH}$ 7.3, $^4J_{HH}$ 1.3, C**7'**H), 8.08-8.09 (2 H, m, C**5'**H), 8.13–8.15 (2 H, m, C**5**H); $^{19}$F NMR (376 MHz, $CDCl_3$) δ −102.6 (s, $CF_2$, C**2**F), −100.6 (tt, $^3J_{HF}$ 8.2, $^4J_{HF}$ 5.3, C**7**F); $^{13}$C{$^1$H} NMR (176 MHz, $CDCl_3$) δ 112.8 (t, $^1J_{CF}$ 265.8, C**2**), 116.5 (d, $^2J_{CF}$ 22.1, C**6**), 128.2 (d, *J* 2.9, C**4**), 129.1 (s, C**6'**), 130.4 (t, $^4J_{CF}$ 2.6, C**5'**), 131.7 (C**4'**), 133.4 (dt, $^3J_{CF}$ 9.9, $^4J_{CF}$ 2.8, C**5**), 135.3 (C**7**), 167.0 (d, $^1J_{CF}$ 259.2, C**2**), 186.0 (t, $^2J_{CF}$ 27.1, C**1**), 187.5 (t, $^2J_{CF}$ 26.8 C**3**); *m/z* (EI$^+$) 278.1 ([M]$^+$, 2%), 123.2 ([$C_7H_4FO$]$^+$, 100%), 105.2 ([$C_7H_5O$]$^+$, 100%), 95.1 ([C6H4F]$^+$, 58%), 77.1 ([$C_6H_5$]$^+$, 90%), 51.1 ([$C_3H_4$]$^+$, 26%). HRMS (ASAP) *m/z* calculated for [M+H]$^+$ $C_{15}H_{10}F_3O_2$ 279.0633; found 279.0642. IR (neat cm$^{-1}$) 3080, 1693, 1597, 1450, 1241, 1108, 885, 683, 572.

### 1.5.3 2,2-Difluoro-1-(4-nitrophenyl)-3-phenylpropane-1,3-dione (3f)

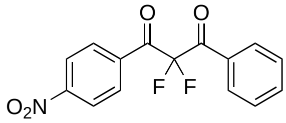

Prepared according to **GP2**, 1-(4-nitrophenyl)-3-phenylpropane-1,3-dione (0.535 g, 1.99 mmol) in acetonitrile (60 mL) after column chromatography with hexane and dichloromethane (1:1) as the eluent yielded *2,2-difluoro-1-(4-nitrophenyl)-3-phenylpropane-1,3-dione* (0.304 g, 50%) as a pale yellow solid; Mp 54–56 °C. $^{1}$H NMR (700 MHz, $CDCl_3$) δ 7.52–7.58 (2 H, m), 7.71 (1 H, tt, $^{3}J_{HH}$ 7.5, $^{4}J_{HH}$ 1.2), 8.09–8.12 (2 H, m), 8.22–8.25 (2 H, m), 8.33–8.35 (2 H, m); $^{19}$F NMR (376 MHz, $CDCl_3$) δ −102.7 (p, $^{5}J_{HF}$ 0.8); $^{13}$C{$^{1}$H} NMR (176 MHz, $CDCl_3$) δ 112.5 (t, $^{1}J_{CF}$ 266.9, C**2**), 124.1 (C**6)**, 129.3 (C**6')**, 130.5 (t, $^{5}J_{CF}$ 2.7, C**5'**), 131.3 (C**4'**), 131.5 (t, $^{5}J_{CF}$ 2.8, C**4**), 135.7 (C**7'**), 136.3 (C**4**), 151.2 (C**7**), 186.6 (t, $^{2}J_{CF}$ 27.7 C**1**), 187.4 (t, $^{2}J_{CF}$ 27.1, C**3**); *m/z* (EI$^{+}$) 150.1 ($[C_7H_4NO_3]^+$, 32%), 105.2 ($[C_7H_5O]^+$, 100%), 77.1 ($[C_6H_5]^+$, 54%), 51.1 ($[C_3H_4]^+$, 10%). HRMS (ASAP) *m/z* calculated for $[M+H]^+$ $C_{15}H_{10}F_2NO_4$ 306.0567; found 306.0578. Crystals suitable for X-ray diffraction were grown by slow evaporation from hexane—see section3.4. IR (neat cm$^{-1}$) 3111, 1698, 1525, 1290, 1101, 954, 841, 711, 522.

### 1.5.5 2,2-Difluoro-1,3-bis(4-chlorophenyl)propane-1,3-dione (3g)

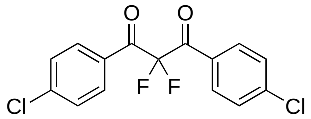

Prepared according to **GP2**, 1,3-bis(4-chlorophenyl)-propane-1,3-dione (0.590 g, 2.01 mmol) in acetonitrile (60 mL) after column chromatography with hexane and dichloromethane (1:1) as the eluent yielded *1,3-bis(4-chlorophenyl)-2,2-difluoro-propane-1,3-dione* (0.475 g, 72%) as a white solid; Mp 93–94 °C. $^{1}$H NMR (700 MHz, $CDCl_3$) δ 7.48–7.50 (4 H, m, C**5**H), 8.02 (4 H, d, $^{3}J_{HH}$ 8.4, C**4**H); $^{19}$F NMR (376 MHz, $CDCl_3$) δ −102.8 (s); $^{13}$C{$^{1}$H} NMR (176 MHz, $CDCl_3$) δ 112.7 (t, $^{1}J_{CF}$ 266.3, C**2**), 129.6 (C**5**), 129.9 (t, $^{3}J_{CF}$ 1.5, C**3**), 131.8 (t, $^{4}J_{CF}$ 2.8, C**4**), 142.2 (C**6**), 186.4 (t, $^{2}J_{CF}$ 27.1, C**1**); *m/z* (EI)$^{+}$ 328.0 ($[M]^+$, 1%), 139.1 ($[C_7H_4{}^{35}ClO]^+$, 100%), 111.0 ($[C_6H_4{}^{35}Cl]^+$, 41%), 50.0 ($[C_4H_2]^+$, 4%). HRMS (ASAP) *m/z* calculated for $[M+H]^+$ $C_{15}H_9{}^{35}Cl_2F_2O_2$ 328.9948; found 328.9937. IR (neat cm$^{-1}$) 1698, 1588, 1488, 1405, 1251, 1139, 1013, 884, 773, 542.

### 1.5.6 2,2-Difluoro-1,3-bis(4-fluorophenyl)propane-1,3-dione (3h)

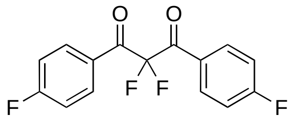

Prepared according to **GP2**, 1,3-bis(4-fluorophenyl)-propane-1,3-dione (0.522 g, 2.01 mmol) in acetonitrile (60 mL) after recrystallisation from hexane and chloroform yielded *2,2-difluoro-1,3-bis(4-fluorophenyl)propane-1,3-dione* (0.451 g, 76%) as a pale yellow solid; Mp 79–81 °C. $^{1}$H NMR (500 MHz, $CDCl_3$) δ 7.16–7.20 (4 H, m, C**5**H), 8.12–8.16 (4 H, m, C**4**H); $^{19}$F NMR (376 MHz, $CDCl_3$) δ −102.6 (s, C**2**$F_2$), −100.3 (tt, $^{3}J_{HF}$ 8.2, $^{4}J_{HF}$ 5.3, C**6**F); $^{13}$C{$^{1}$H} NMR (126 MHz, $CDCl_3$) δ 112.9 (t, $^{1}J_{CF}$ 265.9, C**2**), 116.5 (d, $^{2}J_{CF}$ 22.2, C**5**), 128.1 (dt, $^{3}J_{CF}$ 3.1, $^{4}J_{CF}$ 1.6, C**3**), 133.5 (dt, $^{4}J_{CF}$ 9.9, $^{5}J_{CF}$ 2.9, C**4**), 167.0 (d, $^{1}J_{CF}$ 259.4, C**6**), 186.0 (t, $^{2}J_{CF}$ 27.0, C**1**); *m/z* (EI)$^+$ 296 ([M]$^+$, 1%), 123.1 ([$C_7H_4FO$]$^+$, 100%), 95.1 ([$C_6H_4F$]$^+$, 78%), 50.0 ([$C_4H_2$]$^+$, 3%). HRMS (ASAP) *m/z* calculated for [M+H]$^+$ $C_{15}H_9F_4O_2$ 297.0539; found 297.0537. IR (neat cm$^{-1}$) 1695, 1596, 1504, 1413, 1235, 1134, 867, 780, 600. Data consistent with those previously reported in the literature.[12]

### 1.5.7 2,2-Difluoro-1,3-bis(4-nitrophenyl)-propane-1,3-dione (3i)

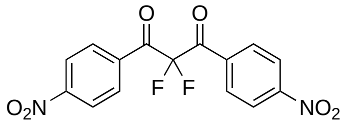

Prepared according to **GP2**, 1,3-bis(4-nitrophenyl)-propane-1,3-dione (0.630 g, 2.00 mmol) in acetonitrile (60 mL) after recrystallisation from hexane and chloroform yielded *1,3-bis(4-nitrophenyl)-2,2-difluoropropane-1,3-dione* (0.542 g, 77%) as a pale yellow solid; Mp 117–119 °C. $^{1}$H NMR (700 MHz, $CDCl_3$) δ 8.25–8.28 (4 H, m, C**4**H), 8.37–8.40 (4 H, m, C**5**H); $^{19}$F NMR (376 MHz, $CDCl_3$) δ −102.9 (s); $^{13}$C{$^{1}$H} NMR (176 MHz, $CDCl_3$) δ 112.2 (t, $^{1}J_{CF}$ 267.7, C**2**), 124.3 (C**5**), 131.5 (t, $^{4}J_{CF}$ 2.9, C**4**), 135.8 (C**6**), 151.5 (C**2**), 186.3 (t, $^{2}J_{CF}$ 27.9, C**1**). *m/z* (EI)$^+$ 150.1 ([$C_7H_4NO_3$]$^+$, 100%), 76.1 ([$C_6H_4$]$^+$, 22%). HRMS (ASAP) *m/z* calculated for [M+H]$^+$ $C_{15}H_9F_2N_2O_6$ 351.0430; found 351.0429. Crystals suitable for X-ray diffraction were grown by slow evaporation from hexane—see section 3.5. IR (neat cm$^{-1}$) 3112, 1729, 1520, 1349, 1126, 955, 827, 715, 562.

## 1.6 Synthesis of 4b–h

### General procedure 3 (GP3)– synthesis of compounds 4b–h

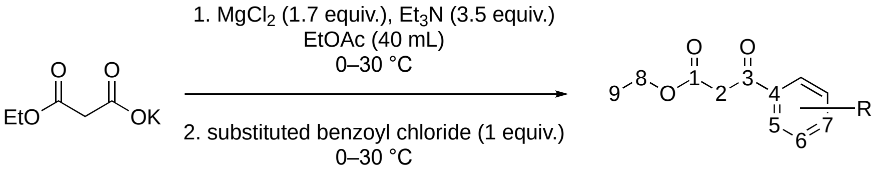


Ethyl potassium malonate (2.383 g, 14 mmol) in ethyl acetate (40 mL) was cooled to 0 °C. Triethylamine (4.9 mL, 35 mmol) and magnesium chloride (1.619 g, 17 mmol) were added, and the resulting slurry was stirred at 30 °C for 6 h before the mixture was cooled to 0 °C and the corresponding benzoyl chloride (10 mmol) was added dropwise over 5 min. The mixture was stirred overnight at rt before being cooled to 0 °C and quenched with 13% hydrochloric acid (30 mL). The aqueous phase was removed and extracted with toluene (20 mL), then the combined organic phases were washed with 13% hydrochloric acid (2 × 10 mL) and water (2 × 10 mL), dried over magnesium sulphate and the solvents were removed under reduced pressure. The crude product was filtered through a silica plug with chloroform as the eluent to give the desired product.

### 1.6.1 Ethyl (4-methoxy)benzoylacetate (4b)

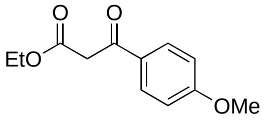


4-(Methoxy)benzoyl chloride (1.354 mL, 10.00 mmol) gave *ethyl (4-methoxy)benzoylacetate* (1.658 g, 75%) as a pale orange oil containing a mixture of keto- and two enol tautomers. *Ketone-tautomer* (75%) $^{1}$H NMR (700 MHz, $CDCl_3$) δ 1.26 (3 H, t, $^{3}J_{HH}$ 7.1, C**9**H), 3.88 (3 H, s, C**10**H), 3.94 (2 H, s, C**2**H), 4.21 (2 H, q, $^{3}J_{HH}$ 7.1, C**8**H), 6.93–6.96 (2 H, m, C**6**H), 7.91–7.94 (2 H, m, C**5**H); $^{13}$C{$^{1}$H} NMR (176 MHz, $CDCl_3$) δ 14.2 (C**9**), 46.0 (C**2**), 55.7 (C**10**), 61.6 (C**8**), 114.1 (C**6**), 129.3 (C**4**), 131.1 (C**5**), 164.1 (C**7**), 167.9 (C**1**), 191.1 (C**3**); *enol tautomer 1* (17%) $^{1}$H NMR (700 MHz, $CDCl_3$) δ 5.58 (1 H, s, C**2**H), 3.85 (3H, s, C**10**H); *enol tautomer 2* (8%) $^{1}$H NMR (700 MHz, $CDCl_3$) δ 6.06 (1 H, s, C**2**H), 3.86 (3H, s, C**10**H); *m/z* (EI$^{+}$) 222.1 ([M]$^{+}$, 7%), 150.1 ([$C_9H_{10}O_2$]$^{+}$, 14%), 135.1 ([$C_8H_7O_2$]$^{+}$, 100%), 107.1 ([$C_7H_7O$]$^{+}$, 15%), 77.1 (([$C_6H_5$]$^{+}$, 18%). HRMS (ASAP) *m/z* calculated for [M+H]$^{+}$ $C_{12}H_{15}O_4$ 223.0970, found 223.0968. IR (neat, cm$^{-1}$) 2982, 1675, 1599, 1511, 1422, 1257, 1170, 1024, 843, 566. Data consistent with those previously reported in the literature.[13]

### 1.6.2 Ethyl (4-chloro)benzoylacetate (4c)

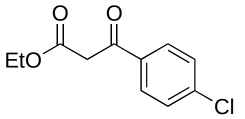

4-(Chloro)benzoyl chloride (1.282 mL, 10.00 mmol) gave *ethyl (4-chloro)benzoylacetate* (2.053 g, 91%) as a low-melting yellow solid as a mixture of keto- and two enol tautomers. *Ketone tautomer* (73%) $^1$H NMR (700 MHz, $CDCl_3$) δ 1.25 (3 H, t, $^3J_{HH}$ 7.1, C**9**H), 3.95 (2 H, s, C**2**H), 4.20 (2 H, q, $^3J_{HH}$ 7.2, C**8**H), 7.43–7.46 (2 H, m, C**6**H), 7.87–7.90 (2 H, m, C**5**H); $^{13}$C{$^1$H} NMR (176 MHz, $CDCl_3$) δ 14.19 (s, C**9**), 46.06 (s, C**2**), 61.74 (s, C**8**), 129.25 (s, C**6**), 130.06 (s, C**5**), 140.42 (s, C**7**), 167.32 (s, C**1**), 191.42 (s, C**3**); *enol tautomer 1* (20%) $^1$H NMR (700 MHz, $CDCl_3$) δ 5.63 (1 H, s, C**2**H), 12.57 (1H, s, C**2**OH); *enol tautomer 2* (7%) $^1$H NMR (700 MHz, $CDCl_3$) δ 6.04 (1 H, s, C**2**H), 13.48 (1H, s, C**2**OH); *m/z* (EI$^+$) 226.0, ([M]$^+$, 5%), 154.1 ([$C_8H_7{}^{35}ClO$]$^+$, 9%), 139.1 ([$C_7H_4{}^{35}ClO$]$^+$, 100%), 111.0 ([$C_6H_4{}^{35}Cl$]$^+$, 29%). HRMS (ASAP) *m/z* calculated for [M+H]$^+$ = $C_{11}H_{12}{}^{35}ClO_3$ 227.0475, found 227.0471. Crystals suitable for X-ray diffraction were grown by slow evaporation from hexane—see section 3.6. IR (neat, cm$^{-1}$) 2980, 2160, 1722, 1624, 1426, 1258, 1177, 1073, 796. Data consistent with those previously reported in the literature.[13a, 14]

### 1.6.3 Ethyl (4-trifluoromethyl)benzoylacetate (4d)

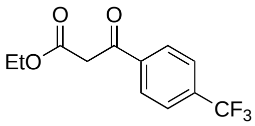

4-(Trifluoromethyl)benzoyl chloride (1.486 mL, 10.00 mmol) gave a mixture of keto- and enol isomers of *ethyl (4- trifluoromethyl)benzoylacetate* (2.463 g, 95%) as a pale orange oil. *Ketone tautomer* (60%) $^1$H NMR (700 MHz, $CDCl_3$) δ 1.26 (3 H, t, $^3J_{HH}$ 7.1, C**9**H), 4.01 (2 H, s, C**2**H), 4.22 (2 H, q, $^3J_{HH}$ 7.1, C**8**H), 7.76 (2 H, d, $^3J_{HH}$ 8.2, C**6**H), 8.04–8.07 (2 H, m, C**5**H); $^{19}$F NMR (376 MHz, $CDCl_3$) δ −63.2 (s, C**10**F3), $^{13}$C{$^1$H} NMR (176 MHz, $CDCl_3$) δ 14.2 (C**9**), 46.3 (C**2**), 61.9 (C**8**), 122.8 (q, $^1J_{CF}$ 273.0, C**10**), 126.0 (q, $^4J_{CF}$ 3.7, C**6**), 129.0 (C**5**), 135.1 (q, $^2J_{CF}$ 32.8, C**7**), 138.8 (C**4**), 167.1 (C**1**), 191.7 (C**3**); *enol tautomer* (40%) $^1$H NMR (700 MHz, $CDCl_3$) δ 1.35 (3 H, t, $^3J_{HH}$ 7.1, C**9**H), 4.29 (2 H, q, $^3J_{HH}$ 7.1, C**8**H),5.71 (1 H, s, C**2**H), 7.68 (2 H, d, $^3J_{HH}$ 8.3, C**6**H), 7.87–7.89 (2 H, m, C**5**H), 12.56 (1 H, s, C**3**OH); $^{19}$F NMR (376 MHz, $CDCl_3$) δ −63.0 (s, C**10**$F_3$ $^{13}$C{$^1$H} NMR (176 MHz, $CDCl_3$) δ 14.4 (C**9**), 60.8 (C**8**), 89.2 (C**2**), 124.7 (q, $^1J_{CF}$ 272.9, C**10**), 125.7

(q, $^4J_{CF}$ 3.8, C**6**), 126.5 (C**5**), 132.9 (q, $^2J_{CF}$ 32.6, C**7**), 137.0 (s, C**4**), 169.6 (C**1**), 173.0 (C**3**); *m/z* (EI$^+$) 260.1, ([M]$^+$, 6%), 173.1 ([$C_8H_4F_3O$]$^+$, 100%), 145.1 ([$C_7H_4F_3$]$^+$, 39%), 69.0 ([$CF_3$]$^+$, 3%), 45.0 ([$C_2H_5O$]$^+$, 3%). HRMS (ASAP) *m/z* calculated for [M+H]$^+$ = $C_{12}H_{12}F_3O$ 261.0739, found 261.0743. IR (neat, cm$^{-1}$) 2980, 2160, 1722, 1624, 1426, 1258, 1177, 1073, 796. Data consistent with those previously reported in the literature.[13a]

### 1.6.4 Ethyl (4-nitro)benzoylacetate (4e)

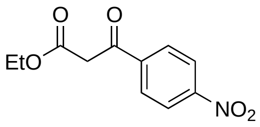


4-(Nitro)benzoyl chloride (1.856 g, 10.00 mmol) was dissolved in ethyl acetate (5 mL) and gave a mixture of keto- and enol isomers of *ethyl (4-nitro)benzoylacetate* (2.336 g, 98%) as a peach coloured solid; Mp 62–63 °C [lit. Mp. 63–67 °C[14]; 68-69 °C[15]]. *Enol tautomer* (74%) $^1$H NMR (700 MHz, $CDCl_3$) δ 1.35 (3 H, t, $^3J_{HH}$ 7.2, C**9**H), 4.30 (2 H, q, $^3J_{HH}$ 7.1, C**8**H), 5.76 (1 H, s, C**2**H), 7.92–7.95 (2 H, m, C**5**H), 8.26–8.29 (2 H, m, C**6**H), 12.57 (1 H, s, C**2**OH); $^{13}$C{$^1$H} NMR (176 MHz, $CDCl_3$) δ 14.4 (C**9**), 61.0 (C**8**), 90.4 (C**2**), 123.9 (C**6**), 127.1 (C**5**), 139.5 (C**4**), 149.4 (C**7**), 168.4 (C**1**), 172.8 (C**3**); *ketone tautomer* (26%) $^1$H NMR (700 MHz, $CDCl_3$) δ 1.26 (3 H, t, $^3J_{HH}$ 7.1, C**9**H), 4.03 (2 H, s, C**2**H), 4.22 (2 H, q, $^3J_{HH}$ 7.1, C**9**H), 8.10–8.13 (2 H, m, C**5**H), (8.32–8.35 (2 H, m, C**6**H), $^{13}$C{$^1$H} NMR (176 MHz, $CDCl_3$) δ 14.2 (C**9**), 46.4 (C**2**), 62.0 (C**8**), 124.1 (C**6**), 129.7 (C**5**), 140.5 (C**4**), 150.8 (C**7**), 166.8 (C**1**), 191.2 (C**3**); *m/z* (EI$^+$) 165.0 ([$C_8H_7NO_3$]$^+$, 20%), 150.1 ([$C_7H_4NO_3$]$^+$, 100%). HRMS (ASAP) *m/z* calculated for [M+H]$^+$ = $C_{11}H_{11}NO_5$ 238.0715, found 238.0715. IR (neat, cm$^{-1}$) 3114, 2910, 2160, 1619, 1519, 1428, 1339, 1215, 1032, 797. Data consistent with those previously reported in the literature.[14-15]

### 1.6.5 Ethyl (4-cyano)benzoylacetate (4f)

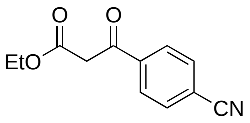


4-(Cyano)benzoyl chloride (1.656 g, 10.00 mmol) was dissolved in ethyl acetate (5 mL) and gave a mixture of keto- and enol isomers of *ethyl (4-cyano)benzoylacetate* (2.060 g, 95%) as a white solid after recrystallisation from ethanol; Mp 60–62 °C [lit.

Mp. 62–63 °C;[16]]. *Enol tautomer* (88%) $^1$H NMR (400 MHz, $CDCl_3$) δ 1.34 (3 H, t, $^3J_{HH}$ 7.1, C**9**H), 4.30 (2 H, q, $^3J_{HH}$ 7.1, C**8**H), 5.72 (1 H, s, C**2**H), 7.71 (2 H, d, $^3J_{HH}$ 7.6, C**6**H), 7.86 (2 H, d, $^3J_{HH}$ 7.6, C**5**H), 12.57 (1 H, s, C**2**OH); $^{13}$C{$^1$H} NMR (101 MHz, $CDCl_3$) δ 14.4 (C**9**), 60.9 (C**8**), 89.9 (C**2**), 123.9 (C**6**), 137.7 (C**4**), 118.4 (C**10**), 126.7 (C**5**), 132.5 (C**6**), 149.4 (C**7**), 168.7 (C**1**), 172.8 (C**3**); *ketone tautomer* (12%) $^1$H NMR (400 MHz, $CDCl_3$) δ 1.25 (3 H, t, $^3J_{HH}$ 7.0, C**9**H), 4.00 (2 H, s, C**2**H), 4.22 (2 H, q, $^3J_{HH}$ 7.0, C**8**H), 7.79 (2 H, d, $^3J_{HH}$ 7.7, C**6**H), 8.04 (2 H, d, $^3J_{HH}$ 7.7, C**5**H); $^{13}$C{$^1$H} NMR (101 MHz, $CDCl_3$) δ 14.2 (C**9**), 46.2 (C**2**), 62.0 (C**8**), 117.1 (C**7**), 117.9 (C**10**), 129.1 (C**5**), 132.8 (C**6**), 139.0 (C**4**), 166.9 (C**1**), 191.4 (C**3**); *m/z* ($EI^+$) 145.1 ($[C_9H_7NO]^+$, 34%), 130.1 ($[C_8H_4NO]^+$, 100%), 102.1 ($[C_7H_4N]^+$, 70%), 75.1 ($[C_3H_7O_2]^+$, 16%), 63.1 ($[C_2H_5O_2]^+$, 2%), 51.1 ($[C_4H_3]^+$, 9%), 43.1 ($[C_2H_3O]^+$, 19%). HRMS (ASAP) *m/z* calculated for $[M+H]^+$ = $C_{12}H_{12}NO_3$ 218.0817, found 218.0836. IR (neat, $cm^{-1}$) 2999, 2233, 2159, 1977, 1739, 1621, 1422, 1356, 1253, 1192, 1029, 800, 715, 543, 433. Data are consistent with those previously reported in the literature.[17]

### 1.6.6 Ethyl (3-nitro)benzoylacetate (4g)

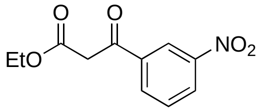


3-(Nitro)benzoyl chloride (1.856 g, 10.00 mmol) was dissolved in ethyl acetate (5 mL) and reacted according to the general procedure to yield a mixture of keto- and enol isomers of *ethyl (3-nitro)benzoylacetate* (1.777 g, 75%) as a peach coloured solid; Mp 70–71 °C [lit. Mp. 80–82 °C[15]; 85-87 °C]. *Ketone tautomer* (58%) $^1$H NMR (700 MHz, $CDCl_3$) δ 1.27 (3 H, t, $^3J_{HH}$ 7.1, C1**1**H), 4.05 (2 H, s, C**2**H), 4.23 (2 H, q, $^3J_{HH}$ 7.1, C**10**H), 7.71 (1 H, t, $^3J_{HH}$ 7.9, C**8**H), 8.29 (1 H, dt, $^3J_{HH}$ 7.9, $^4J_{HH}$ 1.2, C**9**H), 8.46 (1 H, ddd, $^3J_{HH}$ 8.2, $^4J_{HH}$ 2.1, $^4J_{HH}$ 1.2, C**7**H), 8.77 (1 H, t, $^4J_{HH}$ 2.1, C**5**H); $^{13}$C{$^1$H} NMR (176 MHz, $CDCl_3$) δ 14.2 (C1**1**), 46.1 (C**2**), 62.0 (C**10**), 123.6 (C**5**), 128.1 (C**7**), 130.3 (C**8**), 134.1 (C**9**), 137.4 (C**4**), 148.7 (C**6**), 166.8 (C**1**), 190.6 (C**3**); *enol tautomer* (42%) $^1$H NMR (700 MHz, $CDCl_3$) δ 1.35 (3 H, t, $^3J_{HH}$ 7.1, C1**1**H), 4.30 (1 H, q, $^3J_{HH}$ 7.1, C**10**H), 5.77 (1 H, s, C**2**H), 7.62 (1 H, t, $^3J_{HH}$ 7.9, C**8**H), 8.10 (1 H, ddd, $^3J_{HH}$ 7.9, $^4J_{HH}$ 1.7, $^4J_{HH}$ 1.0, C**9**H), 8.31 (1 H, ddd, $^3J_{HH}$ 8.2, $^4J_{HH}$ 2.2, $^4J_{HH}$ 1.0, C**7**H), 8.62 (1 H, t, $^4J_{HH}$ 2.2, C**5**H), 12.62 (1 H, s, C**3**OH); $^{13}$C{$^1$H} NMR (176 MHz, $CDCl_3$) δ 14.4 (C1**1**), 61.0 (C**10**), 89.4 (C**2**), 121.2 (C**5**), 125.7 (C**7**), 129.8 (C**8**), 131.8 (C**9**), 135.4 (C4),128.1 (C**9**), 148.6 (C**6**), 168.4 (C**1**), 172.9 (C**3**); *m/z* ($ESI^+$) 238.3 ($[C_{11}H_{11}NO_5]^+$, 100%), 115.1 ($[C_7H_5NO_3]^+$, 29%), *m/z* ($EI^+$) 165.0

([$C_8H_7NO_3$]$^+$, 17%), 150.1 ([$C_7H_4NO_3$]$^+$, 100%). HRMS (ASAP) *m/z* calculated for [M+H]$^+$ = $C_{11}H_{11}NO_5$ 238.0715, found 238.0722. Crystals suitable for X-ray diffraction were grown by slow evaporation from hexane—see section 3.7. Data consistent with those previously reported in the literature.[13b]

### 1.6.7 Ethyl 3-oxo-3-(pyridin-4-yl)propanoate (4h)

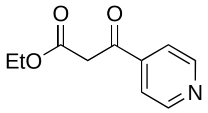

Ethyl isonicotinate (1.498 mL, 10 mmol) was dissolved in ethyl acetate (30 mL) and cooled to −50 °C before lithium hexamethyldisilizane (1 M in tetrahydrofuran, 30 mL, 30 mmol) was added and the mixture was stirred for 20 min. The reaction was quenched with acetic acid (50 mmol), basified with saturated sodium bicarbonate solution and extracted with ethyl acetate (2×100 mL). The organic phases were combined, washed with water (20 mL), saturated sodium chloride solution (20 mL) before drying over magnesium sulphate and the solvents removed under reduced pressure. Recrystallisation from ethanol gave a mixture of keto- and enol isomers of the desired *ethyl 3-oxo-(pyridine-4-yl)propanoate* (1.377 g, 71%) as a white solid; Mp 52–54 °C [lit. Mp. 57.5-58 °C[18]]. *Ketone tautomer* (42%) $^1$H NMR (600 MHz, $CDCl_3$) δ 1.25 (3 H, t, $^3J_{HH}$ 7.1, C**8**H), 3.98 (2 H, s, C**2**H), 4.21 (2 H, q, $^3J_{HH}$ 7.1, C**7**H), 7.71 (2 H, dt, $^3J_{HH}$ 4.3, $^4J_{HH}$ 1.3C**5**H), 8.83 (2 H, dt, $^3J_{HH}$ 4.4, $^4J_{HH}$ 1.3, C**6**H); $^{13}$C{$^1$H} NMR (151 MHz, $CDCl_3$) δ 14.2 (C**8**), 46.1 (C**2**), 62.0 (C**7**), 121.4 (C**5**), 141.9 (C**4**), 151.3 (C**6**), 166.8 (C**1**), 192.2 (C**3**); *enol tautomer* (58%) $^1$H NMR (600 MHz, $CDCl_3$) δ 1.34 (3 H, t, $^3J_{HH}$ 7.1, C**8**H), 4.28 (2 H, q, $^3J_{HH}$ 7.2, C**7**H), 5.76 (1 H, s, C**2**H), 7.60 (2 H, dt, $^3J_{HH}$ 4.5, $^4J_{HH}$1.3, C**5**H), 8.70 (2 H, dt, $^3J_{HH}$ 4.4, $^4J_{HH}$ 1.3, C**6**H), 12.43 (1 H, s, COH);$^{13}$C{$^1$H} NMR (151 MHz, $CDCl_3$) δ 14.4 (C**8**), 61.0 (C**7**), 90.1 (C**2**), 119.8 (C**5**), 140.9 (C**4**), 150.6 (C**6**), 168.4 (C**3**), 172.8 (C**1**); *m/z* (ESI$^+$) 194.1 ([$C_{10}H_{11}NO_3$]$^+$, 100%), 148.1 ([$C_8H_6NO_2$]$^+$, 69%), 120.1 (([$C_7H_6NO$]$^+$, 3%), 106.0 ([$C_6H_4NO$]$^+$, 1%). HRMS (ASAP) (*m/z*) calculated for [M+H]$^+$ $C_{10}H_{12}NO_3$ 194.0817; found 194.0810. Data consistent with those previously reported in the literature.[19]

## 1.7 Screening vonditions for the base-mediated direct fluorination of ethyl benzoylacetate

Ethyl benzoylacetate (0.346 mL, 2.0 mmol) and the mediating agent were added to a SIMAX glass bottle and dissolved in acetonitrile (20 mL). The reaction vessel was cooled to 0 °C, stirred rapidly and purged with nitrogen for 10 minutes before fluorine gas, as a 10% mixture in nitrogen (v/v) was passed through the reaction mixture at a prescribed flow rate, (15 mL min$^{-1}$) that was controlled by a mass flow controller. After purging with nitrogen for 10 minutes, the reaction vessel was disconnected and the solvent removed under reduced pressure. The organic products were washed through a silica plug with chloroform (100 mL) and concentrated under reduced pressure to give the crude product which was analysed by NMR spectroscopy.

**Table S3:** Screening conditions for the base mediated fluorination of ethyl benzoylacetate

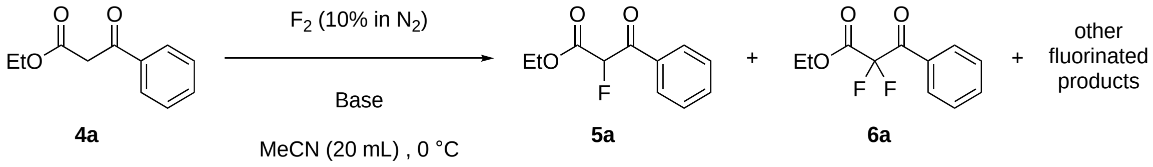


| Entry | Base | Equiv of base | Equiv. of F2 | NMR yield/% | |
|---|---|---|---|---|---|
| | | | | **5a** | **6a** |
| 1 | None | - | 2.3 | 8 | 2 |
| 2 | DABCO | 1.1 | 2.3 | 16 | 10 |
| 3 | DABCO[a] | 1.1 | 2.3 | 1 | 1 |
| 4 | DABCO[b] | 1.1 | 2.3 | 0 | 0 |
| 5 | DABCO | 1.5 | 2.3 | 0 | 3 |
| 6 | DABCO | 1.5 | 4.0 | 0 | 1 |
| 7 | DBU | 0.5 | 2.3 | 12 | 6 |
| 8 | DBU | 1.5 | 2.3 | 18 | 3 |
| 9 | Et3N | 1.1 | 2.3 | 11 | 1 |
| 10 | 3-Quinuclidinol[a] | 1.1 | 2.3 | 0 | 1 |
| 11 | Quinuclidine | 1.1 | 2.3 | 0 | 42 |
| 12 | Quinuclidine | 1.1 | 3.5 | 6 | 41 |
| 13 | Quinuclidine | 1.1 | 3.0[c] | 6 | 51 |
| 14 | Quinuclidine | 1.5 | 3.0[c] | 0 | 73 |
| 15 | Quinuclidine | 1.5 | 3.0[c] | 0 | 85[d] |

[a] 40 mL MeCN used; [b] 40 mL MeCN, 4.0 mmol, **4a**, 2.3 equiv. F2 and 1.1 equiv. DABCO used; [c] 30 mL min$^{-1}$ flow rate of 10% *v/v* F2 in N2 used; [d] Isolated yield after 20 mL column

## 1.8 Synthesis of 5a–g

### General procedure 4 (GP4) — synthesis of compounds 5a–g

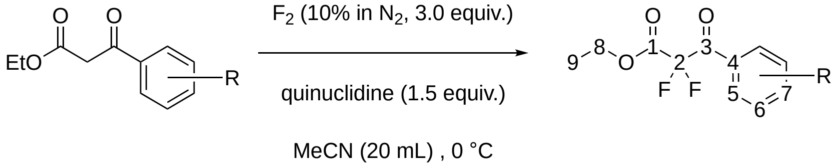


Quinuclidine (0.334 g, 3.00 mmol, 1.5 equiv) and the relevant ethyl benzoylacetate (2.00 mmol, 1.0 equiv) were added to a SIMAX glass bottle and dissolved in acetonitrile (20 mL). The reaction vessel was cooled to 0 °C, stirred rapidly and purged with nitrogen for 10 minutes before fluorine, as a 10% mixture in nitrogen (v/v), was passed through the reaction mixture at a prescribed flow rate, (6.00 mmol, 3.0 equiv, 30 mL $min^{-1}$) that was controlled by a mass flow controller for 49 min. After purging with nitrogen for 10 min., the reaction vessel was disconnected, and the solvent was removed under reduced pressure. The crude residue was filtered through a silica plug with chloroform as the eluent to give the desired product.

### 1.8.1 Ethyl 2,2-difluoro-3-oxo-3-phenylpropanoate (5a)

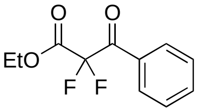

Following **GP4**, ethyl benzoylacetate (0.35 mL, 2.02 mmol) afforded *ethyl 2,2-difluoro-3-oxo-3-phenylpropanoate* (0.393 g, 85%) as a pale yellow oil; $^{1}$H NMR (700 MHz, $CDCl_3$) δ 1.32 (3 H, t, $^{3}J_{HH}$ 7.1, C**9**H), 4.39 (2 H, q, $^{3}J_{HH}$ 7.1, C**8**H), 7.51– 7.54 (2 H, m, C**6**H), 7.66–7.69 (1 H, m, C**7**H), 8.08 (2 H, ddt, $^{3}J_{HH}$ 7.8, $^{4}J_{HH}$ 2.2, $^{5}J_{HH}$ 1.1, C**5**H); $^{19}$F NMR (376 MHz, $CDCl_3$) δ −107.6 (s); $^{13}$C{$^{1}$H} NMR (176 MHz, $CDCl_3$) δ 14.0 (C**9**), 63.9 (C**8**), 109.9 (t, $^{1}J_{CF}$ 264.6, C**2**), 129.1 (C**4**), 130.1 (t, $^{4}J_{CF}$ 2.7, C**5**), 131.2 (t, $^{5}J_{CF}$ 2.0, C**6**), 135.2 (C**7**), 161.9 (t, $^{2}J_{CF}$ 30.5, C**1**), 185.6 (t, $^{2}J_{CF}$ 27.6, C**3**); *m/z* $(EI)^+$ 183.0 ($[C_9H_5F_2O_2]^+$, 2%), 155.1 ($[C_8H_5F_2O]^+$, 3%), 105.1 ($[C_7H_5O]^+$, 100%), 77.1 ($[C_6H_5]^+$, 76%), 51.0 ($[C_4H_3]^+$, 17%). HRMS (ASAP) *m/z* calculated for $[M+H]^+$ $C_{11}H_{11}F_2O_3$ 229.0676; found 229.0682. IR (neat, $cm^{-1}$) 2988, 1772, 1599, 1451, 1311, 1098, 922, 832, 685, 584. Data consistent with those previously reported in the literature.[20]

### 1.8.2 Ethyl 2,2-difluoro-3-(4-chlorophenyl)-3-oxo-propanoate (5c)

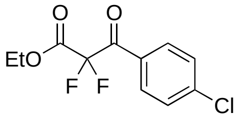


Following **GP4**, ethyl (4-chloro)benzoylacetate (0.453 g, 2.00 mmol,) afforded *ethyl 2,2-difluoro-3-(4-chlorophenyl)-3-oxopropanoate* (0.466 g, 89%) as a pale yellow oil; $^{1}$H NMR (600 MHz, $CDCl_3$) δ 1.33 (3H, t, $^{3}J_{HH}$ 7.1, C**9**H), 4.39 (2H, q, $^{3}J_{HH}$ 7.1, C**8**H), 7.49–7.52 (2H, m, C**6**H), 8.03 (2H, d, $^{3}J_{HH}$ 8.8, C**5**H); $^{19}$F NMR (376 MHz, $CDCl_3$) δ −107.6 (s, C**2**$F_2$); $^{13}$C{$^{1}$H} NMR (151 MHz, $CDCl_3$) δ 14.0 (s, C**9**), 64.0 (s, C**9**), 109.9 (t, $^{1}J_{CF}$ 264.8, C**2**), 129.5 (t, $^{3}J_{CF}$ 2.1, C**4**), 129.6 (s, C**6**), 134.5 (t, $^{4}J_{CF}$ 2.9, C**5**), 142.1 (s, C**7**), 161.7 (t, $^{2}J$CF 30.4, C**1**), 184.6 (t, $^{2}J_{CF}$ 28.0, C**3**); *m/z* $(EI)^+$ 217.0 ($[C_9H_4{}^{35}ClF_2O_2]^+$, 3%), 189.0 ($[C_8H_4ClF_2O]^+$, 3%), 139.1 ($[C_7H_4{}^{35}ClO]^+$, 100%), 111.0 ($[C_6H_4{}^{35}Cl]^+$, 83%), 85.0 ($[C_6H_{13}]^+$, 3%), 75.0 ($[C_3H_7O_2]^+$, 35%), 50.0 ($[C_4H_2]^+$, 9%). HRMS (ASAP) *m/z* calculated for $[C_{11}H_{10}{}^{35}ClF_2O_3]^+$ 263.0287; found 263.0287. IR (neat, $cm^{-1}$) 2988, 1773, 1702, 1589, 1491, 1373, 1406, 1310, 1254, 1158, 1088, 1014, 922, 847, 759, 549.

### 1.8.3 Ethyl 2,2-difluoro-3-(4-(trifluoromethyl)phenyl)-3-oxo-propanoate (5d)

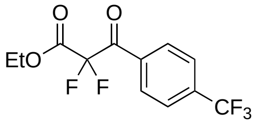


Following **GP4**, ethyl (4-trifluoromethyl)benzoylacetate (0.520 g, 2.00 mmol) afforded *ethyl 2,2-difluoro-3-(4-trifluoromethylphenyl)-3-oxopropanoate* (0.516 g, 87%) as a pale yellow oil; $^{1}$H NMR (700 MHz, $CDCl_3$) δ 1.34 (3H, t, $^{3}J_{HH}$ 7.1, C**9**H), 4.41 (2H, q, $^{3}J$HH 7.1, C**8**H), 7.79–7.81 (2H, m, C**6**H), 8.19–8.21 (2H, m, C**5**H); $^{19}$F NMR (376 MHz, $CDCl_3$) δ −107.9 (2F, s, C**2**$F_2$), −63.5 (3F, s, C**10**$F_3$); $^{13}$C{$^{1}$H} NMR (176 MHz, $CDCl_3$) δ 14.0 (s, C**9**), 64.2 (s, C**9**), 109.7 (t, $^{1}J_{CF}$ 264.9, C**2**), 123.4 (q, $^{1}J_{CF}$ 273.0, C**10**), 126.2 (q, $^{3}J$CF 3.8, C**6**), 130.5 (t, $^{4}J_{CF}$ 2.8, C**5**), 133.9 (s, C**4**), 136.3 (q, $^{2}J$CF 33.1, C**7**), 161.5 (t, $^{2}J$CF 30.3, C**1**), 185.1 (t, $^{2}J_{CF}$ 28.4, C**3**); *m/z* $(EI)^+$) 297.1 ($[C_{12}H_{10}F_5O_3]^+$, 34%), 269.0 ($[C_{10}H_6F_5O_3]^+$, 6%), 232.1 ($[C_{10}H_4F_4O_2$+, 3%), 173.0 ($[C_8H_4F_3O]^+$, 16%), 145 ($[C_77H_4F_3]^+$, 4%). HRMS (ASAP) *m/z* calculated for $[C_{12}H_{10}F_5O_3]^+$ 297.0539; found 297.0538. IR (neat, $cm^{-1}$) 1775, 1713, 1514, 1414, 1375, 1327, 1315, 1254, 1128, 1117, 1064, 1017, 925, 857, 832, 708, 595. Data consistent with those previously reported in the literature.[20a]

### 1.8.4 Ethyl 2,2-difluoro-3-(4-(nitrophenyl)-3-oxo-propanoate (5e)

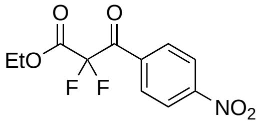


Following **GP4**, ethyl (4-nitro)benzoylacetate (0.474 g, 2.00 mmol) afforded *ethyl 2,2-difluoro-3-(4-nitrophenyl)-oxo-3-phenylpropanoate* (0.455 g, 83%) as a white solid; Mp 66–68 °C [lit. Mp. 80– 83 °C[11a]]. $^1$H NMR (700 MHz, $CDCl_3$) δ 1.34 (3 H, t, $^3J_{HH}$ 7.1, C**9**H), 4.41 (2 H, q, $^3J_{HH}$ 7.1, C**9**H), 8.24–8.27 (2 H, m, C**6**H), 8.35–8.38 (2 H, m, C**5**H); $^{19}$F NMR (376 MHz, $CDCl_3$) δ −107.8 (s); $^{13}$C{$^1$H} NMR (176 MHz, $CDCl_3$) δ 14.0 (C**9**), 64.4 (C**8**), 109.6 (t, $^1J_{CF}$ 265.1, C**2**), 124.2 (s, C**6**), 131.3 (t, $^4J_{CF}$ 2.9, C**5**), 135.6 (s, $^3J_{CF}$ 2.0, C**4**), 151.4 (s, C**7**), 161.2 (t, $^2J_{CF}$ 30.2, C**1**), 184.7 (t, $^2J_{CF}$ 28.8, C**3**); *m/z* (EI)$^+$ 228.0 ($[C_{11}H_{10}F_2O_3]^+$, 2%), 200.0 ($[C_8H_4F_2NO_3]^+$, 4%), 150.1 ($[C_7H_4NO_3]^+$, 100%), 76.1 ($[C_6H_4]^+$, 55%), 50.0 ($[C_4H_2]^+$, 13%). HRMS (ASAP) *m/z* calculated for $[M+H]^+$ $C_{11}H_{10}F_2NO_5$ 274.0527; found 274.0526. IR (neat, cm$^{-1}$) 3539, 3411, 2991, 1773, 1748, 1724, 1606, 1527, 1349, 1312, 1165, 1137, 1100, 1082, 1043, 1012, 1001, 926, 854, 827, 782, 733, 710, 671, 567. Crystals suitable for X-ray diffraction were grown by slow evaporation from hexane—see section 3.8. Data consistent with those previously reported in the literature.[11a]

### 1.8.5 Ethyl 2,2-difluoro-3-(4-(cyanophenyl)-3-oxo-propanoate (5f)

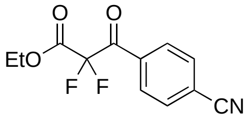


Following **GP4**, ethyl (4-cyano)benzoylacetate (0.4346 g, 2.00 mmol) afforded *ethyl 2,2-difluoro-3-(4-cyanophenyl)-3-oxopropanoate* (0.339 g, 67%) as a pale yellow oil; $^1$H NMR (400 MHz, $CDCl_3$) δ 1.33 (3H, t, $^3J_{HH}$ 7.1, C**9**H), 4.40 (2H, q, $^3J_{HH}$ 7.1, C**8**H), 7.80–7.86 (2H, m, C**6**H), 8.18 (2H, d, $^3J_{HH}$ 8.1, C**5**H); $^{19}$F NMR (376 MHz, $CDCl_3$) δ −107.8 (s); $^{13}$C{$^1$H} NMR (101 MHz, $CDCl_3$) δ 14.0 (s, C**9**), 64.3 (s, C**9**), 109.6 (t, $^1J_{CF}$ 265.0, C**2**), 117.5 (s, C**10**), 118.4 (s, C**7**), 130.4 (t, $^5J_{CF}$ 2.9, C**5**), 132.8 (s, C**6**), 134.1 (t, $^3J$CF 2.1, C**4**), 161.3 (t, $^2J$CF 30.2, C**1**), 184.8 (t, $^2J_{CF}$ 28.7, C**3**); *m/z* (ESI$^+$) 208.0 ($[C_{10}H_4FN0_2]^+$, 2%), 180.0 ($[C_9H_4F_2NO]^+$, 4%), 130.2 ($[C_8H_4NO]^+$, 100%), 102.1 ($[C_7H_4N]^+$, 76%), 75.1 ($[C_3H_7O2]^+$, 17%), 51.0 ($[C_4H_3]^+$, 9%). HRMS (ASAP) *m/z* calculated for $[C_{12}H_{10}F_2NO_3]^+$ 254.0629; found 254.0635. IR (neat, cm$^{-1}$) 2986, 2235, 1772, 1718, 1608, 1471, 1448, 1410, 1374, 1312, 1294, 1253, 1156, 1128, 1098, 1077, 1007, 924, 855, 765, 544. Data consistent with those previously reported in the literature.[20a]

### 1.8.6 Ethyl 2,2-difluoro-3-(3-(nitrophenyl)-3-oxo-propanoate (5g)

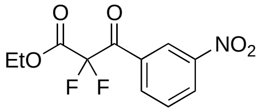


Following **GP4**, ethyl (3-nitro)benzoylacetate (0.474 g, 2.00 mmol) afforded *ethyl 2,2-difluoro-3-(3-nitrophenyl)-oxo-3-phenylpropanoate* (0.460 g, 84%) as a pale yellow oil. $^{1}$H NMR (700 MHz, $CDCl_3$) δ 1.35 (3 H, t, $^{3}J_{HH}$ 7.2, C1**1**H), 4.42 (2 H, q, $^{3}J_{HH}$ 7.1, C**10**H), 7.77–7.79 (1 H, m, C**9**H), 8.40 (1 H, ddq, $^{3}J_{HH}$ 8.1, $^{4}J_{HH}$ 1.9, $^{5}J_{HH}$ 0.8, C**8**H), 8.53 (1 H, ddd, $^{3}J_{HH}$ 8.1, $^{4}J_{HH}$ 2.3, $^{4}J_{HH}$ 1.1, C**7**H), 8.91 (1 H, ddd, $^{4}J_{HH}$ 2.3, $^{4}J_{HH}$ 1.6, $^{5}J_{HH}$ 0.8, C**5**H); $^{19}$F NMR (376 MHz, $CDCl_3$) δ −107.7 (s); $^{13}$C{$^{1}$H} NMR (176 MHz, $CDCl_3$) δ 14.0 (C1**1**), 64.4 (C**10**), 109.6 (t, $^{1}J_{CF}$ 265.2, C**2**), 125.0 (t, $^{4}J_{CF}$ 2.9, C**5**), 129.3 (C**7**), 130.5 (C**9**), 132.4 (t, $^{3}J_{CF}$ 2.2, C**4**), 135.4 (t, $^{5}J_{CF}$ 2.9, C**8**), 148.7 (C**6**), 161.2 (t, $^{2}J_{CF}$ 30.1, C**1**), 184.2 (t, $^{2}J_{CF}$ 28.8, C**3**); *m/z* (EI)$^{+}$ 228.0 ($[C_{11}H_{10}F_2O_3]^+$, 1%), 200.0 ($[C_8H_4F_2NO_3]^+$, 4%), 150.1 ($[C_7H_4NO_3]^+$, 100%), 76.1 ($[C_6H_4]^+$, 55%). HRMS (ASAP) *m/z* calculated for $[M+H]^+$ $C_{11}H_{10}F_2NO_5$ 274.0527; found 274.0525. IR (neat, cm$^{-1}$) 2990, 1773, 1714, 1615, 1534, 1350, 1316, 1250, 1163, 1126, 1078, 1003, 968, 949, 929, 857, 829, 719, 691, 585.

# 2 NMR Spectra

## 2.1 1-(Methylphenyl)-3-phenylpropane-1,3-dione (1b)

$^1$H NMR (700 MHz, $CDCl_3$)

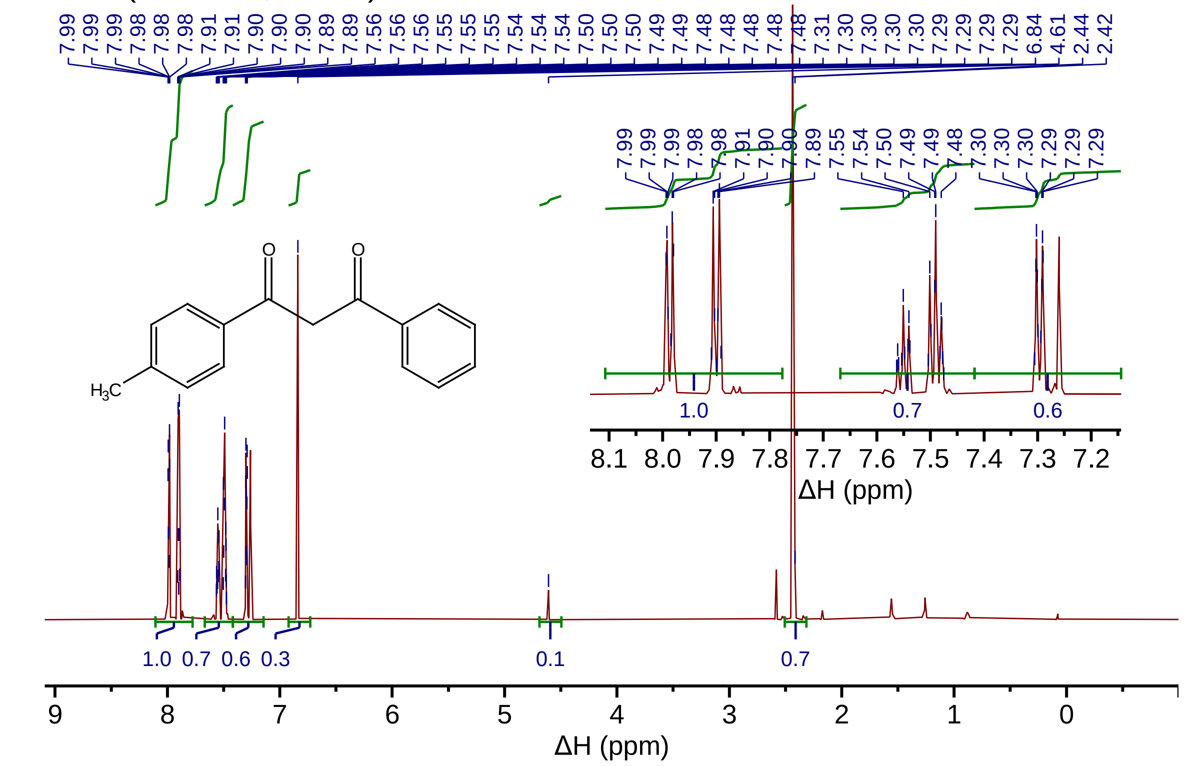


$^{13}C\{^1H\}$ NMR (176 MHz, $CDCl_3$)

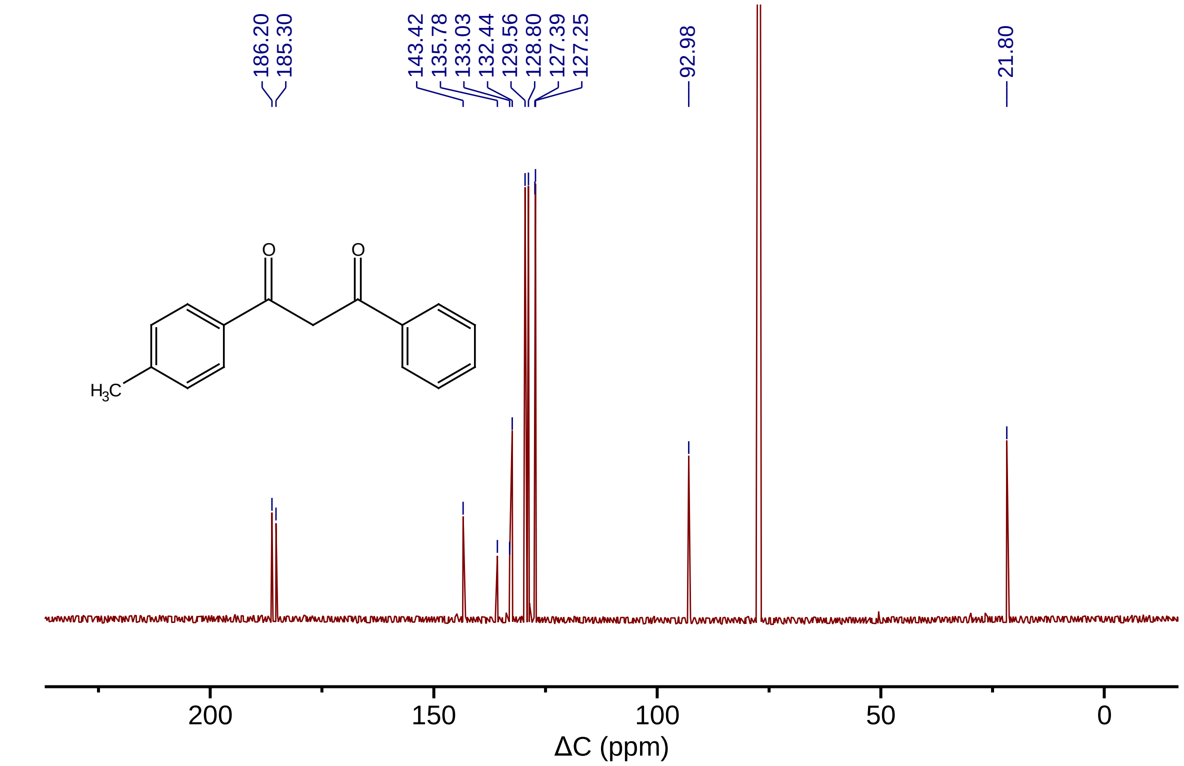

## 2.2 1-(Methoxyphenyl)-3-phenylpropane-1,3-dione (1c)

$^1H$ NMR (700 MHz, $CDCl_3$)

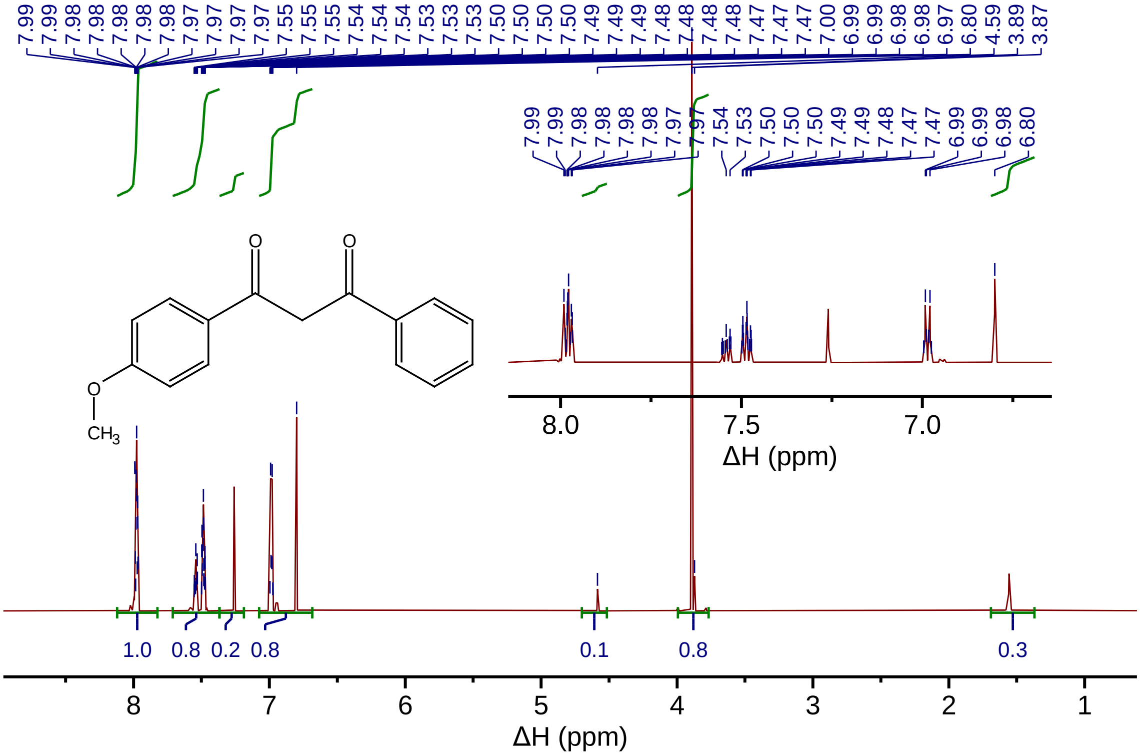


$^{13}C\{^1H\}$ NMR (176 MHz, $CDCl_3$)

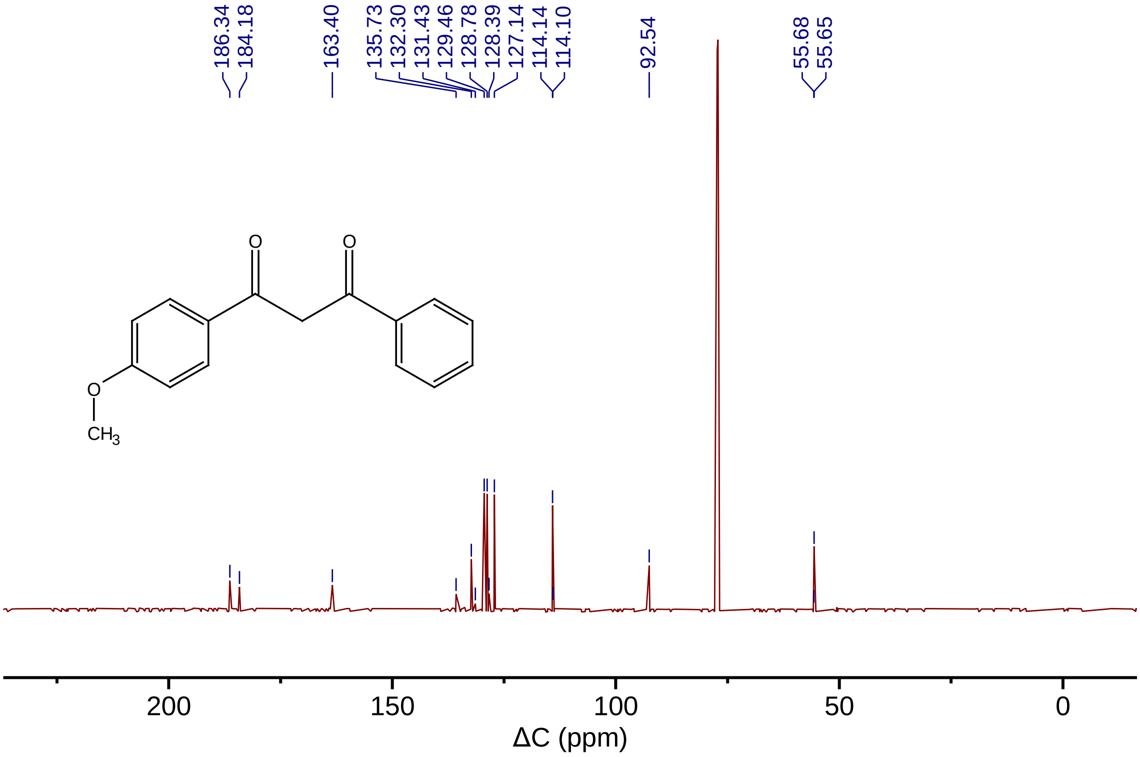

## 2.3 1-(Chlorophenyl)-3-phenylpropane-1,3-dione (1d)

$^1$H NMR (600 MHz, $CDCl_3$)

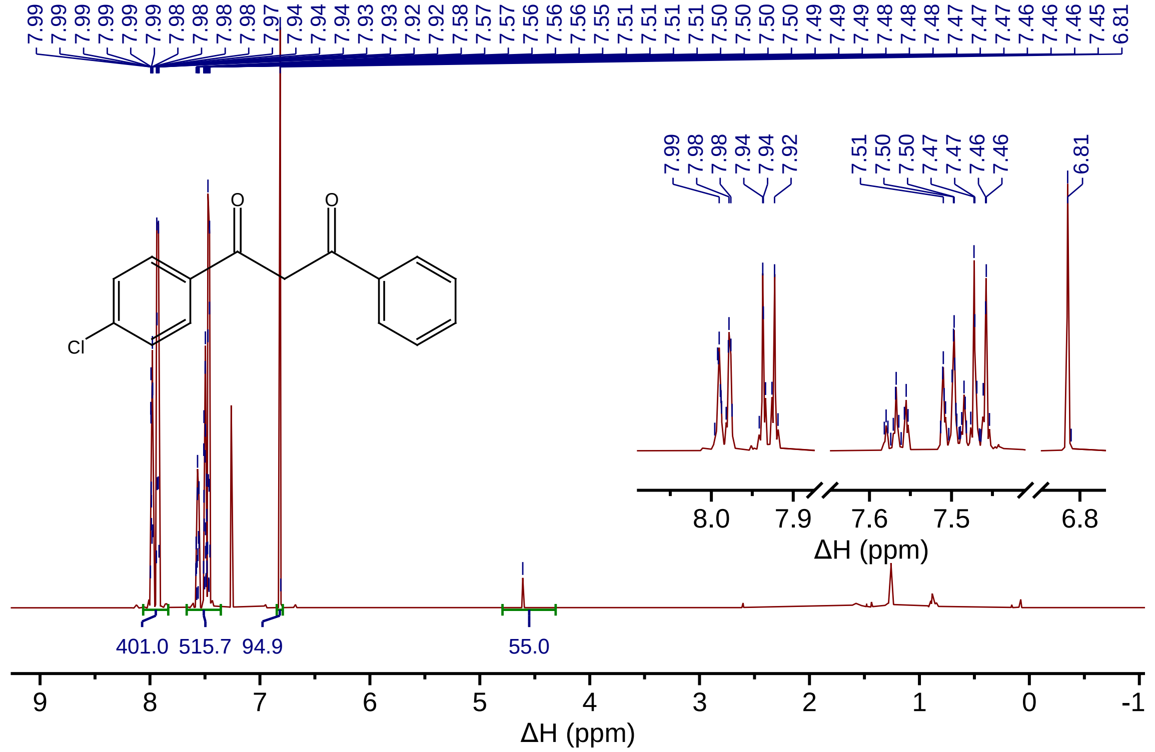


$^{13}$C{$^1$H} NMR (151 MHz, $CDCl_3$)

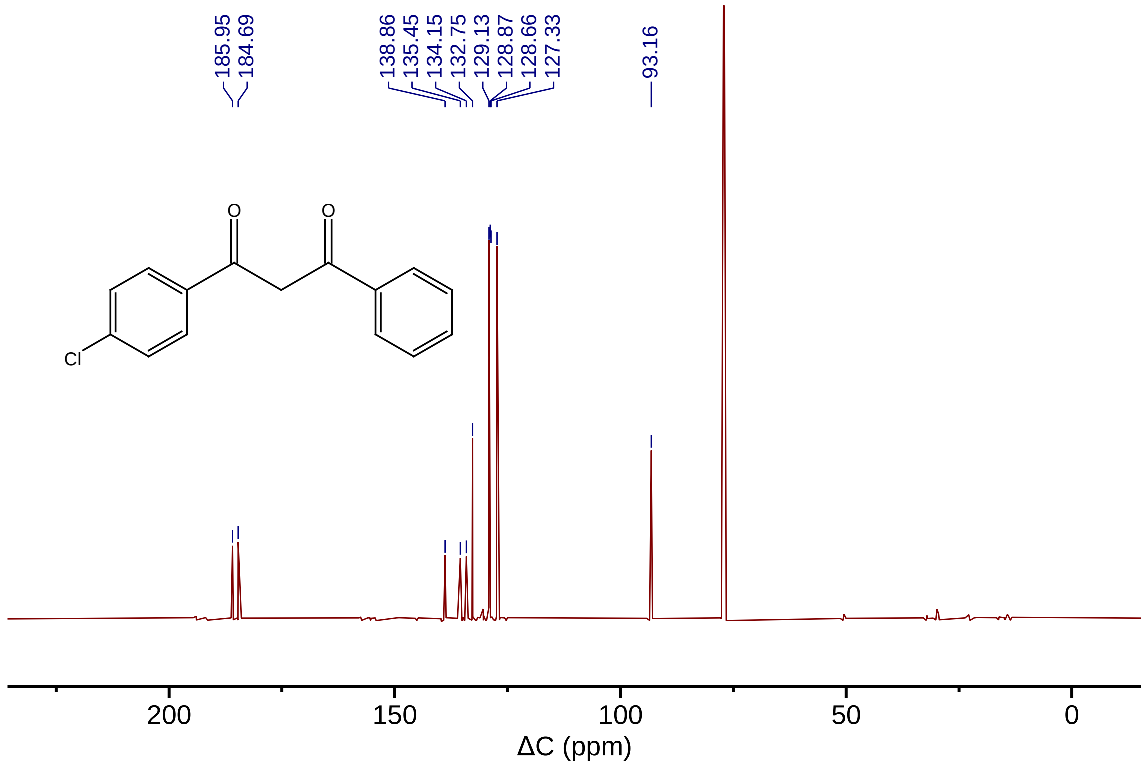

## 2.4 1-(Fluorophenyl)-3-phenylpropane-1,3-dione (1e)

$^{1}H$ NMR (700 MHz, $CDCl_3$)

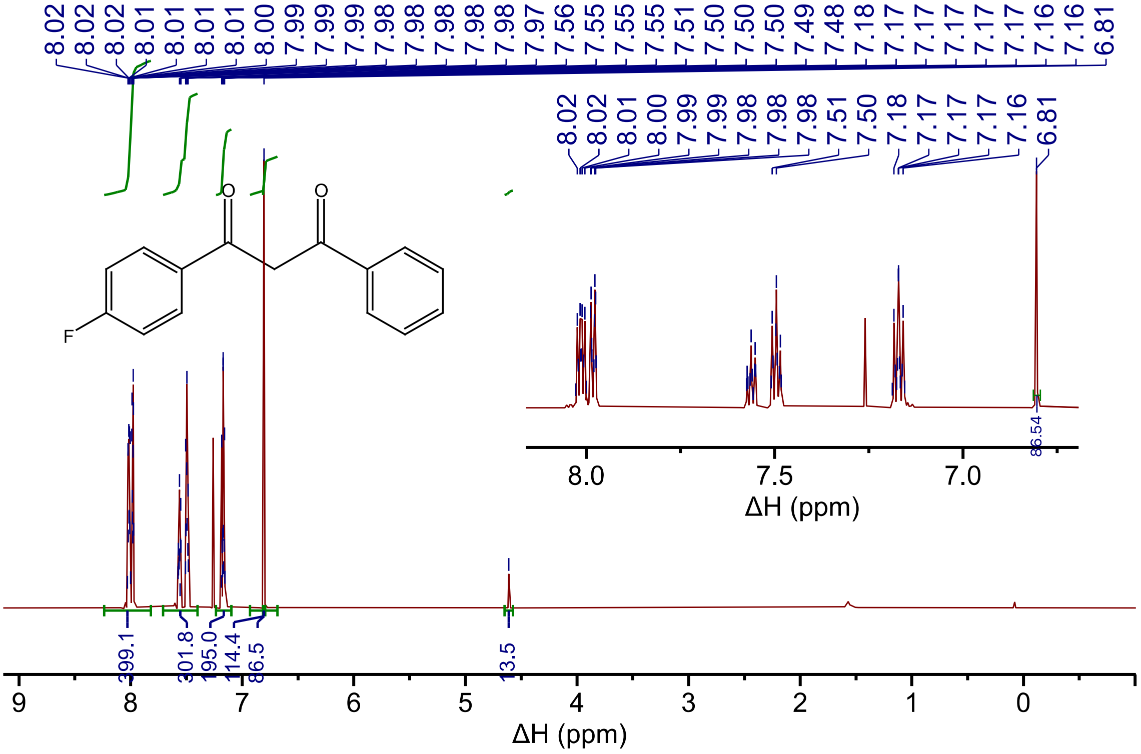


$^{19}F$ NMR (376 MHz, $CDCl_3$)

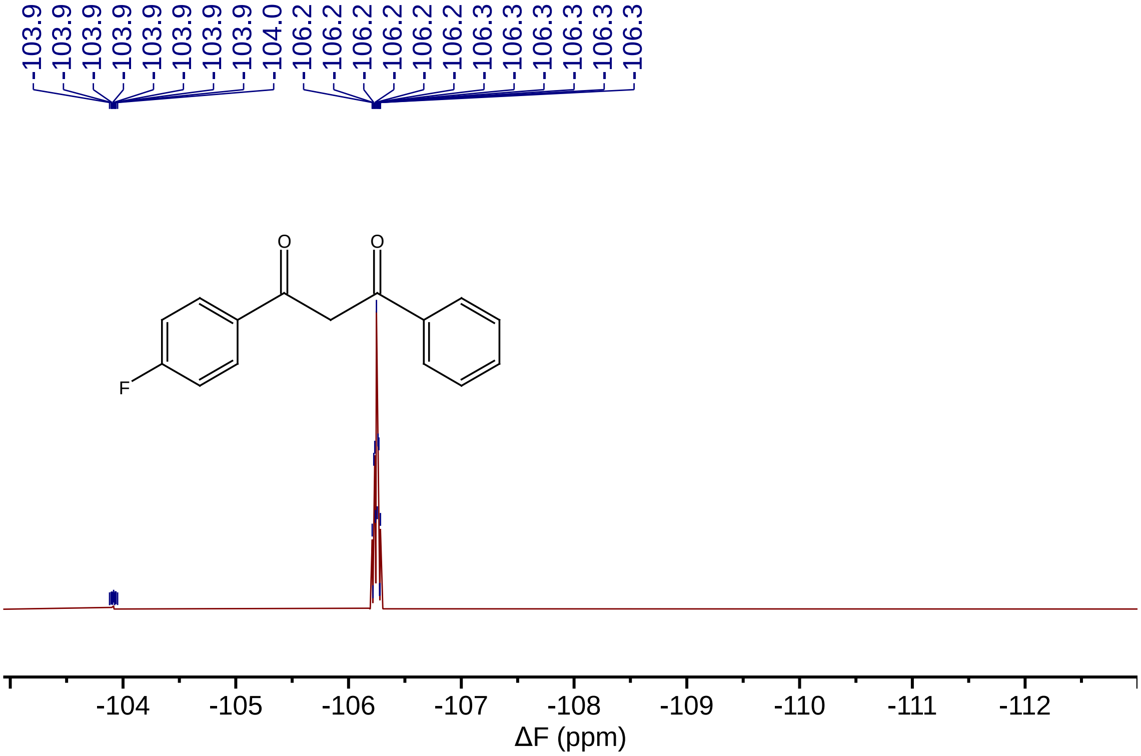

$^{13}C\{^{1}H\}$ NMR (176 MHz, $CDCl_3$)

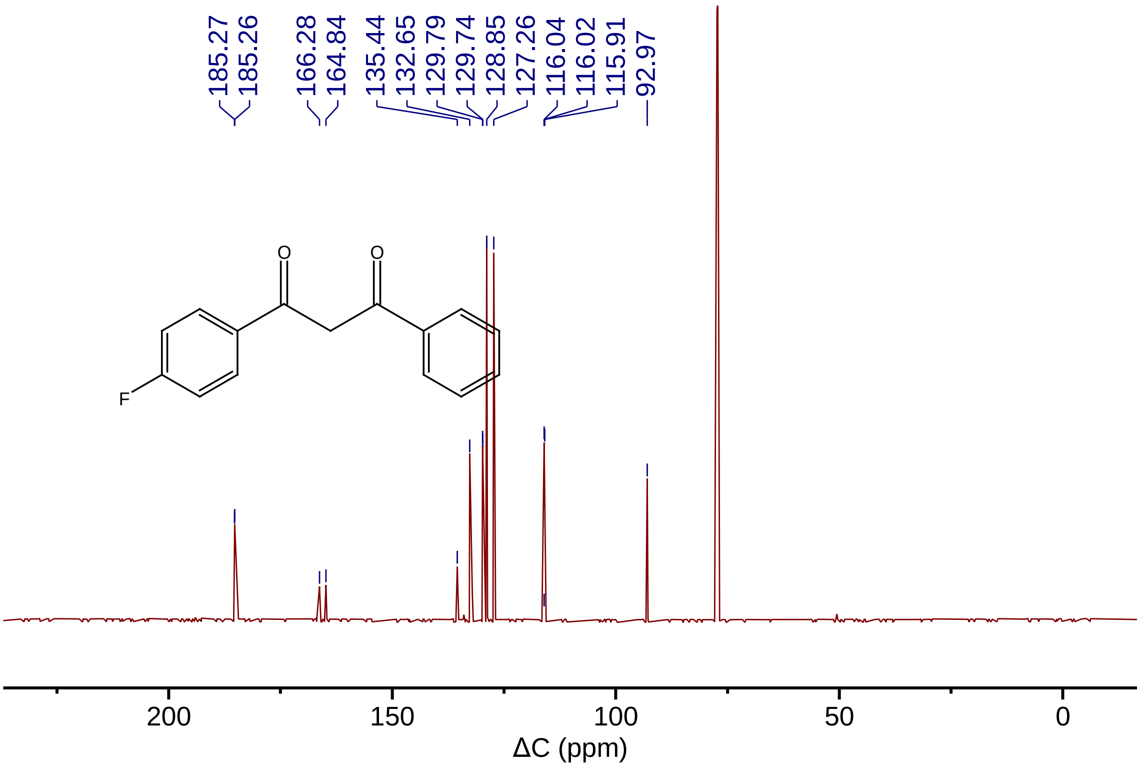

## 2.5 1-(Nitrophenyl)-3-phenylpropane-1,3-dione (1f)

$^1$H NMR (700 MHz, $CDCl_3$)

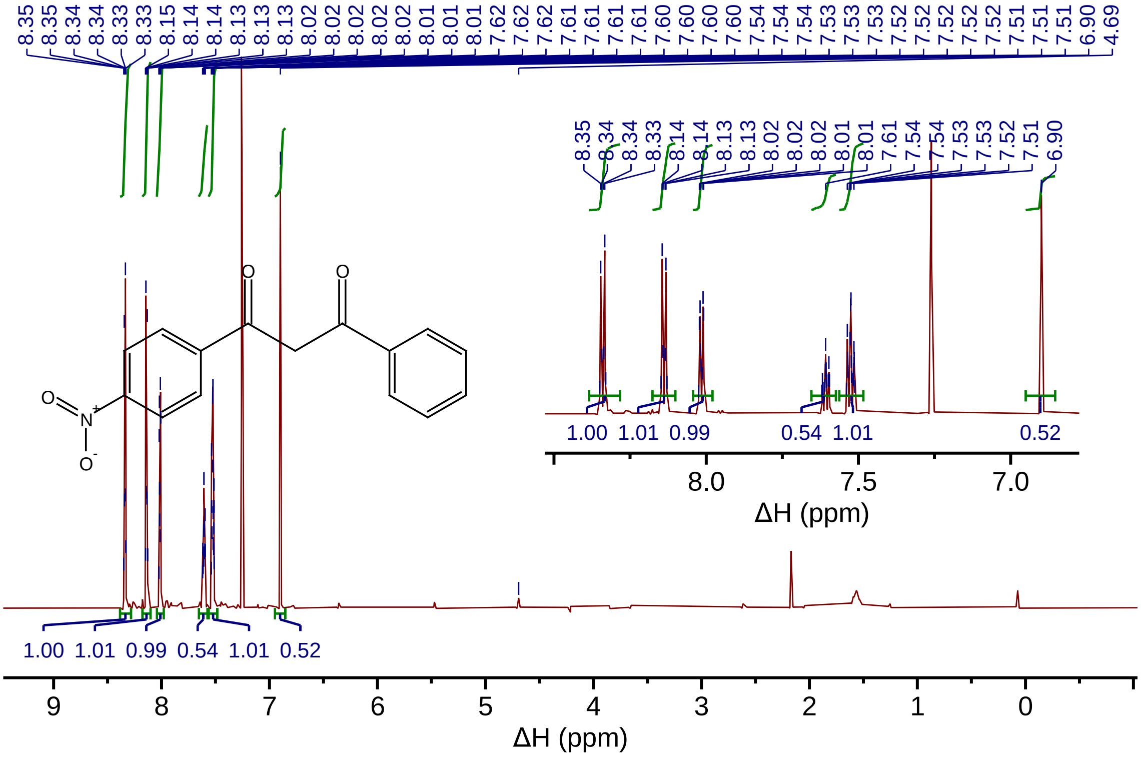


$^{13}$C{$^1$H} NMR (176 MHz, $CDCl_3$)

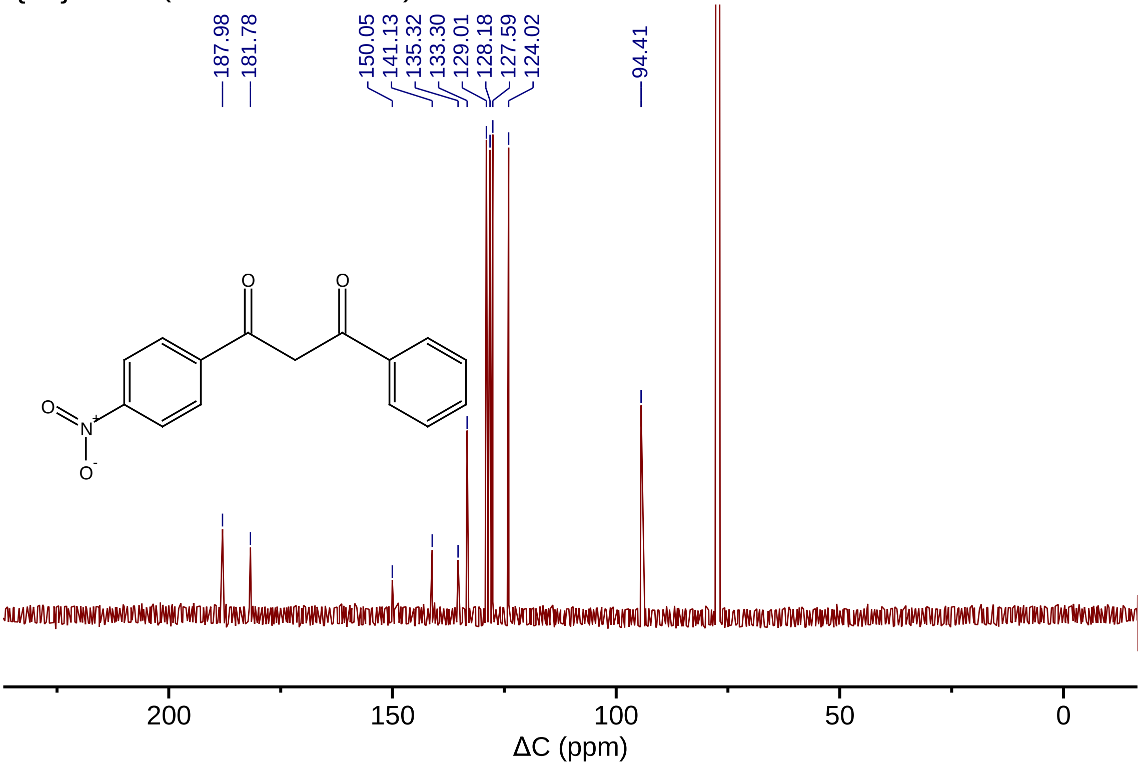

## 2.6 1,3-Bis(4-chlorophenyl)-propane-1,3-dione (1g)

$^{1}H$ NMR (700 MHz, $CDCl_3$)

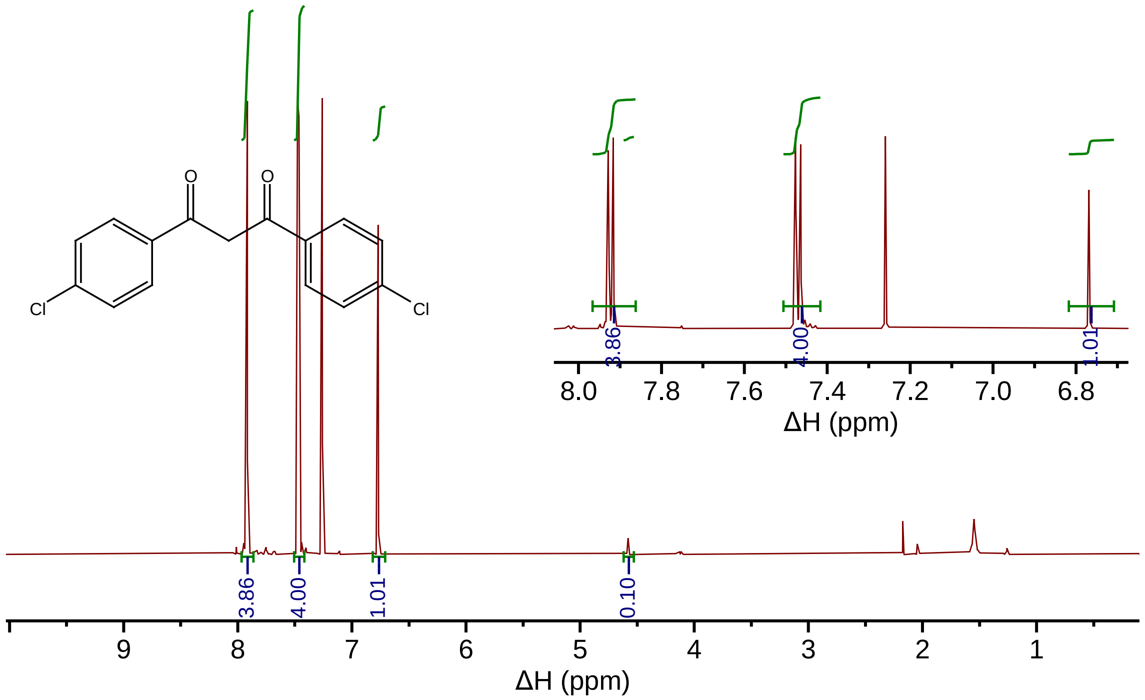


$^{13}C\{^{1}H\}$ NMR (176 MHz, $CDCl_3$)

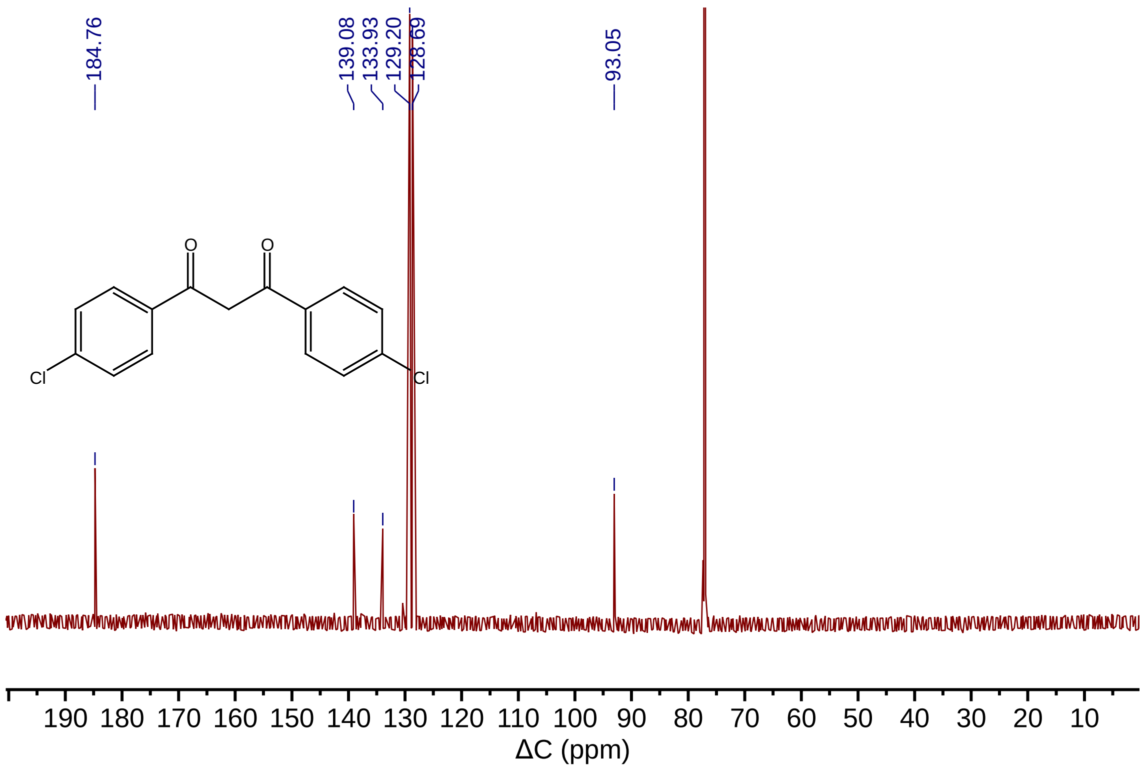

## 2.7 1,3-Bis(fluorophenyl)-propane-1,3-dione (1h)

$^{1}$H NMR (700 MHz, CDCl$_3$)

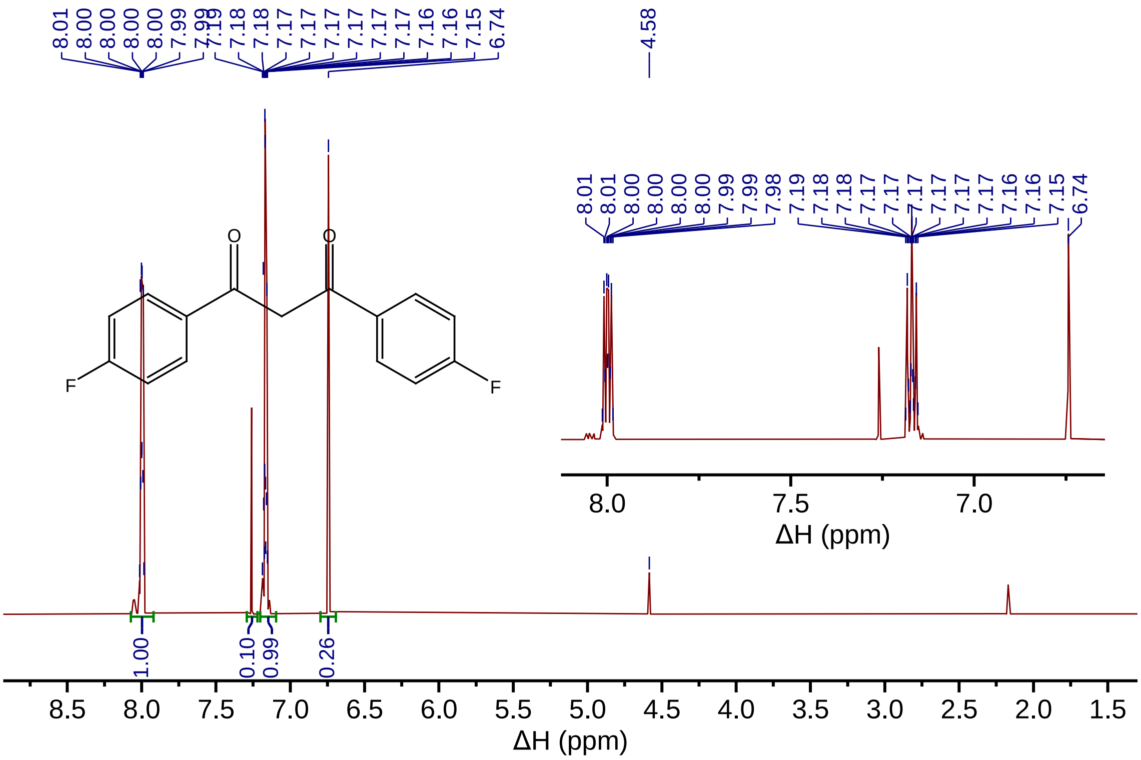


$^{19}$F NMR (376 MHz, CDCl$_3$)

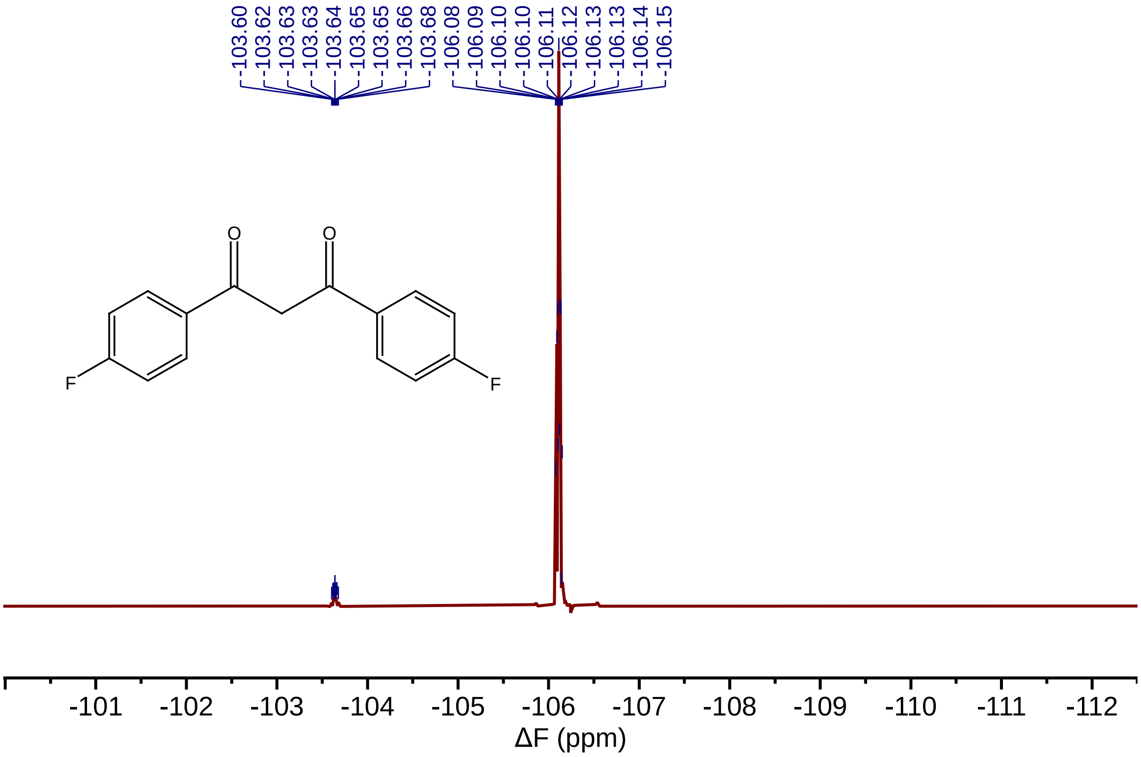

$^{13}C\{^{1}H\}$ NMR (176 MHz, $CDCl_3$)

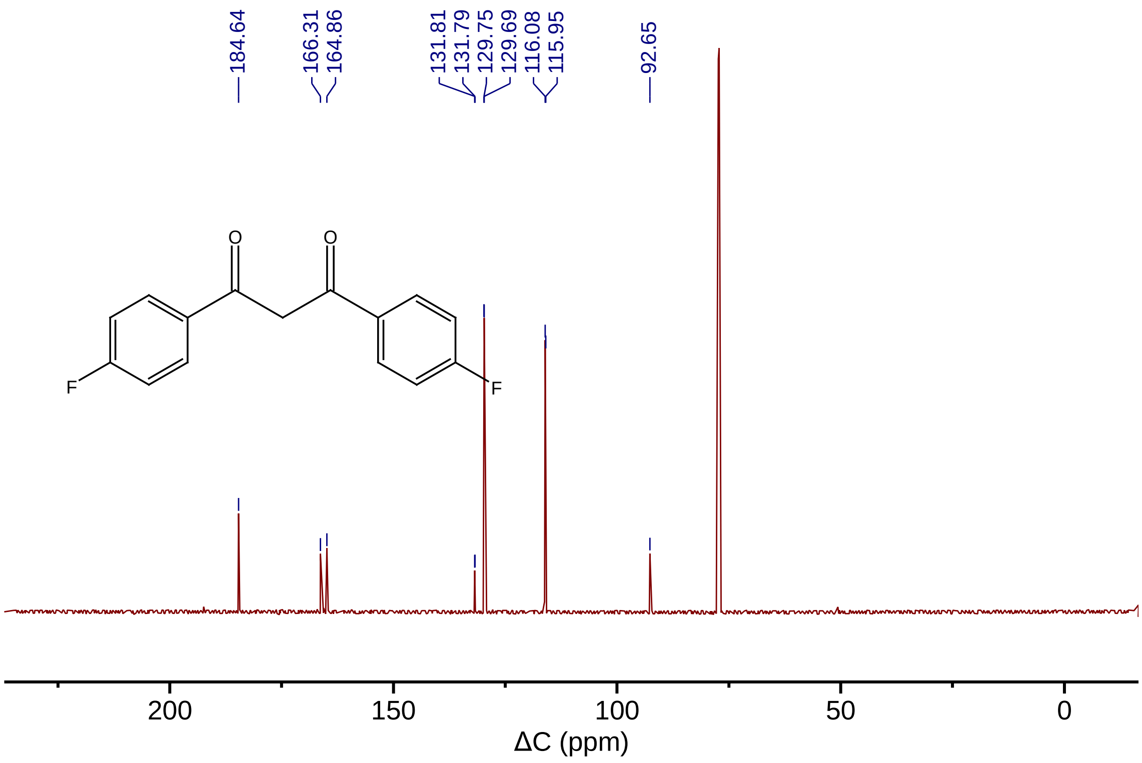

## 2.8 1,3-Bis(nitrophenyl)-propane-1,3-dione (1i)

$^1$H NMR (700 MHz, $CDCl_3$)

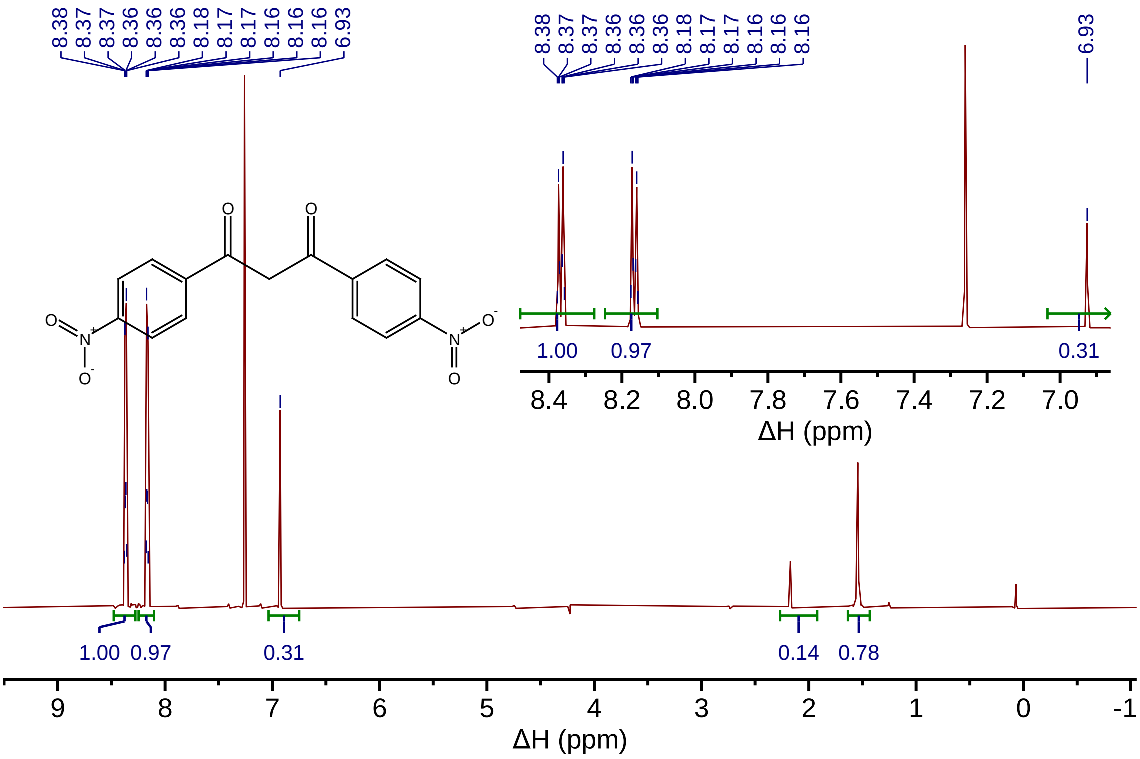


$^{13}$C{$^1$H} NMR (176 MHz, $CDCl_3$)

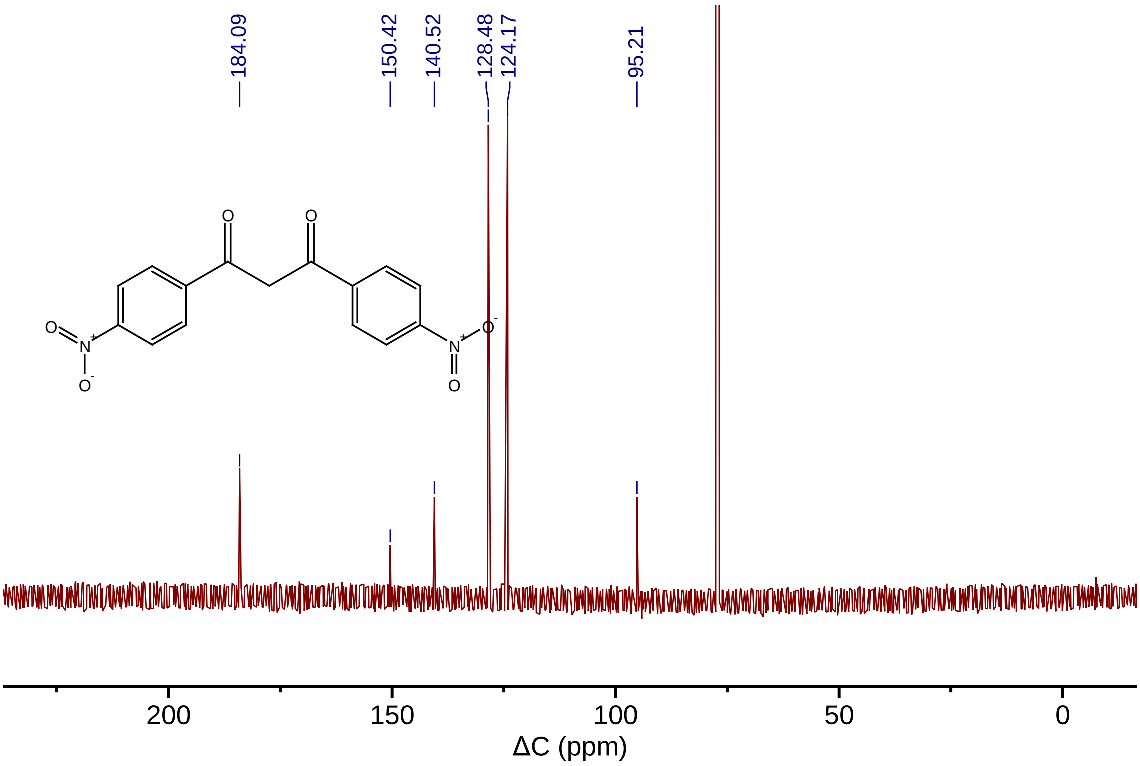

## 2.9 2,2-Difluorophenylpropane-1,3-dione (3a)

$^1H$ NMR (600 MHz, $CDCl_3$)

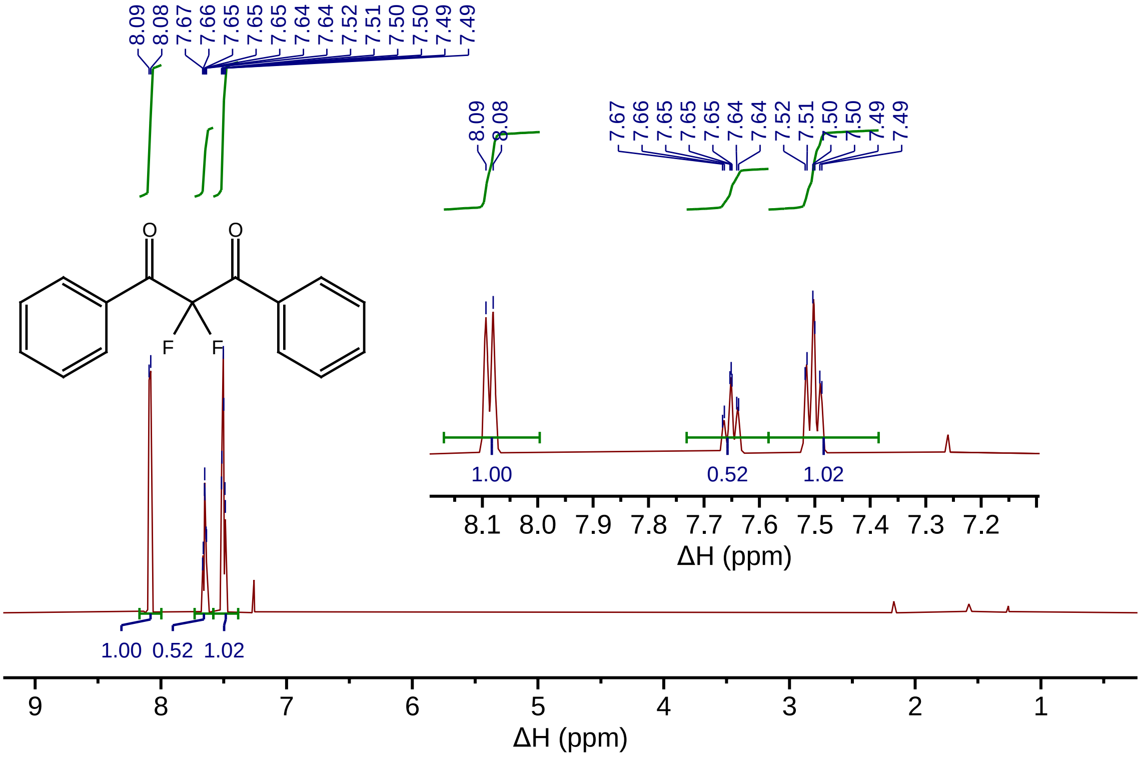


$^{19}F$ NMR (376 MHz, $CDCl_3$)

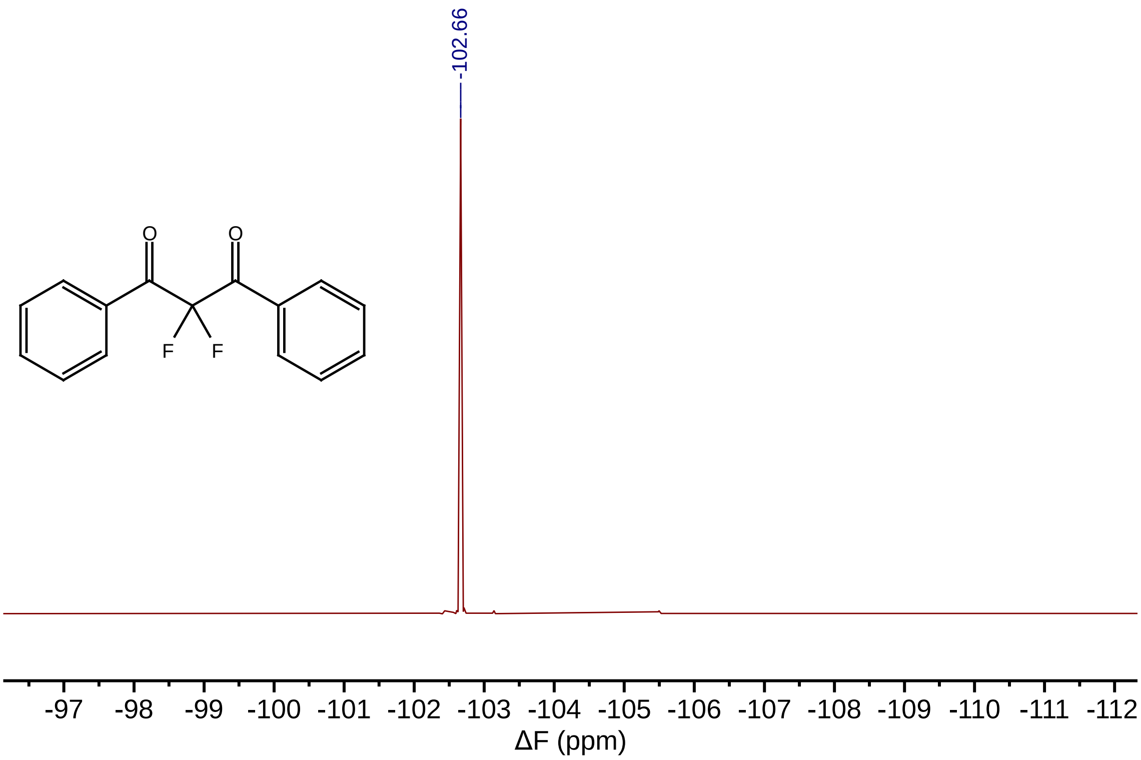

$^{13}C\{^1H\}$ NMR (151 MHz, $CDCl_3$)

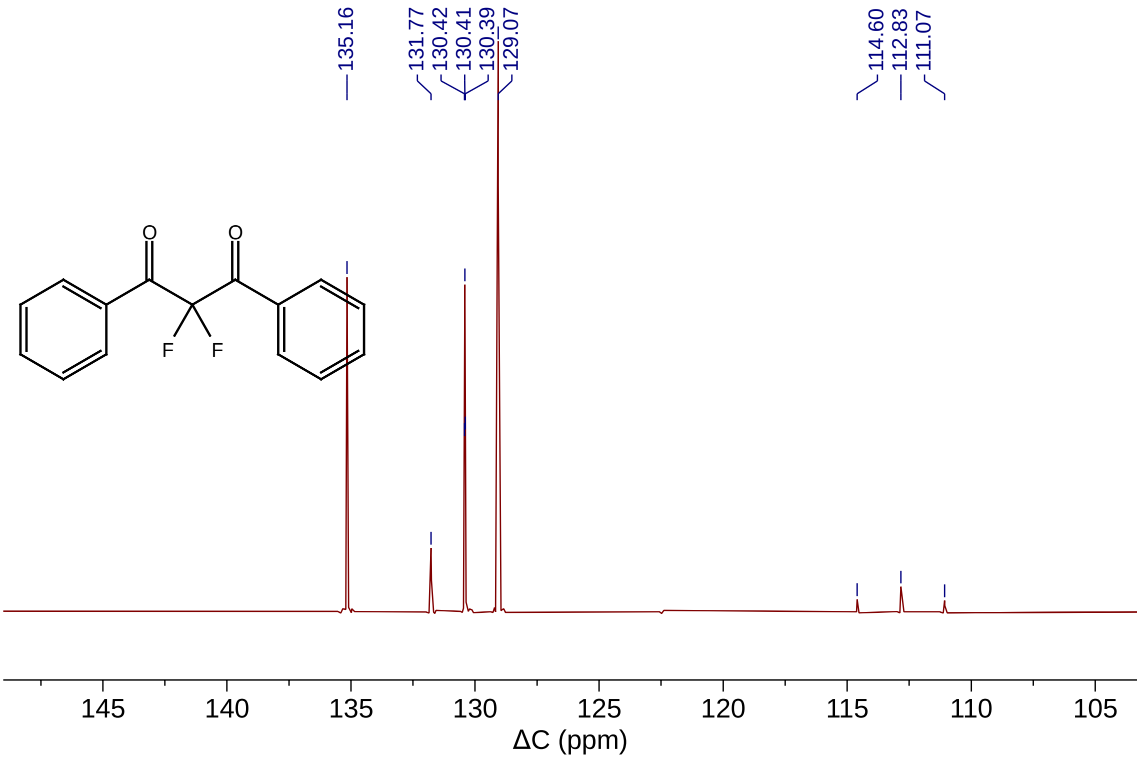

## 2.10 2,2-Difluoro-1-(4-chlorophenyl)-3-phenylpropane-1,3-dione (3d)

$^{1}H$ NMR (700 MHz, $CDCl_3$)

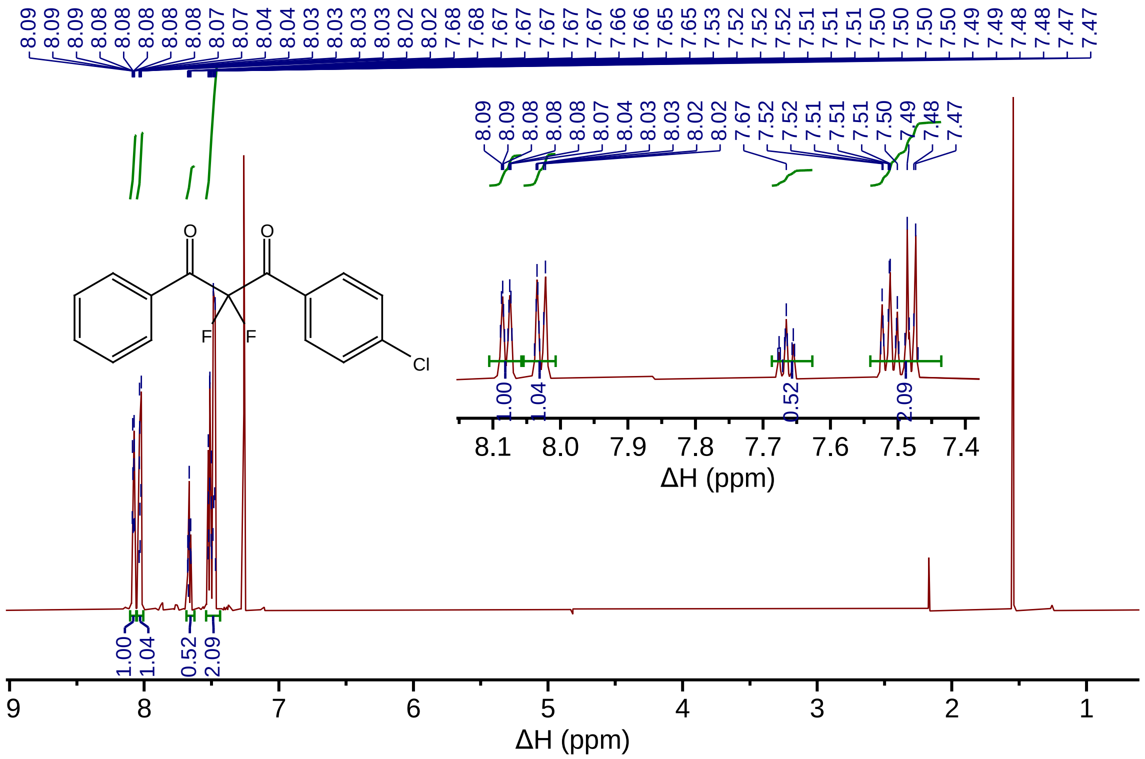

$^{19}F$ NMR (376 MHz, $CDCl_3$)

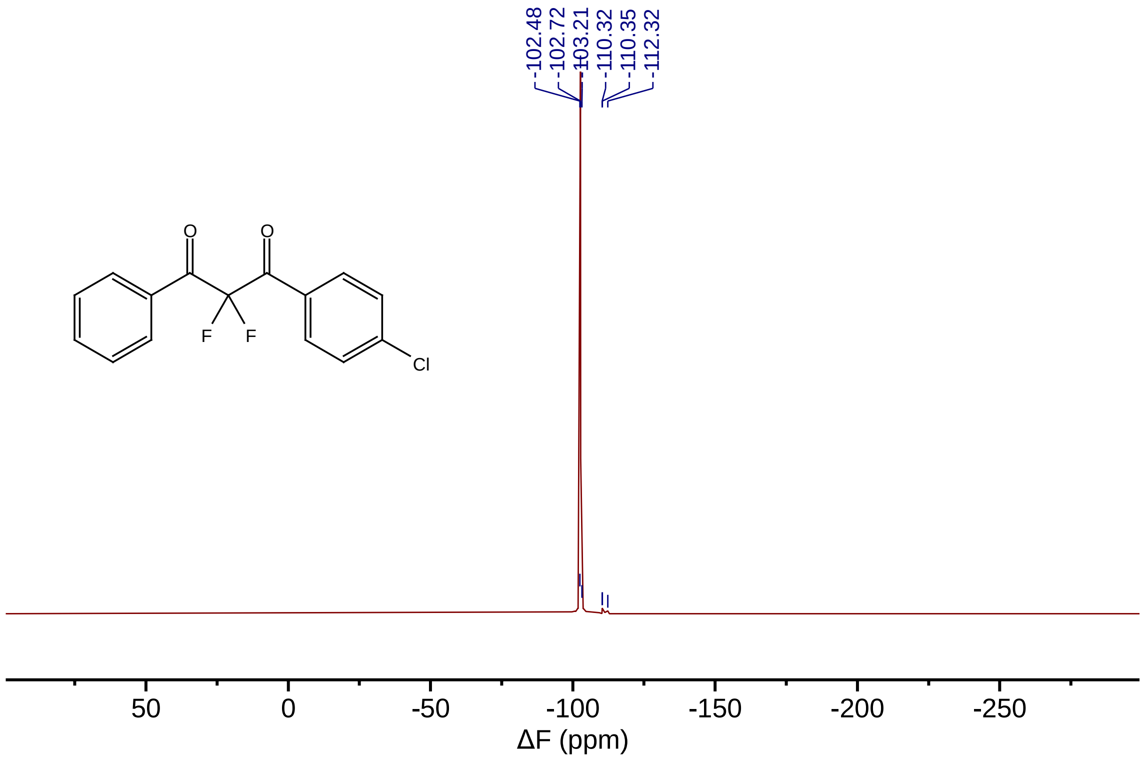

$^{13}C\{^{1}H\}$ NMR (176 MHz, $CDCl_3$)

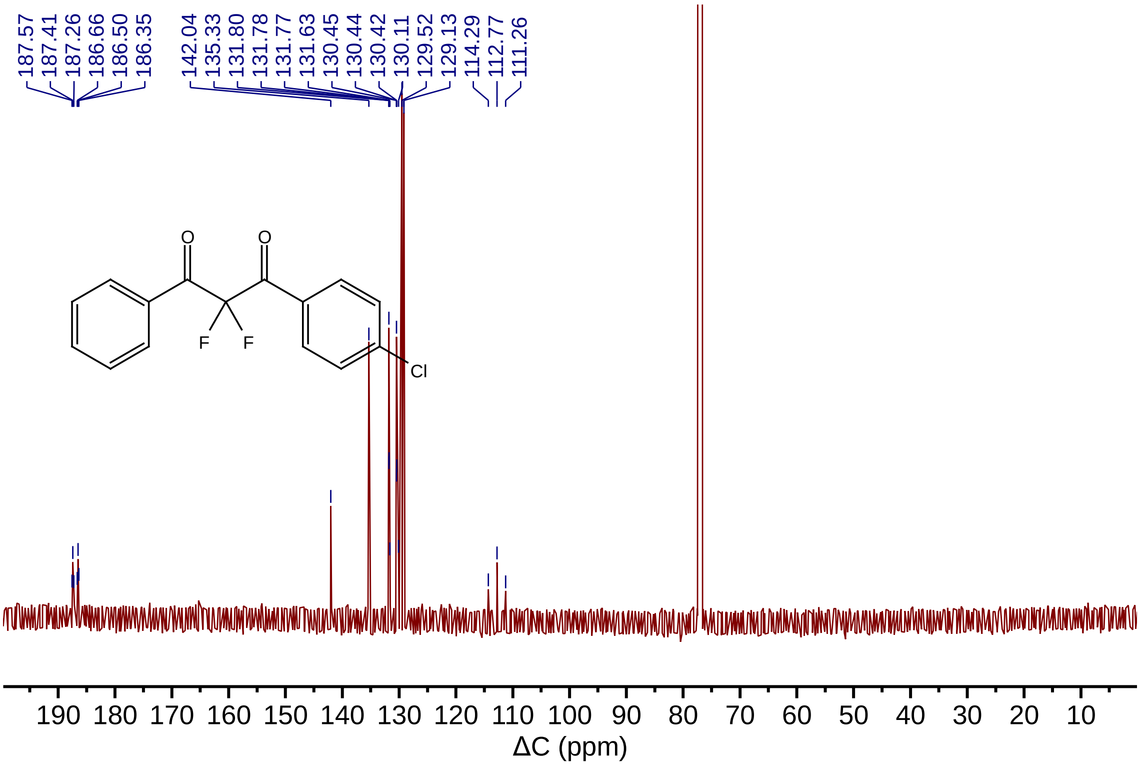

## 2.11 2,2-Difluoro-1-(4-fluorophenyl)-3-phenylpropane-1,3-dione (3e)

$^{1}$H NMR (700 MHz, $CDCl_3$)

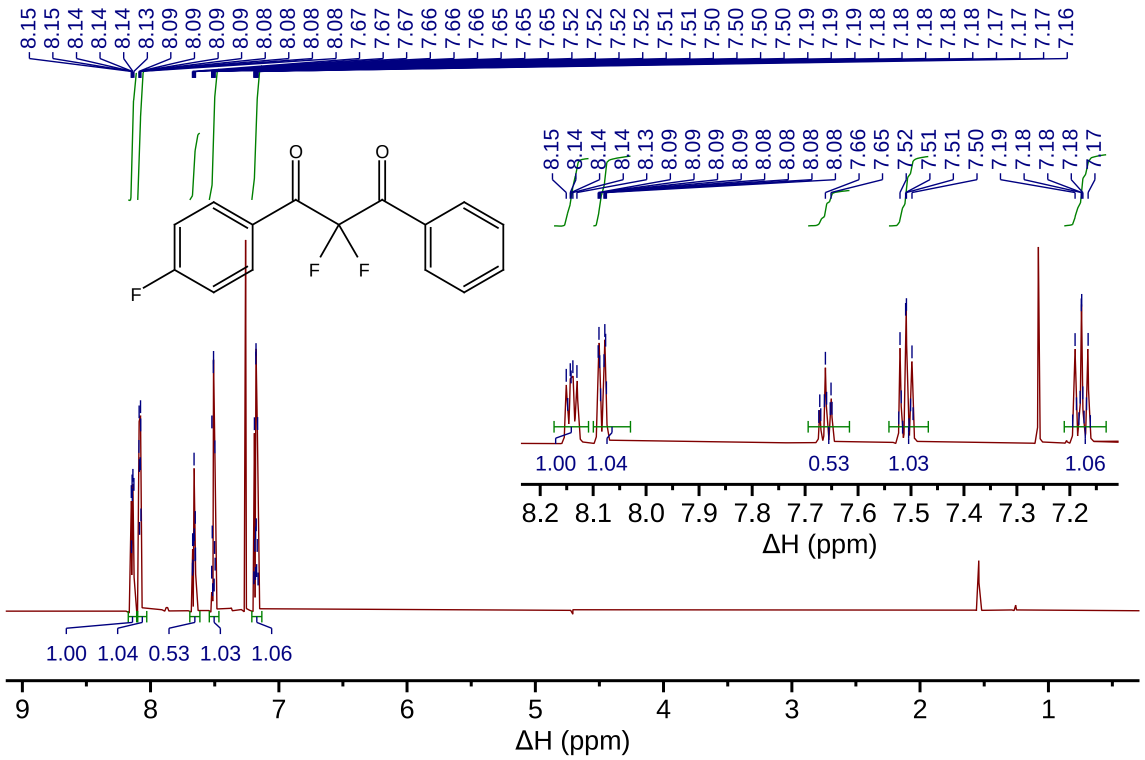


$^{19}$F NMR (376 MHz, $CDCl_3$)

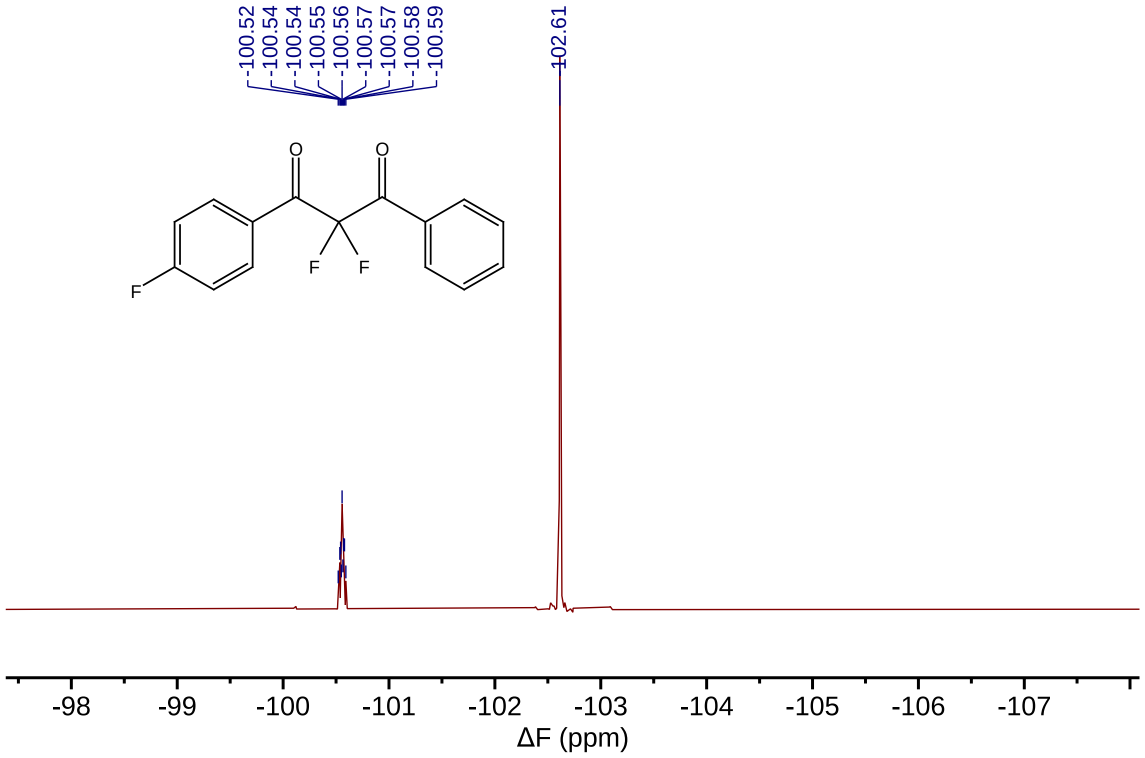

$^{13}C\{^1H\}$ NMR (176 MHz, $CDCl_3$)

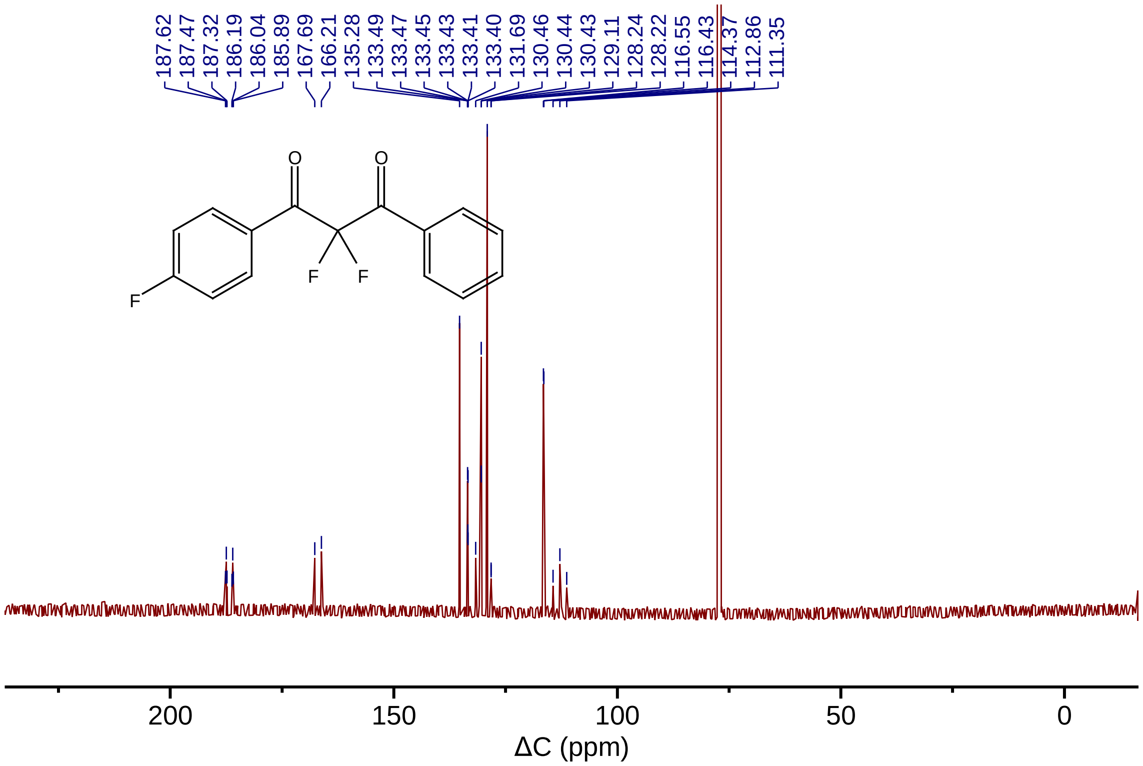

## 2.12 2,2-Difluoro-1-(nitrophenyl)-3-phenylpropane-1,3-dione (3f)

$^{1}H$ NMR (700 MHz, $CDCl_3$)

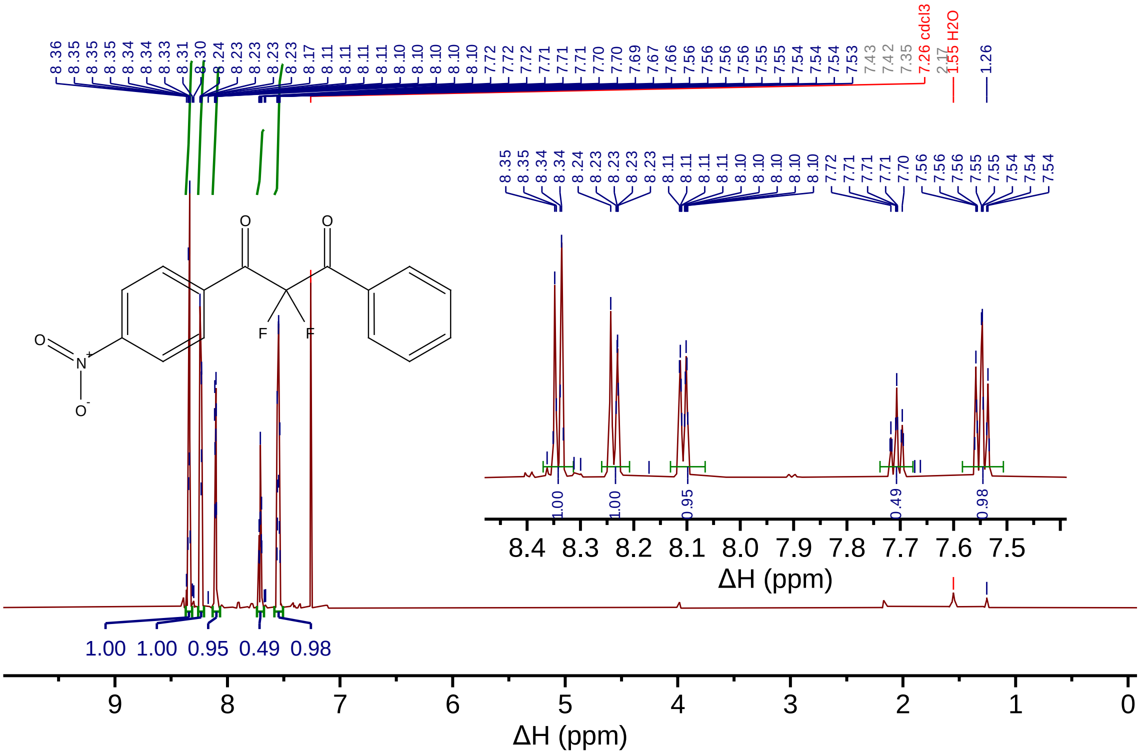

$^{19}F$ NMR (376 MHz, $CDCl_3$)

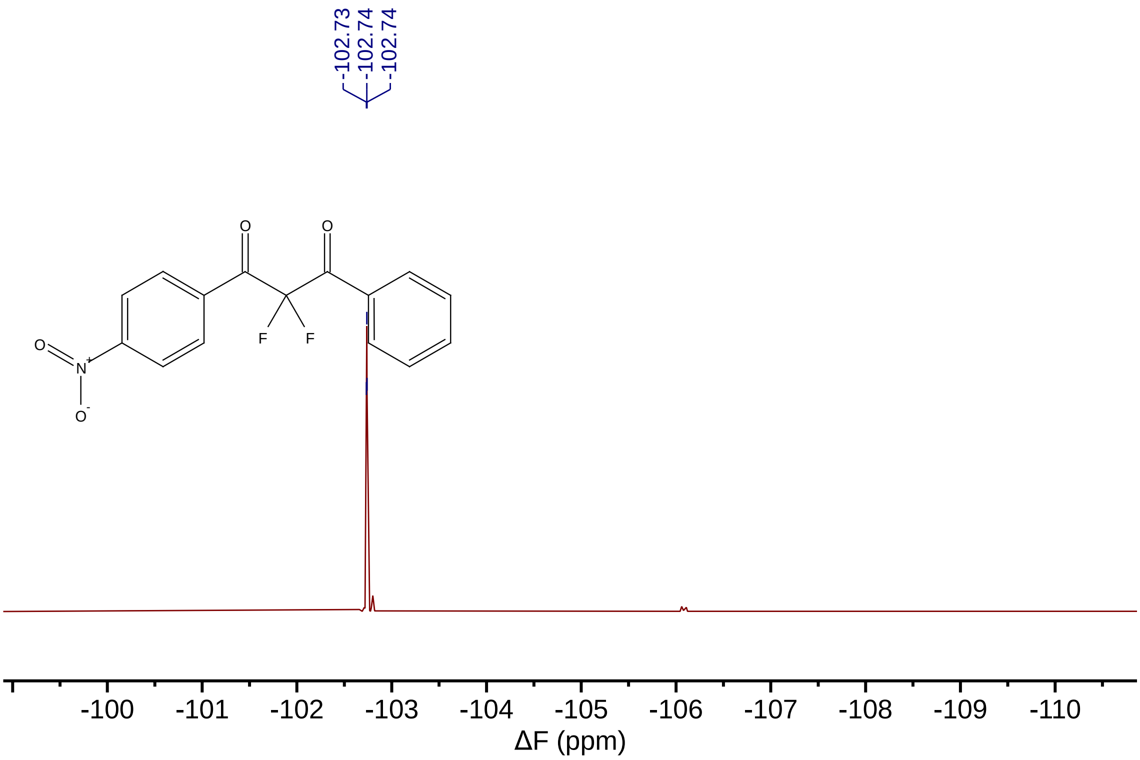

$^{13}C\{^1H\}$ NMR (151 MHz, $CDCl_3$)

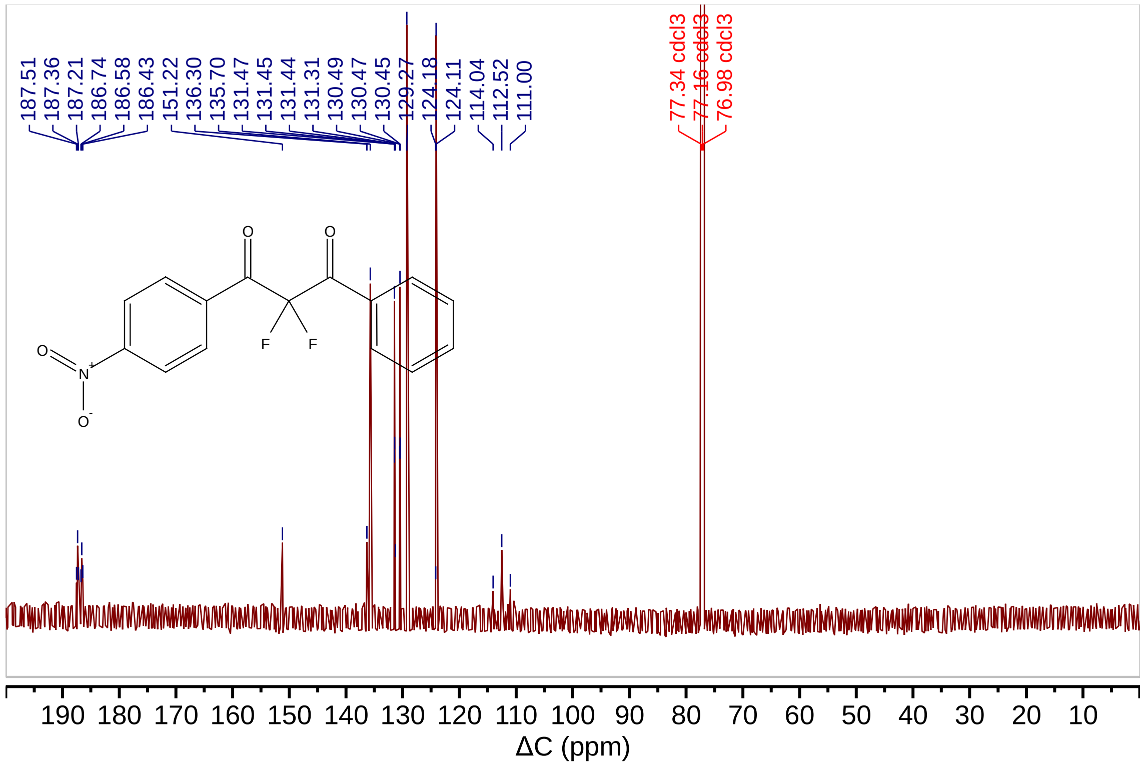

## 2.13 2,2-Difluoro-1,3-bis(chlorophenyl)propane-1,3-dione (3g)

$^{1}$H NMR (700 MHz, $CDCl_3$)

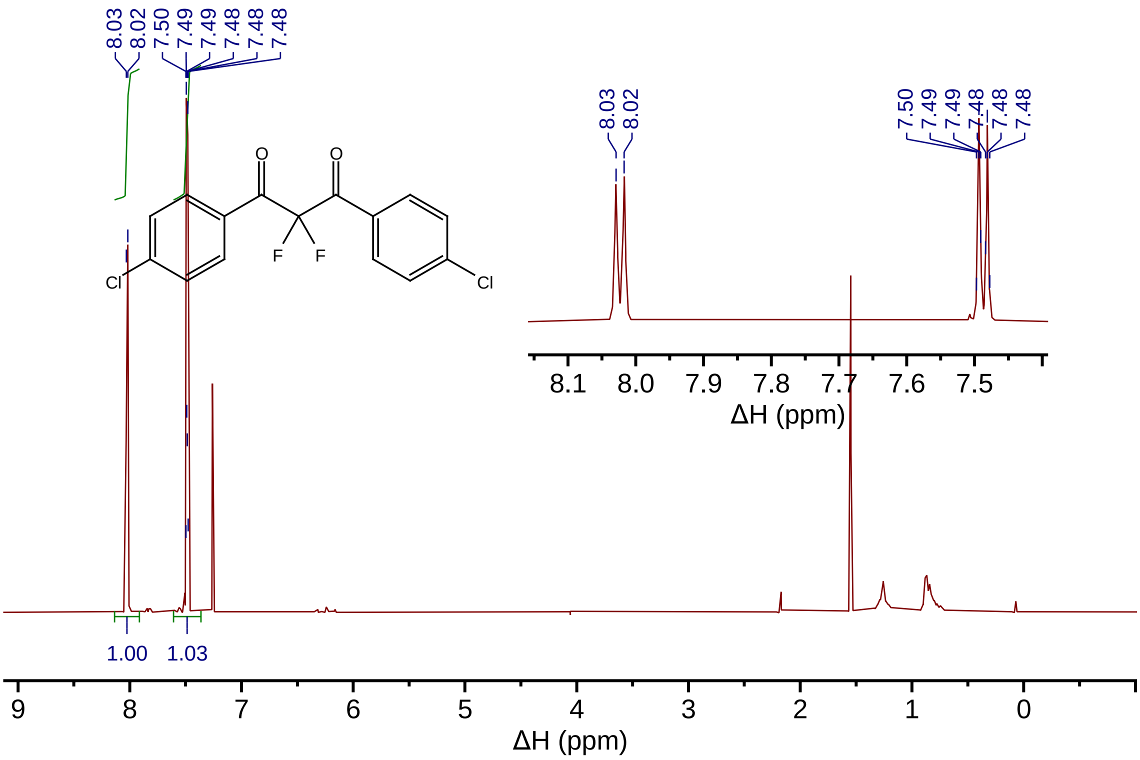


$^{19}$F NMR (376 MHz, $CDCl_3$)

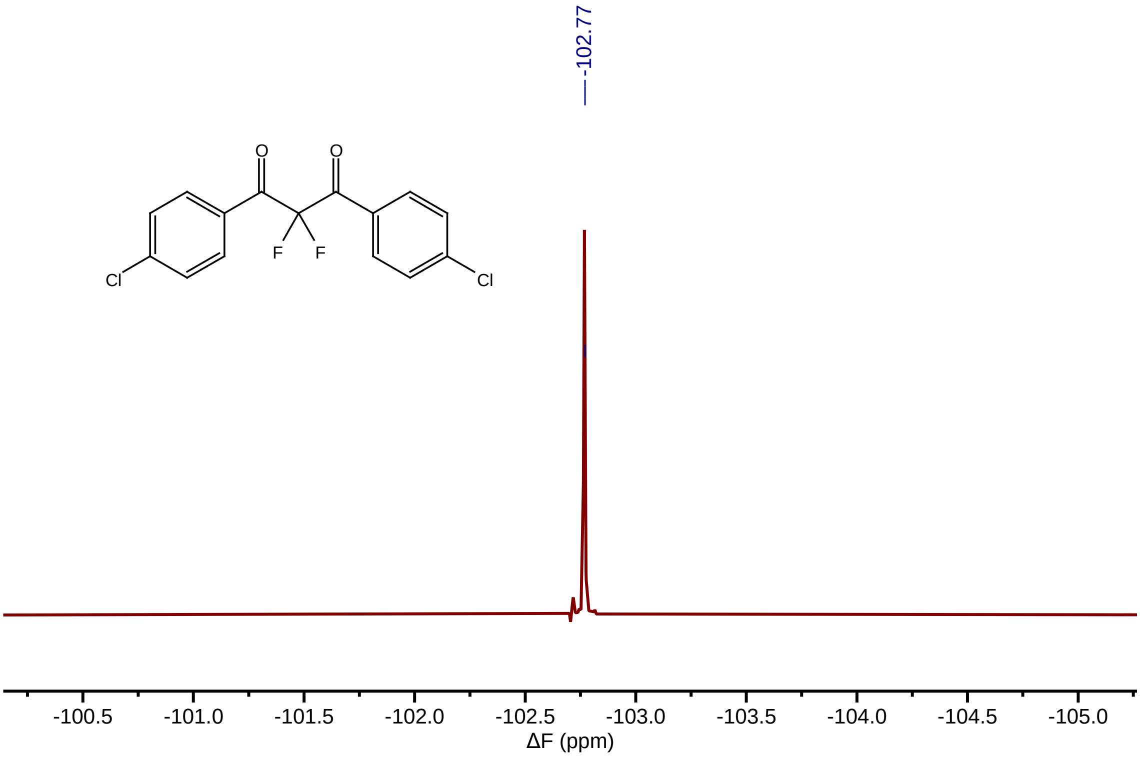

$^{13}C\{^{1}H\}$ NMR (176 MHz, $CDCl_3$)

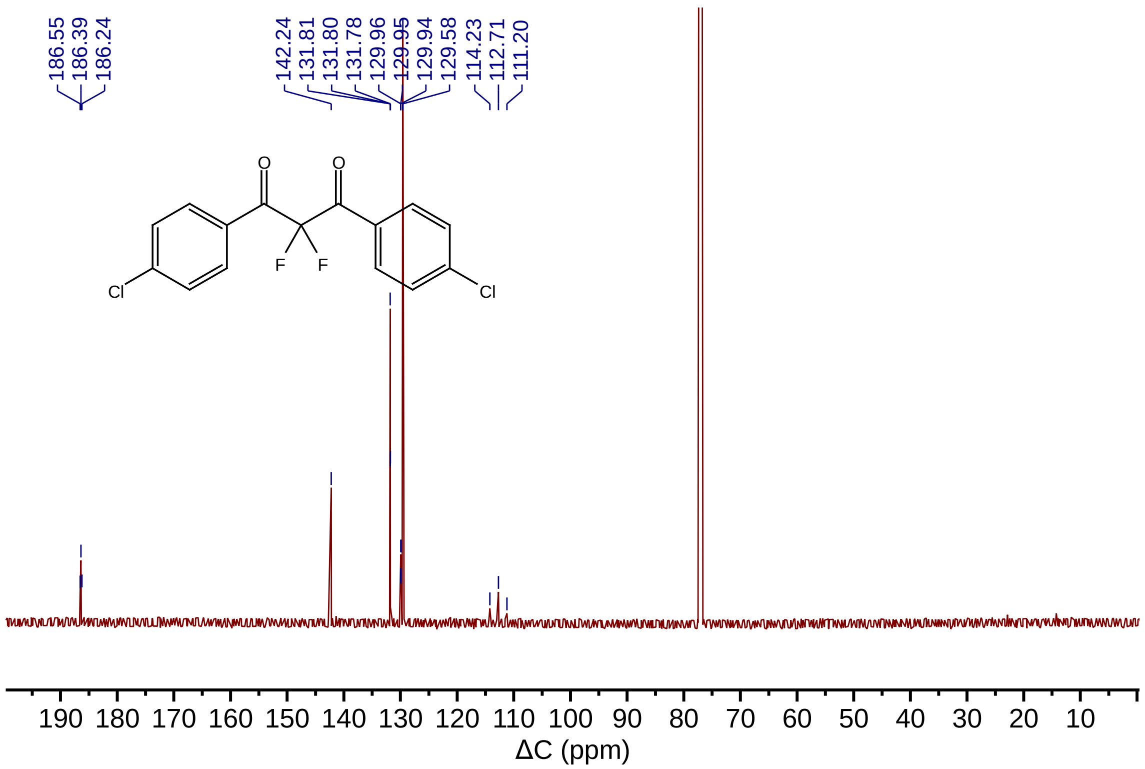

## 2.14 2,2-Difluoro-1,3-bis(fluorophenyl)propane-1,3-dione (3h)

$^1$H NMR (500 MHz, $CDCl_3$)

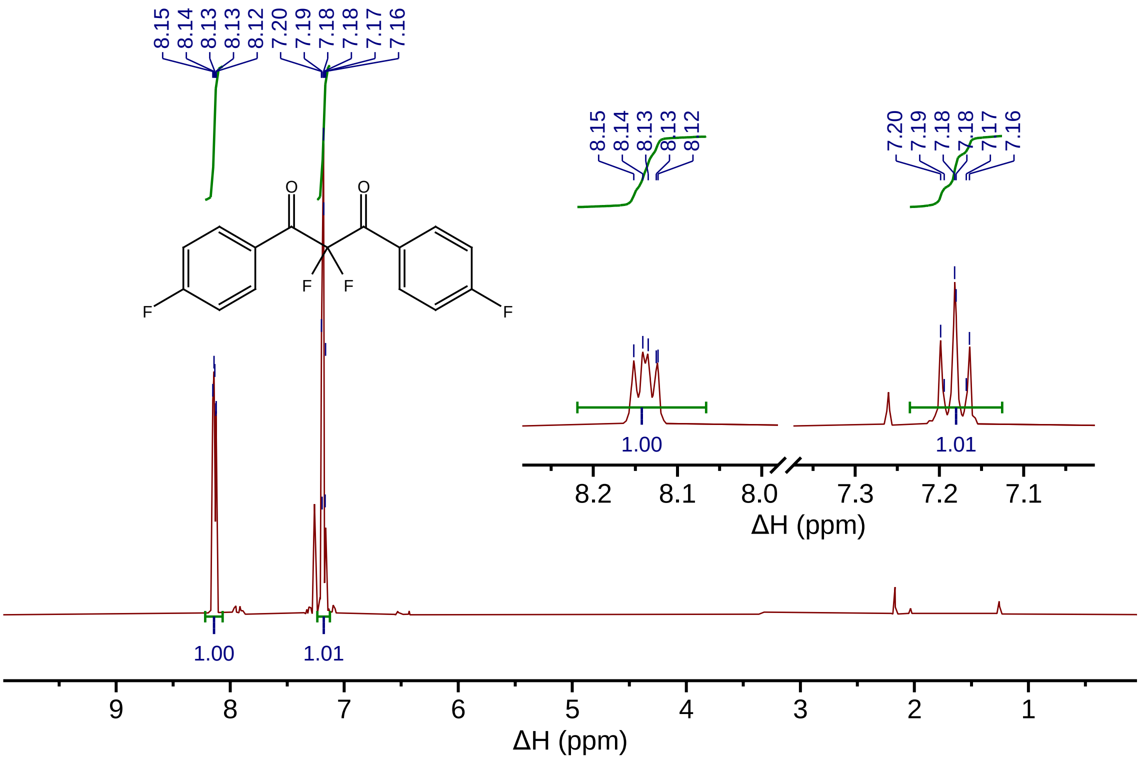


$^{19}$F NMR (376 MHz, $CDCl_3$)

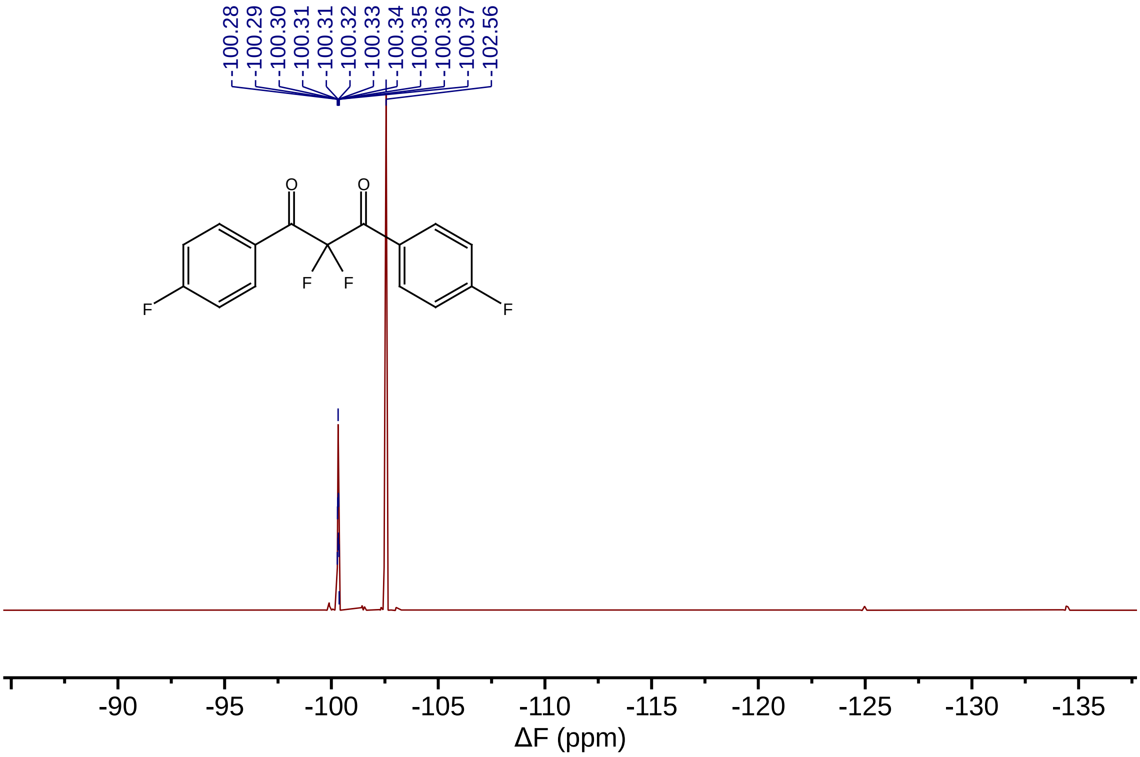

$^{13}C\{^{1}H\}$ NMR (176 MHz, $CDCl_3$)

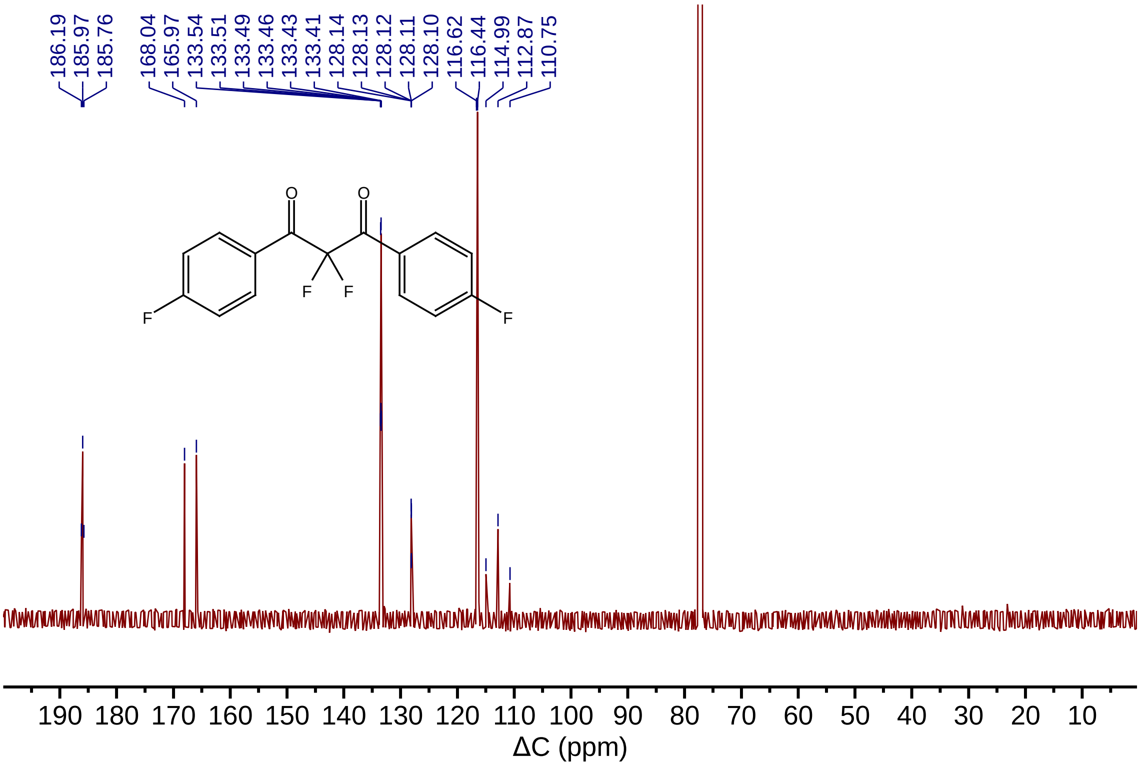

## 2.15 2,2-Difluoro-1,3-bis(nitrophenyl)propane-1,3-dione (3i)

$^{1}H$ NMR (700 MHz, $CDCl_3$)

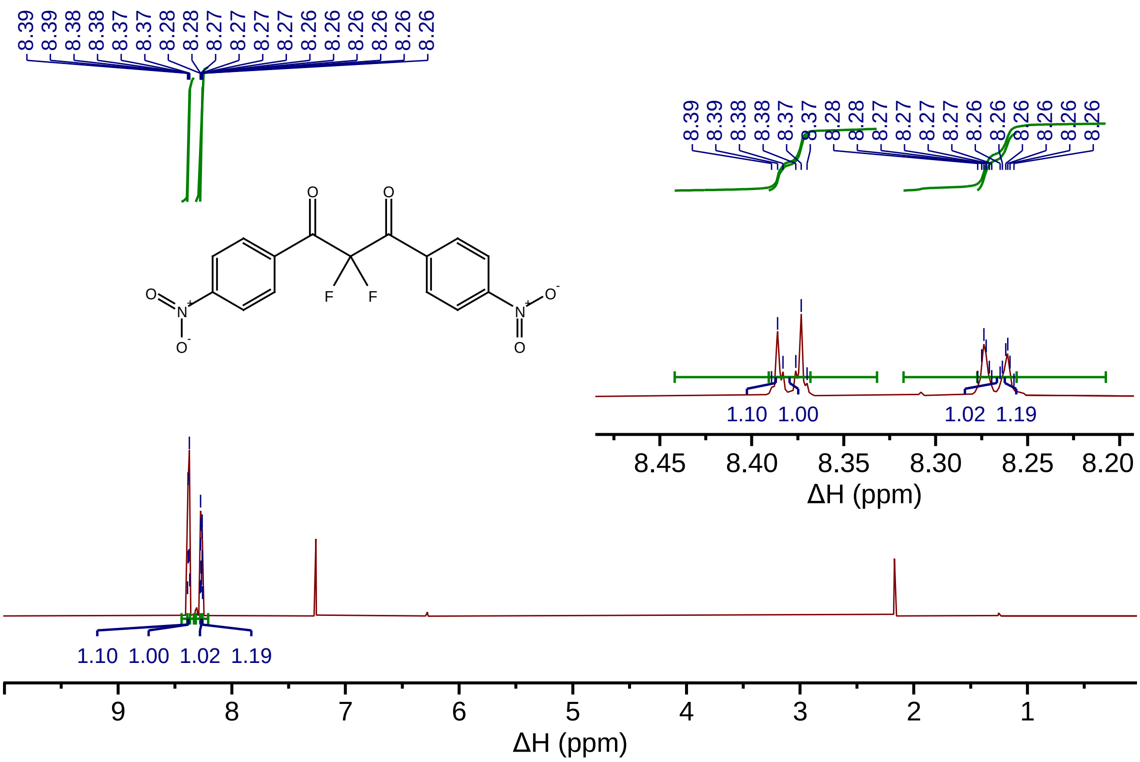


$^{19}F$ NMR (376 MHz, $CDCl_3$)

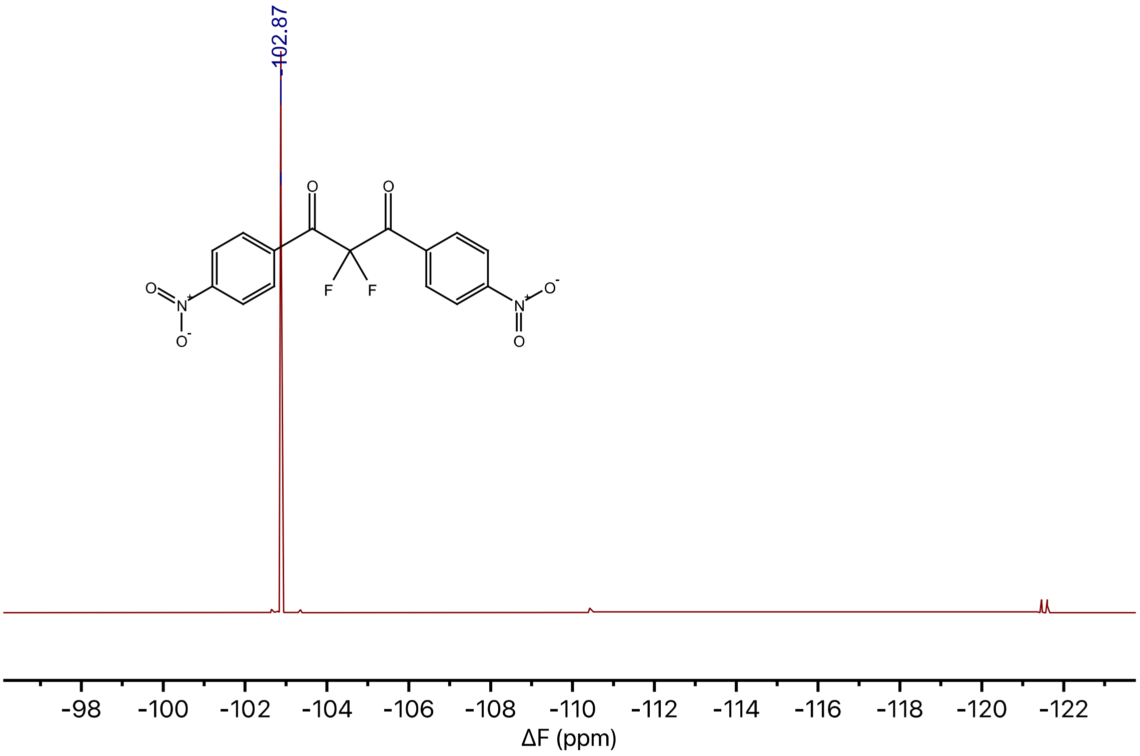

$^{13}C\{^{1}H\}$ NMR (176 MHz, $CDCl_3$)

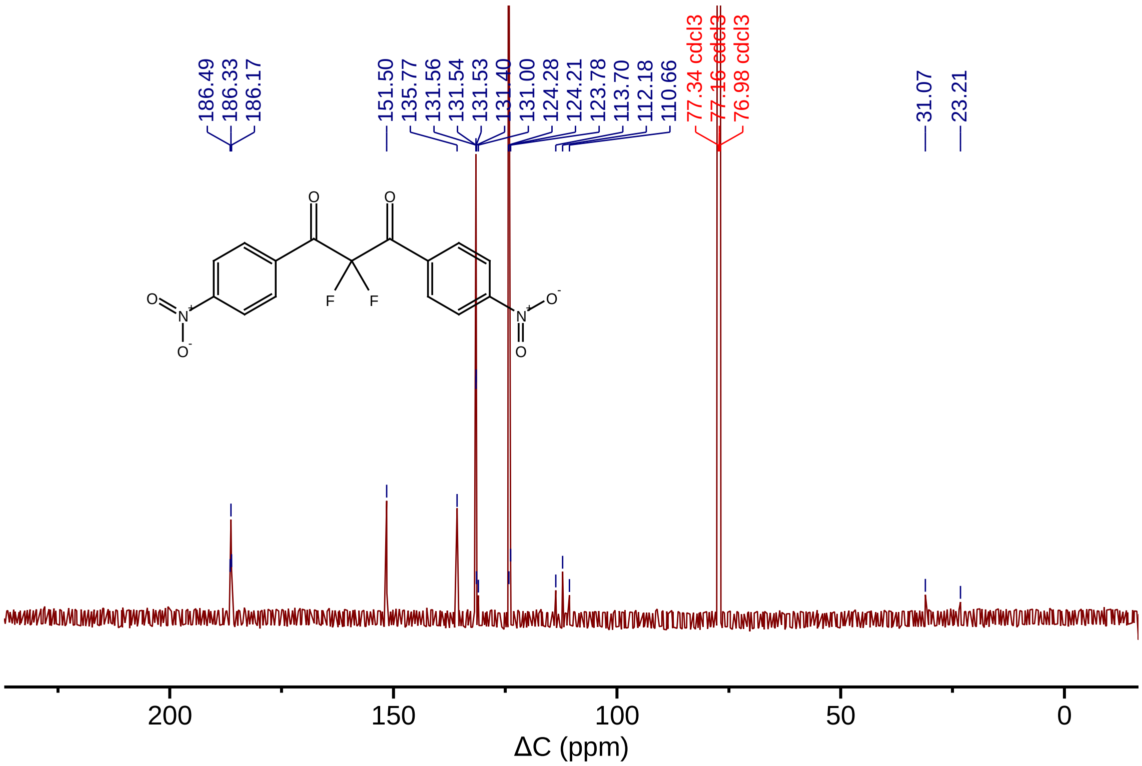

## 2.16 Ethyl (4-methoxy)benzoylacetate (4b)

$^1$H NMR (700 MHz, $CDCl_3$)

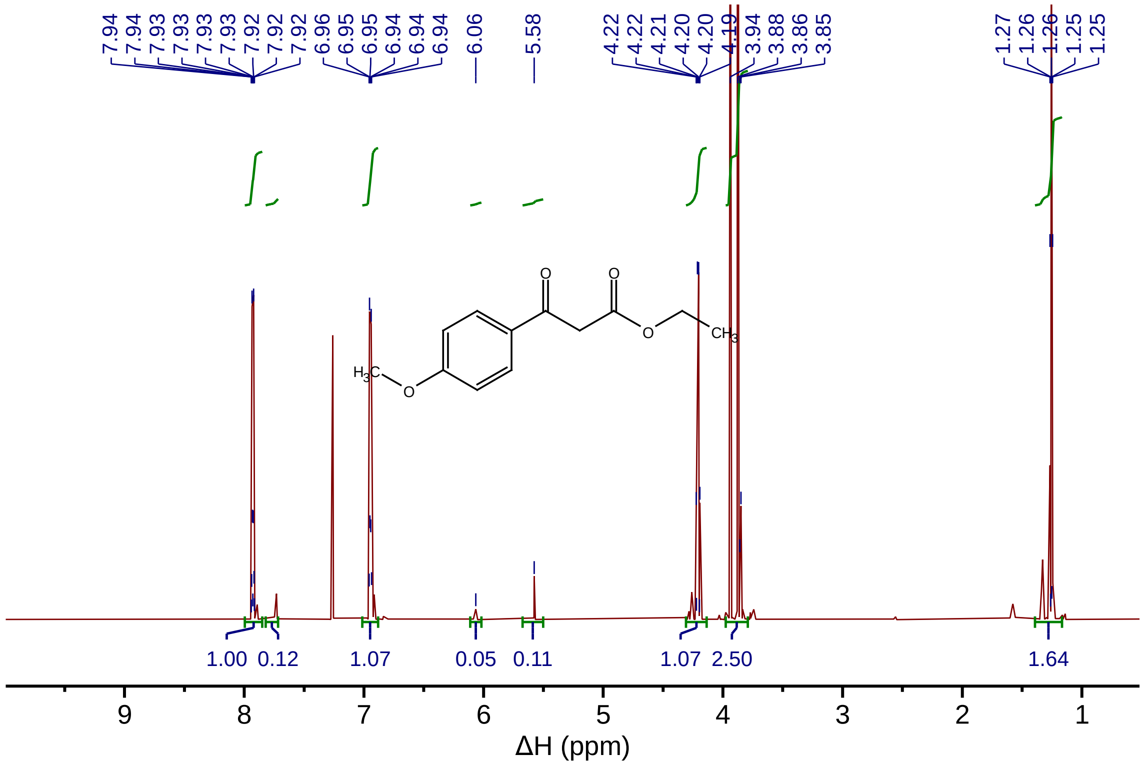

$^{13}C\{^1H\}$ NMR (176 MHz, $CDCl_3$)

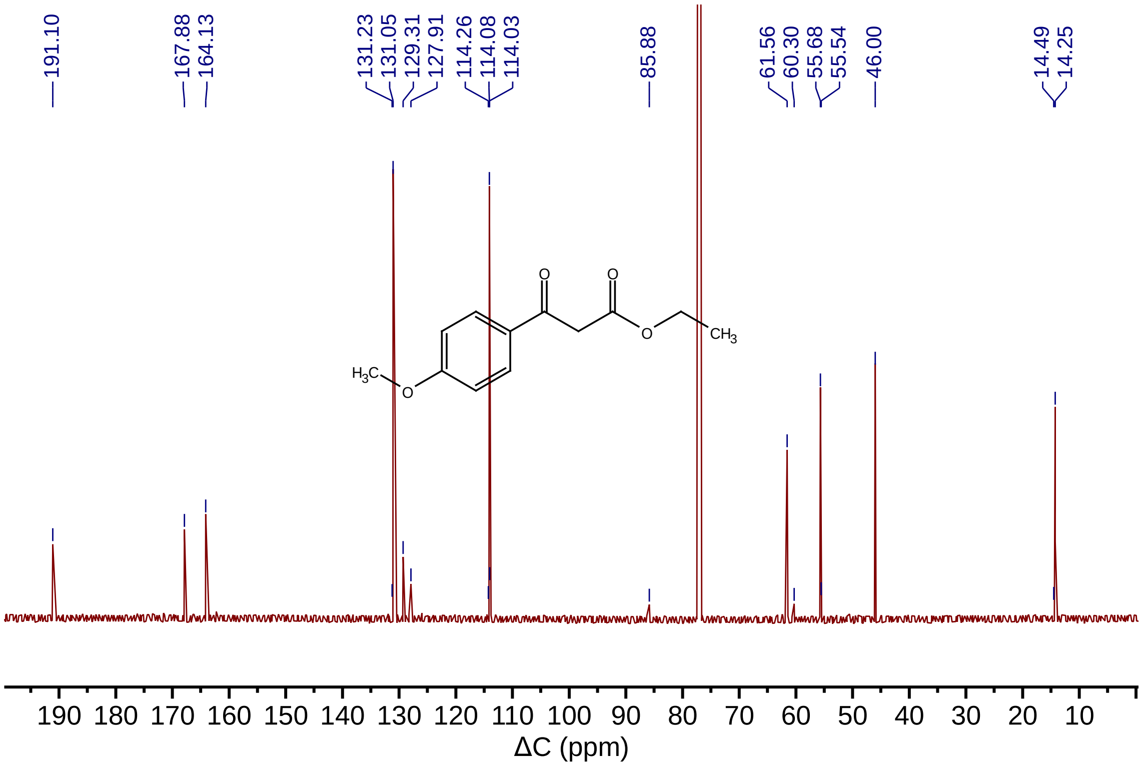

## 2.17 Ethyl (4-chloro)benzoylacetate (4c)

$^1$H NMR (700 MHz, $CDCl_3$)

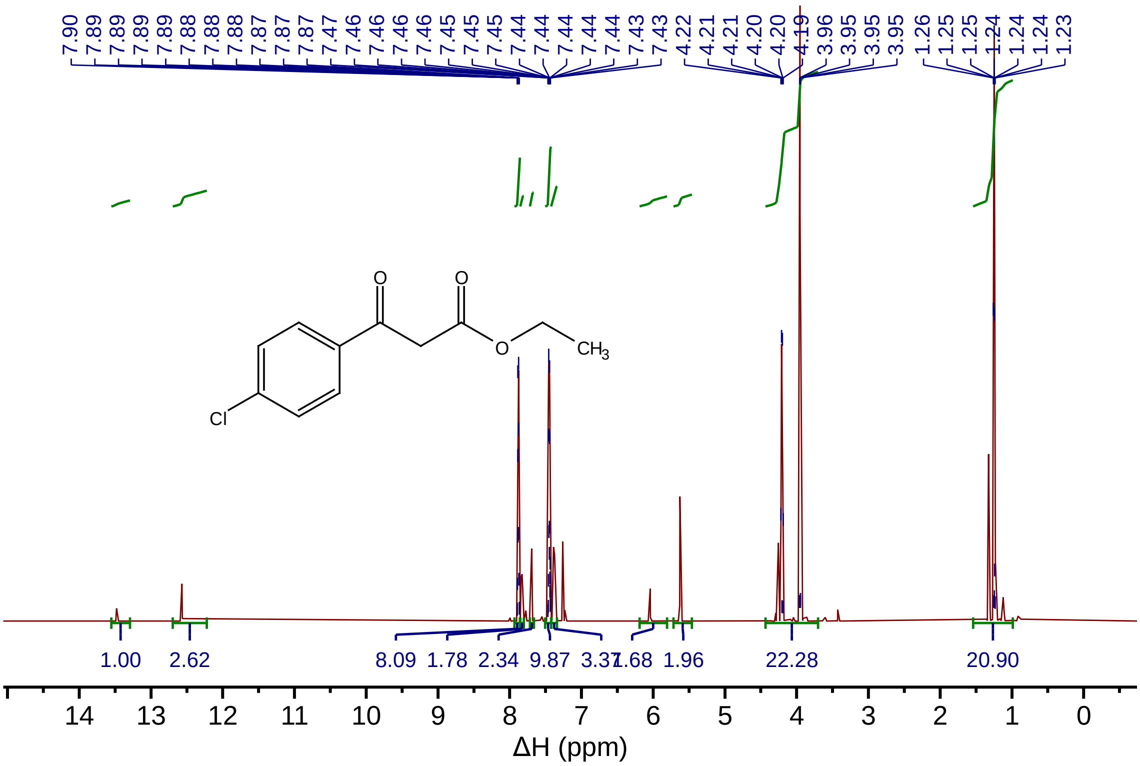


$^{13}$C{$^1$H} NMR (176 MHz, $CDCl_3$)

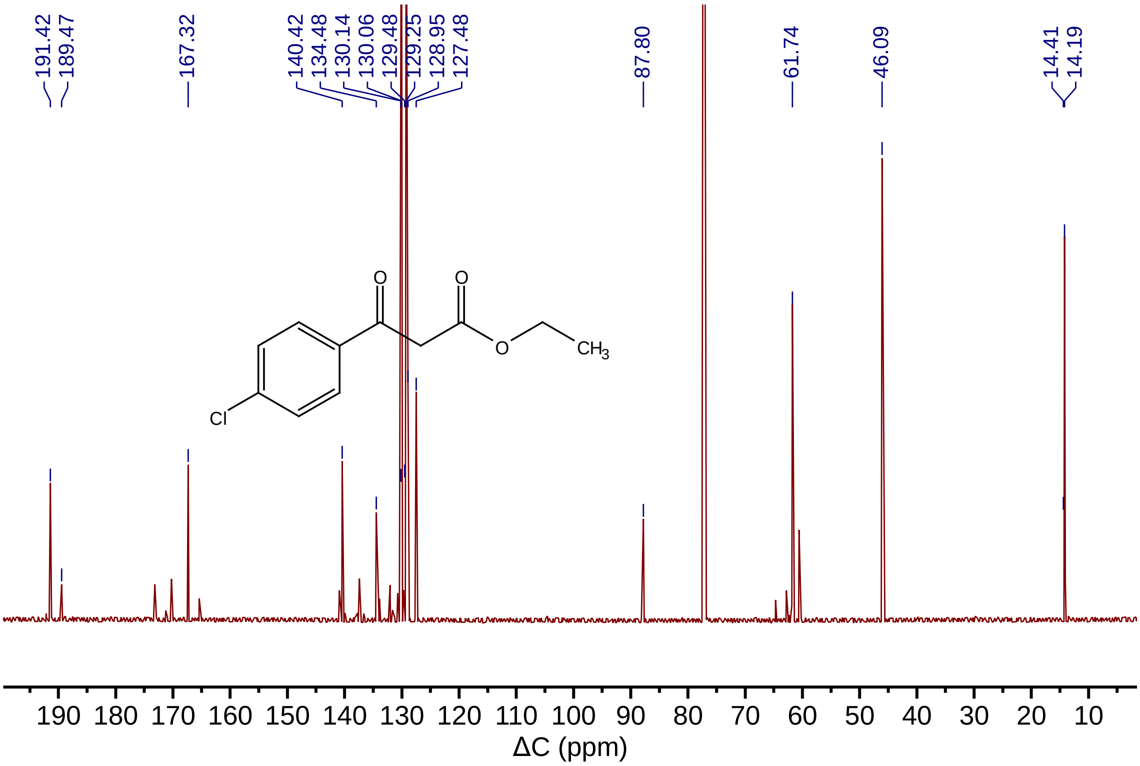

### 2.18 Ethyl (4-trifluoromethyl)benzoylacetate (4d)

$^{1}H$ NMR (700 MHz, $CDCl_3$)

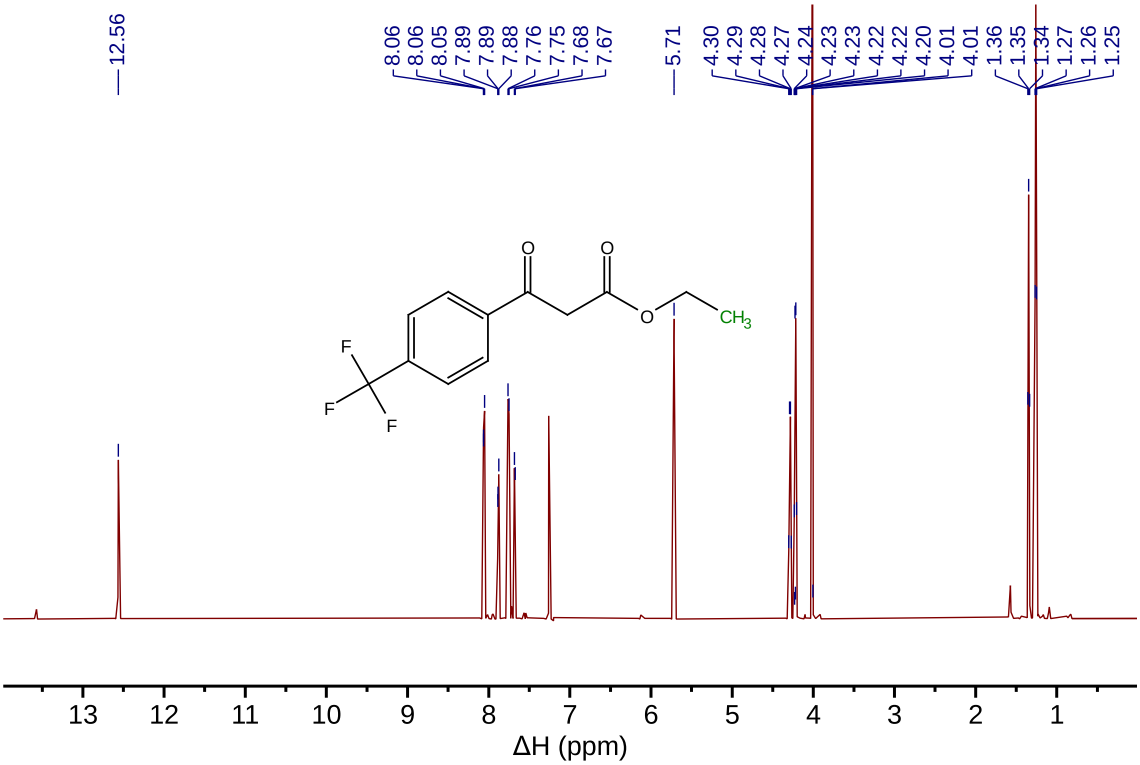


$^{19}F$ NMR (376 MHz, $CDCl_3$)

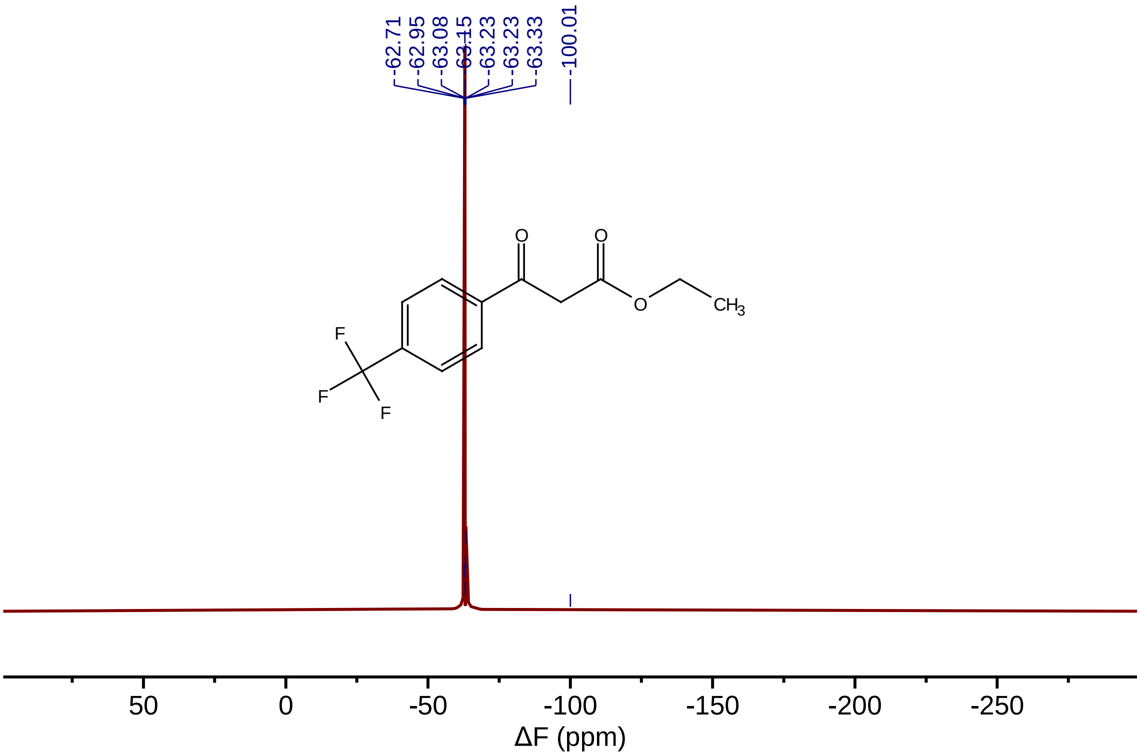

$^{13}C\{^1H\}$ NMR (176 MHz, $CDCl_3$)

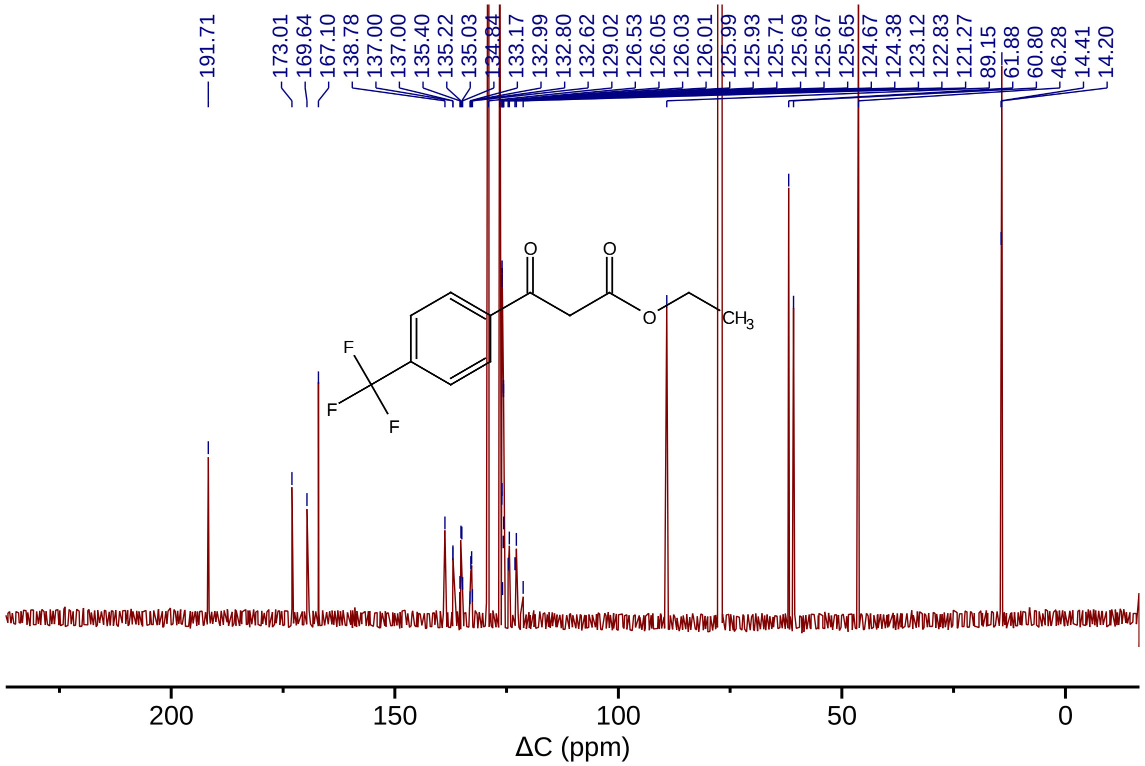

## 2.19 Ethyl (4-nitro)benzoylacetate (4e)

$^1$H NMR (700 MHz, CDCl$_3$)

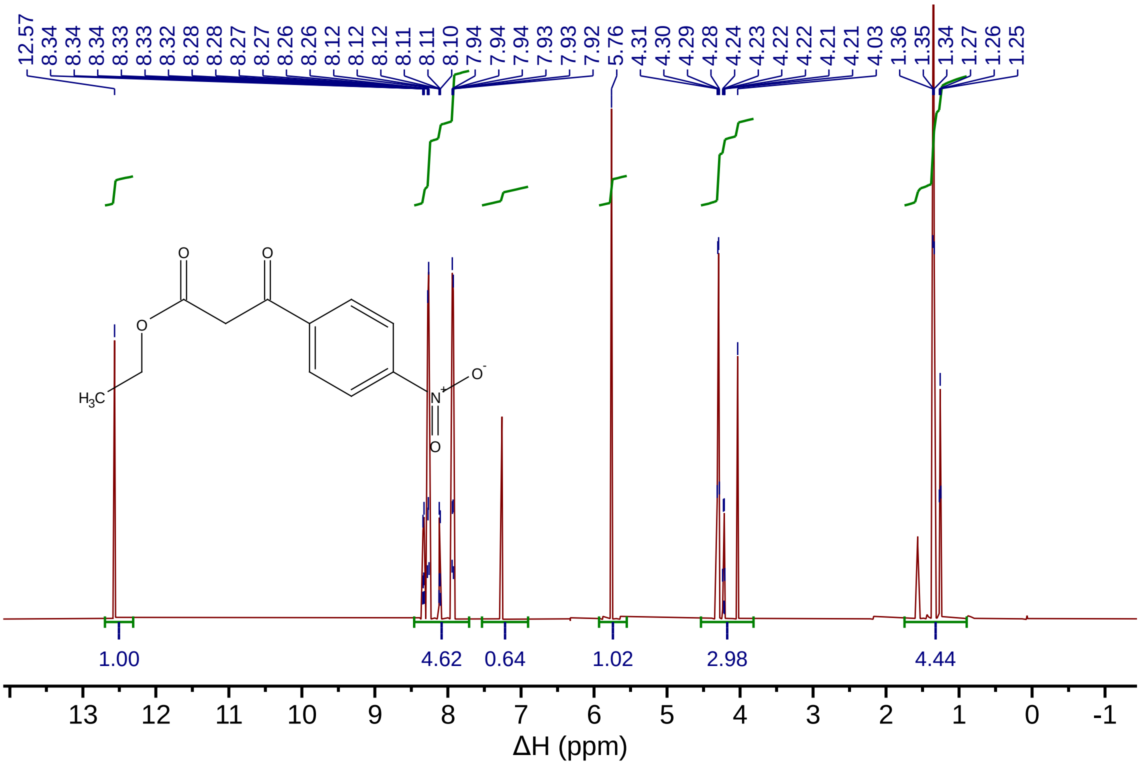

$^{13}$C{$^1$H} NMR (176 MHz, CDCl$_3$)

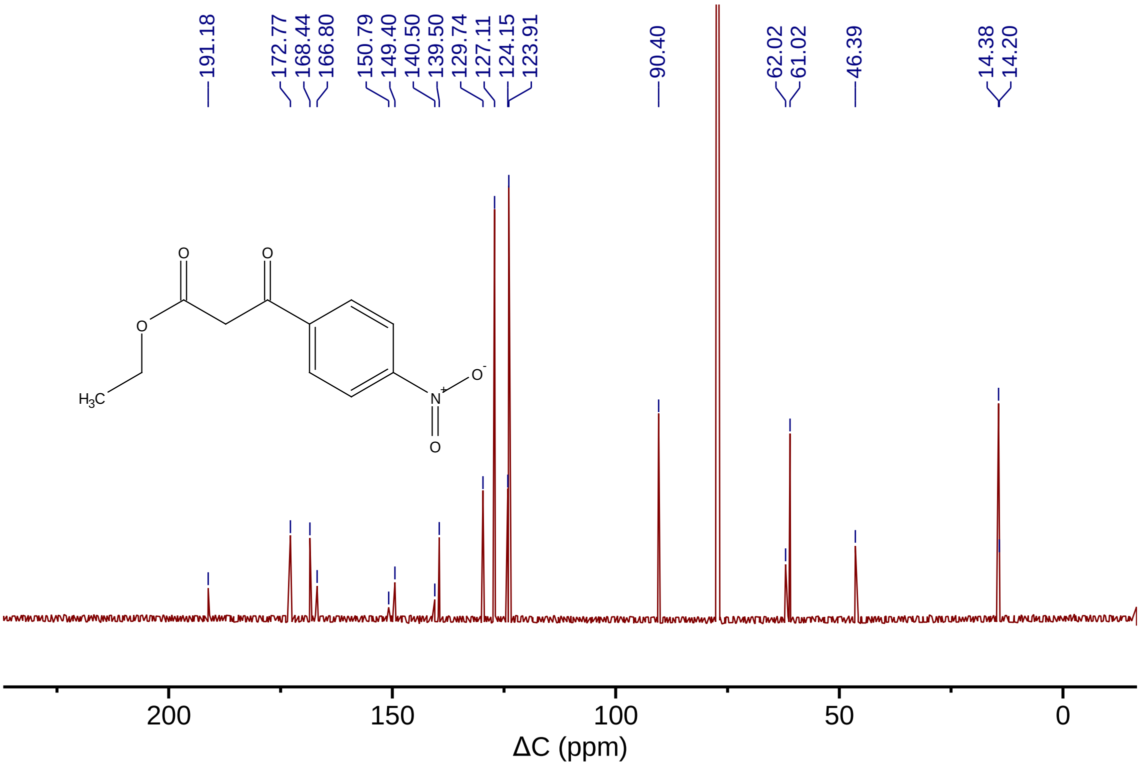

## 2.20 Ethyl (4-cyano)benzoylacetate (4f)

$^{1}H$ NMR (400 MHz, $CDCl_3$)

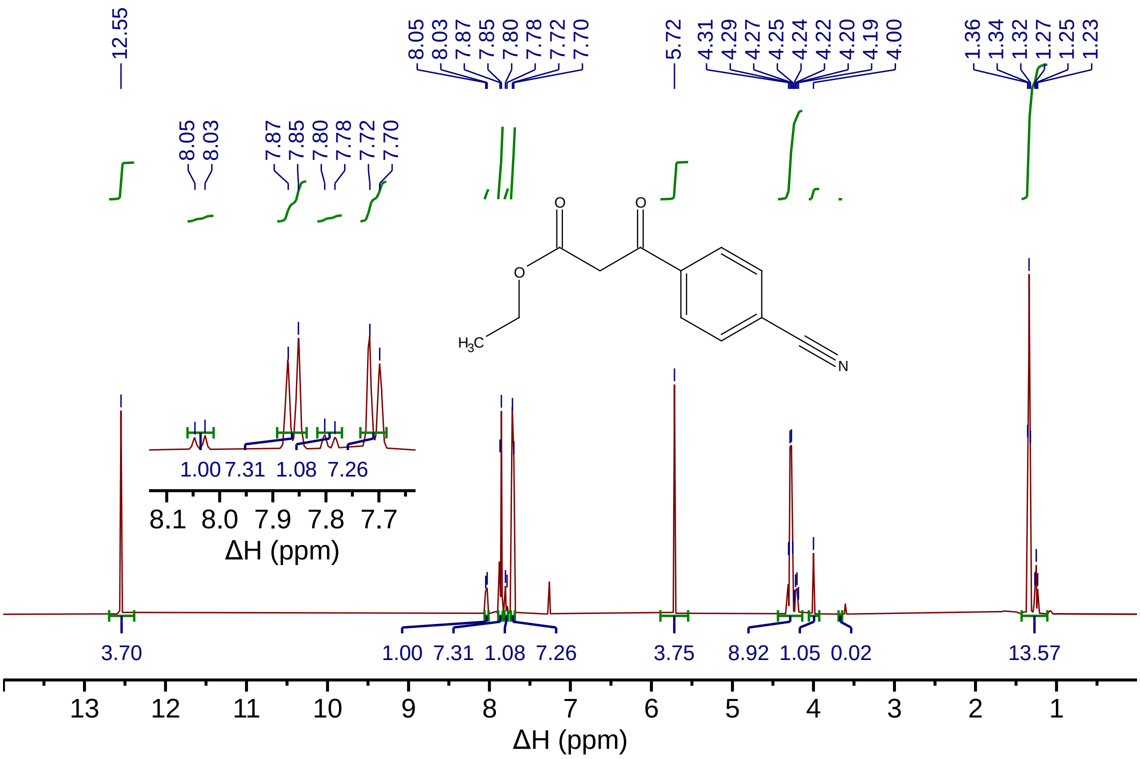

$^{13}C\{^{1}H\}$ NMR (101 MHz, $CDCl_3$)

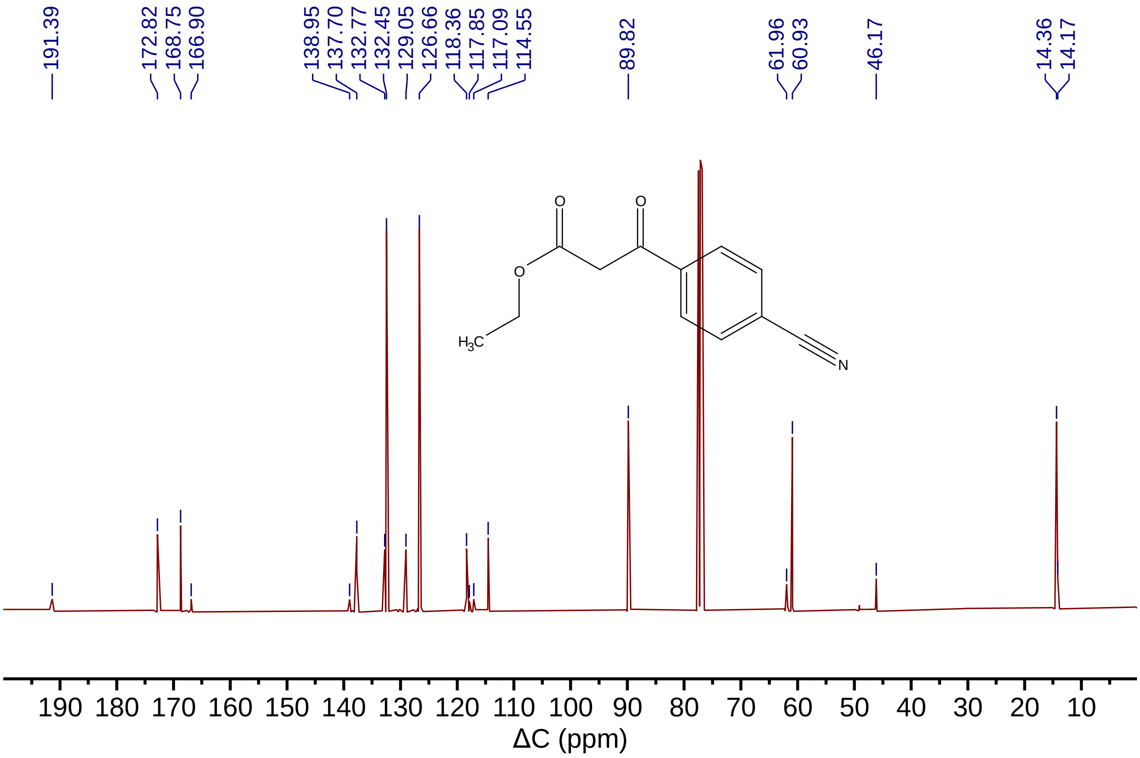

## 2.21 Ethyl (3-nitro)benzoylacetate (4g)

$^{1}H$ NMR (700 MHz, $CDCl_3$)

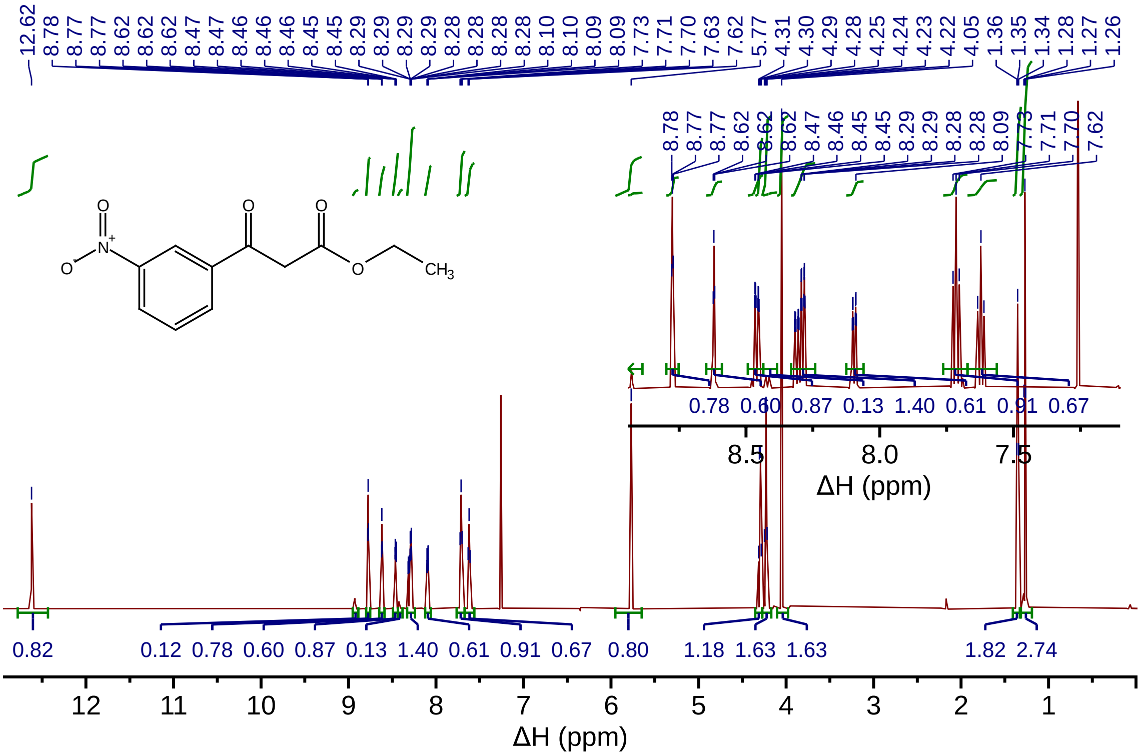

$^{13}C\{^{1}H\}$ NMR (176 MHz, $CDCl_3$)

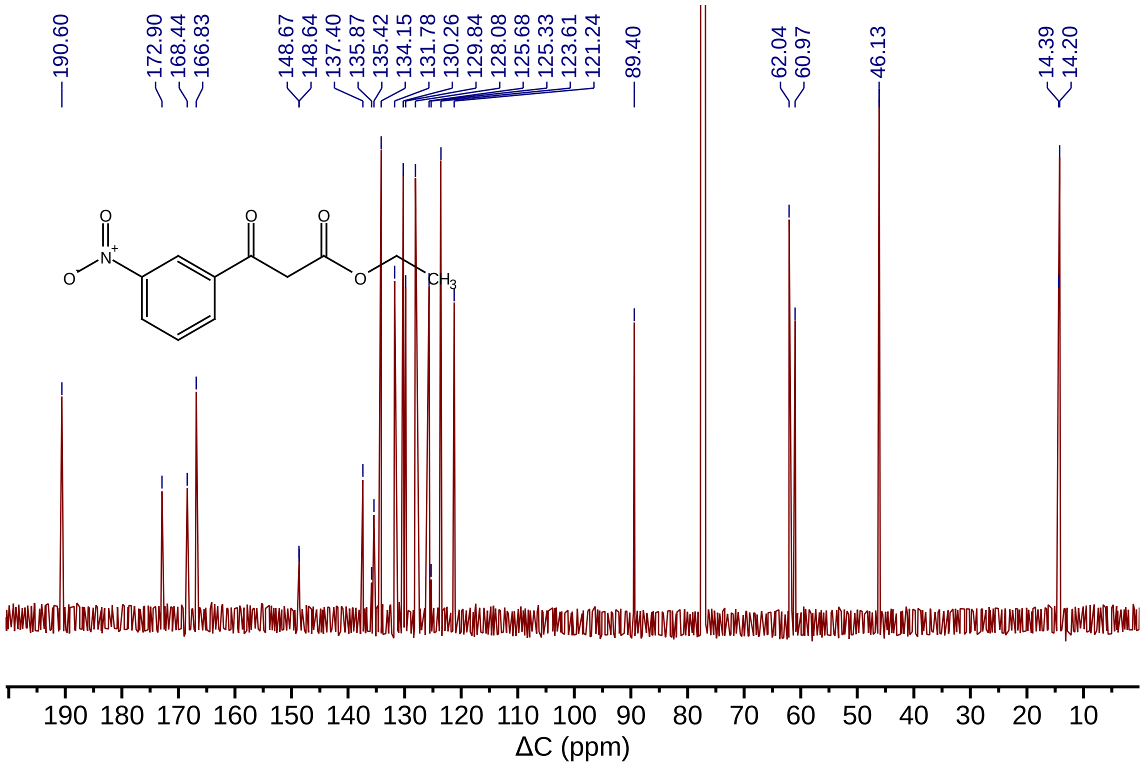

## 2.22 Ethyl 3-oxo-3-(pyridin-4-yl)propanoate (4h)

$^1$H NMR (600 MHz, $CDCl_3$)

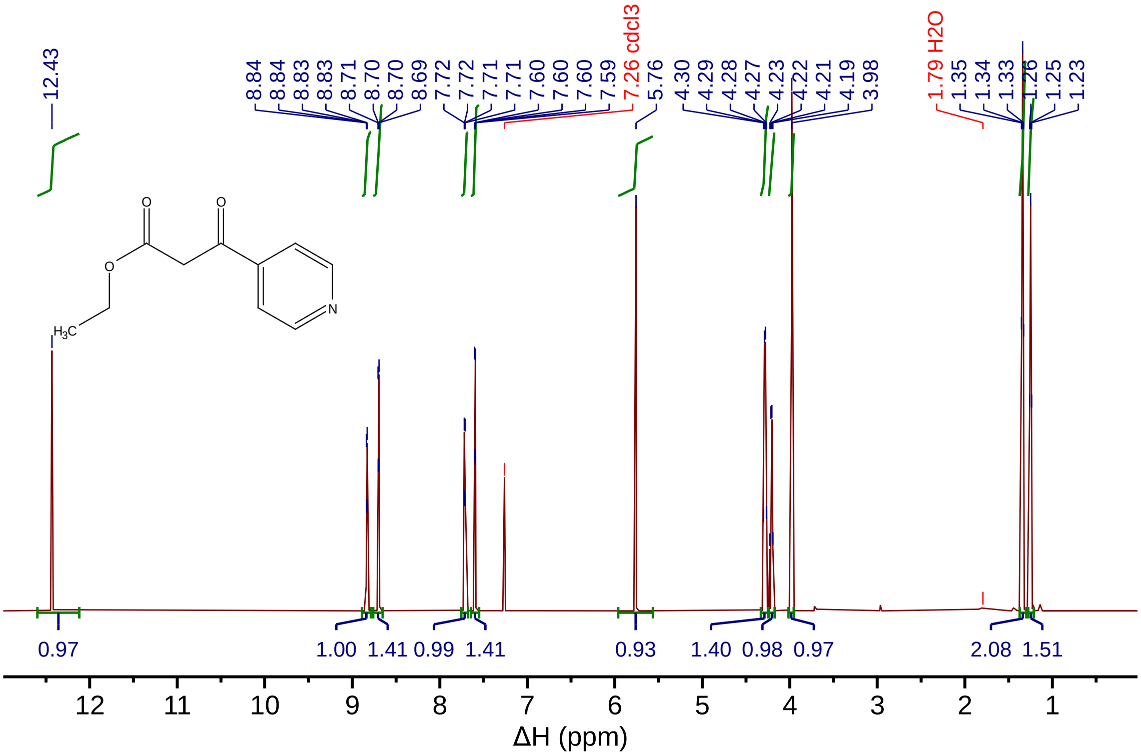


$^{13}C\{^1H\}$ NMR (151 MHz, $CDCl_3$)

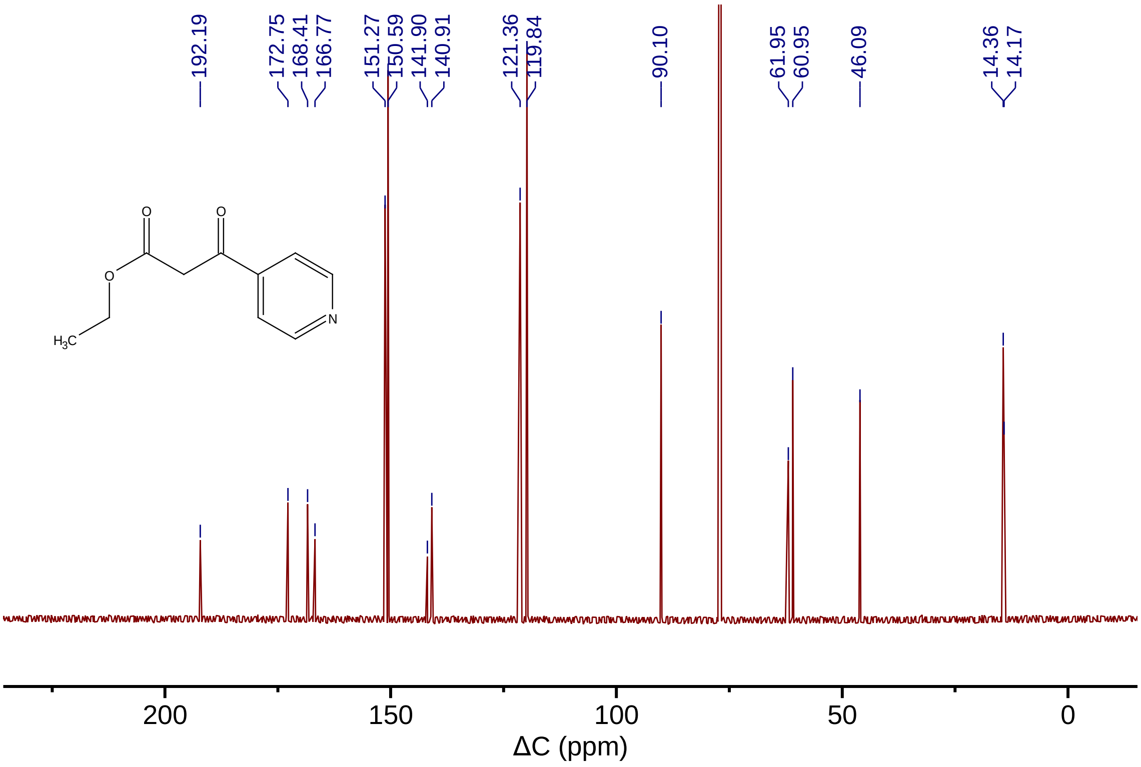

### 2.23 Ethyl 2,2-difluoro-3-oxo-3-phenylpropanoate (5a)

$^1H$ NMR (700 MHz, $CDCl_3$)

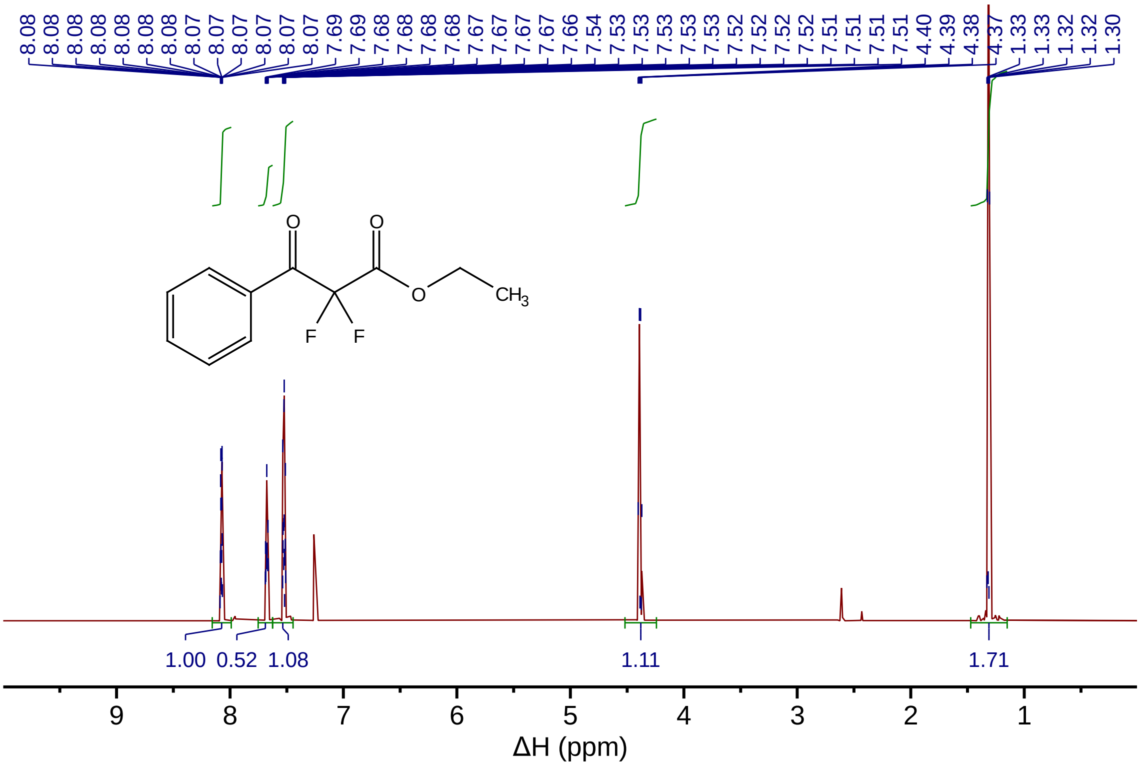

$^{19}F$ NMR (376 MHz, $CDCl_3$)

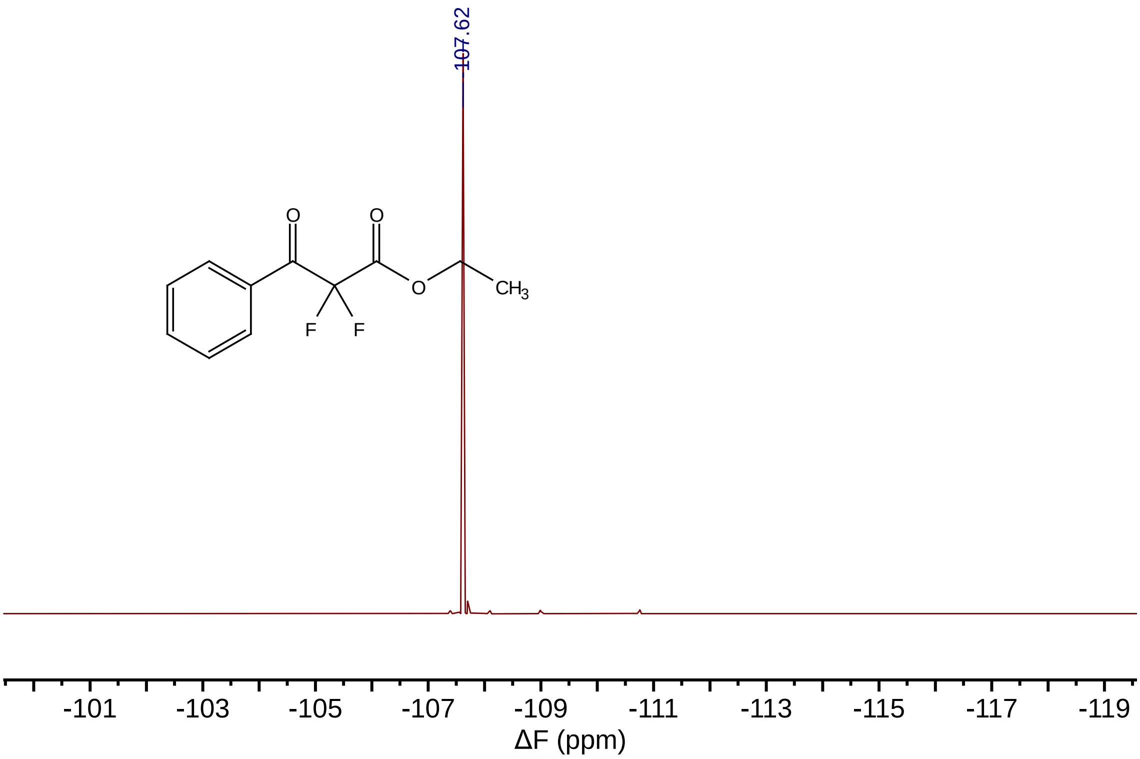

$^{13}C\{^1H\}$ NMR (176 MHz, $CDCl_3$)

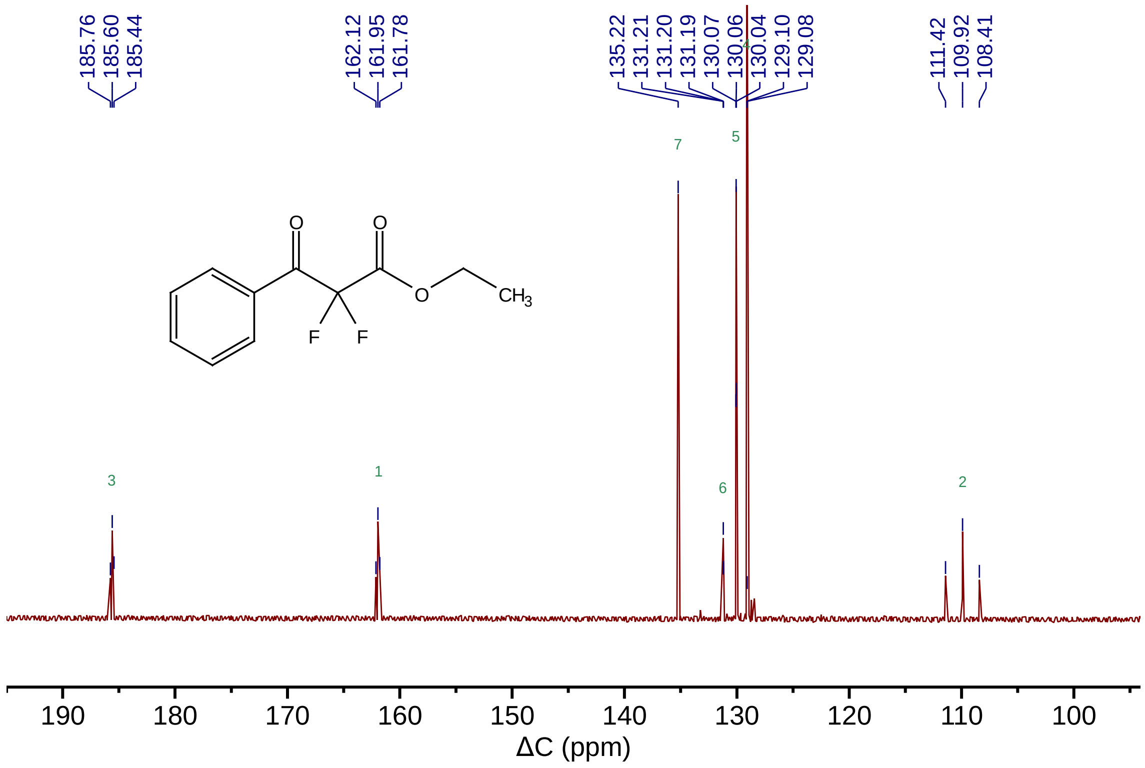

## 2.24 Ethyl 2,2-difluoro-3-(4-chlorophenyl)-3-oxopropanoate (5c)

$^1$H NMR (600 MHz, $CDCl_3$)

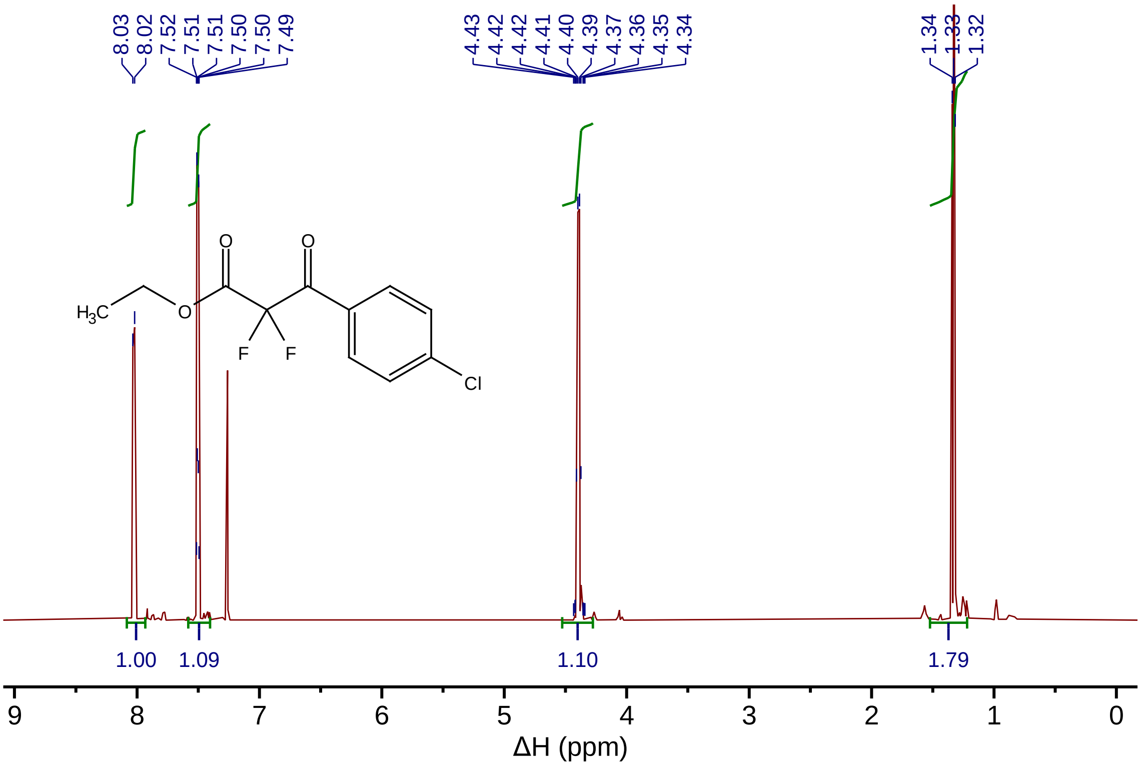


$^{19}$F NMR (376 MHz, $CDCl_3$)

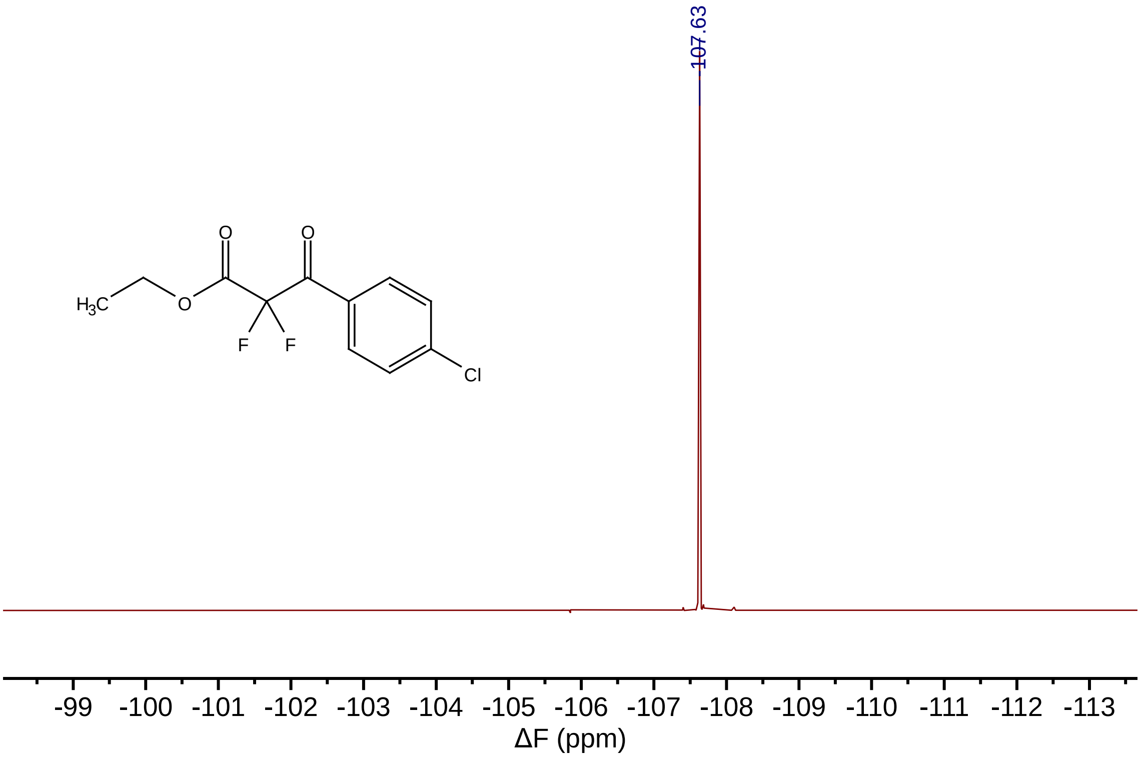

$^{13}C\{^{1}H\}$ NMR (151 MHz, $CDCl_3$)

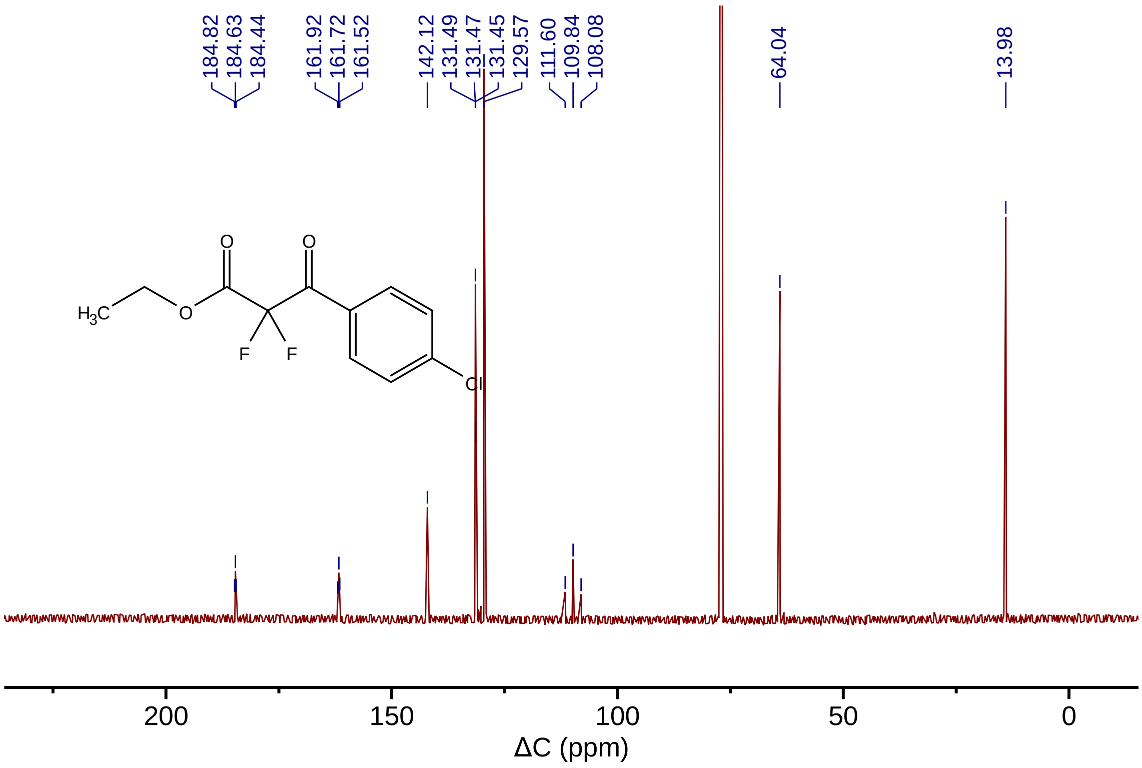

## 2.25 Ethyl 2,2-difluoro-3-(4-trifluoromethylphenyl)-3-oxopropanoate (5d)

$^{1}H$ NMR (700 MHz, $CDCl_3$)

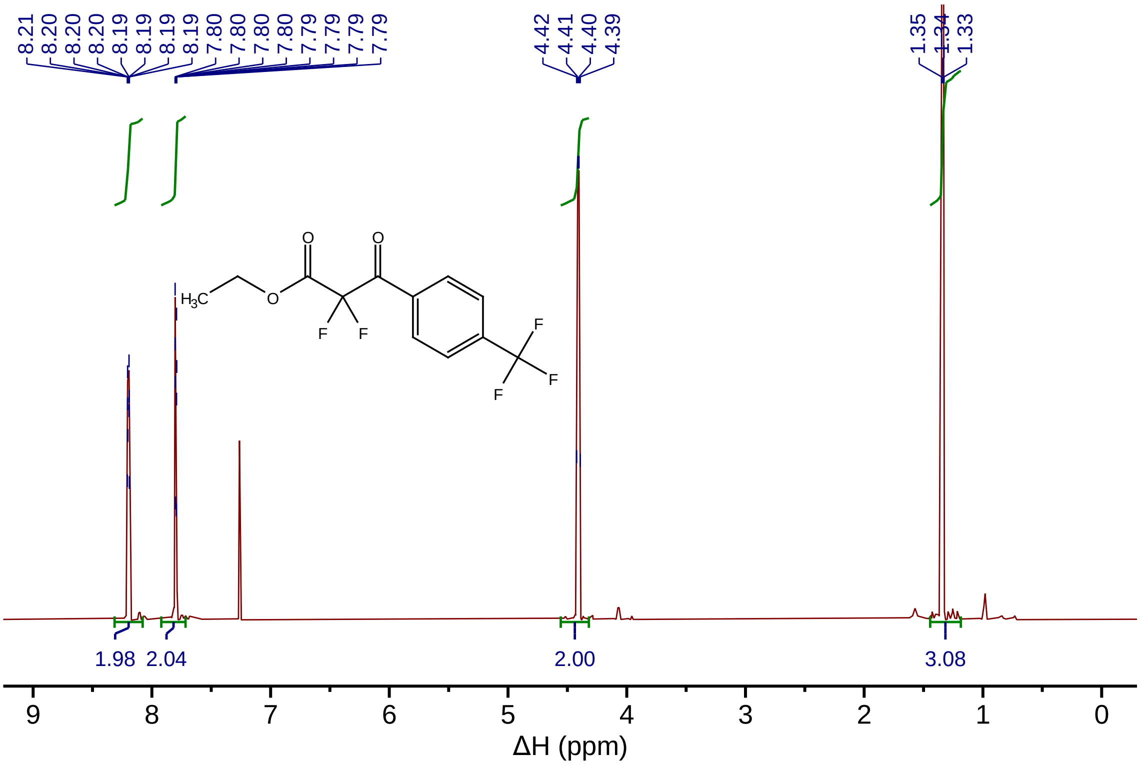


$^{19}F$ NMR (376 MHz, $CDCl_3$)

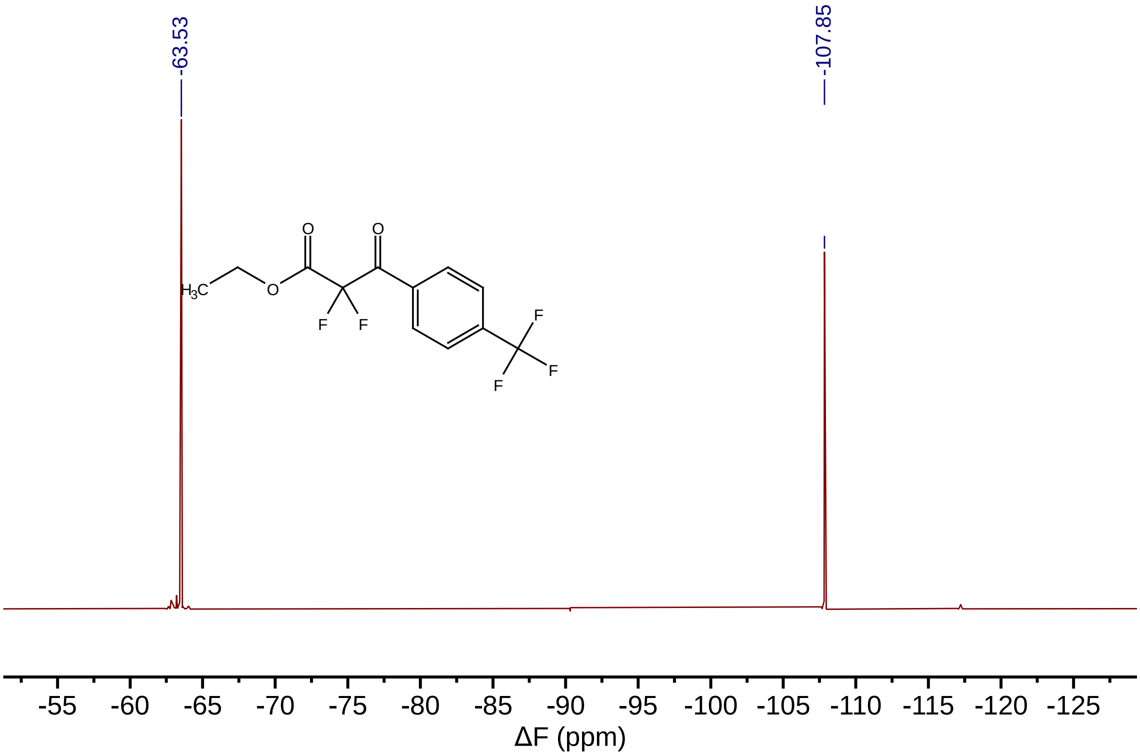

$^{13}C\{^{1}H\}$ NMR (176 MHz, $CDCl_3$)

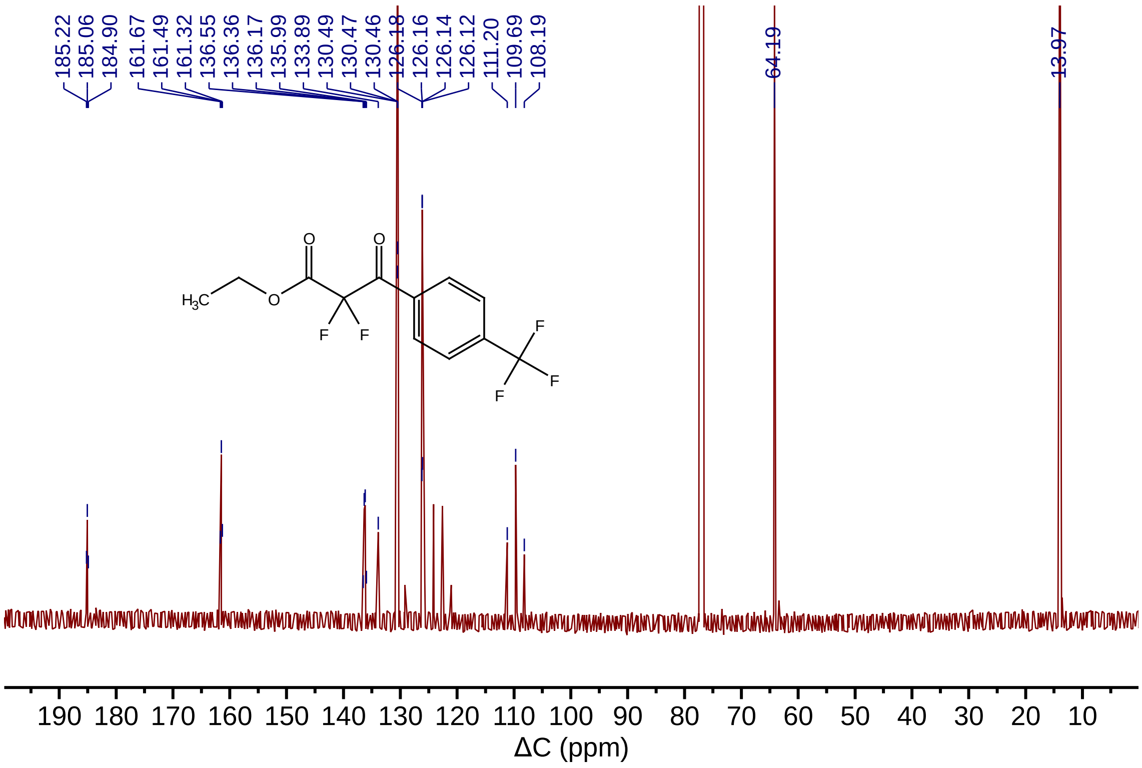

## 2.26 Ethyl 2,2-difluoro-3-(4-nitrophenyl)-3-oxopropanoate (5e)

$^{1}$H NMR (700 MHz, $CDCl_3$)

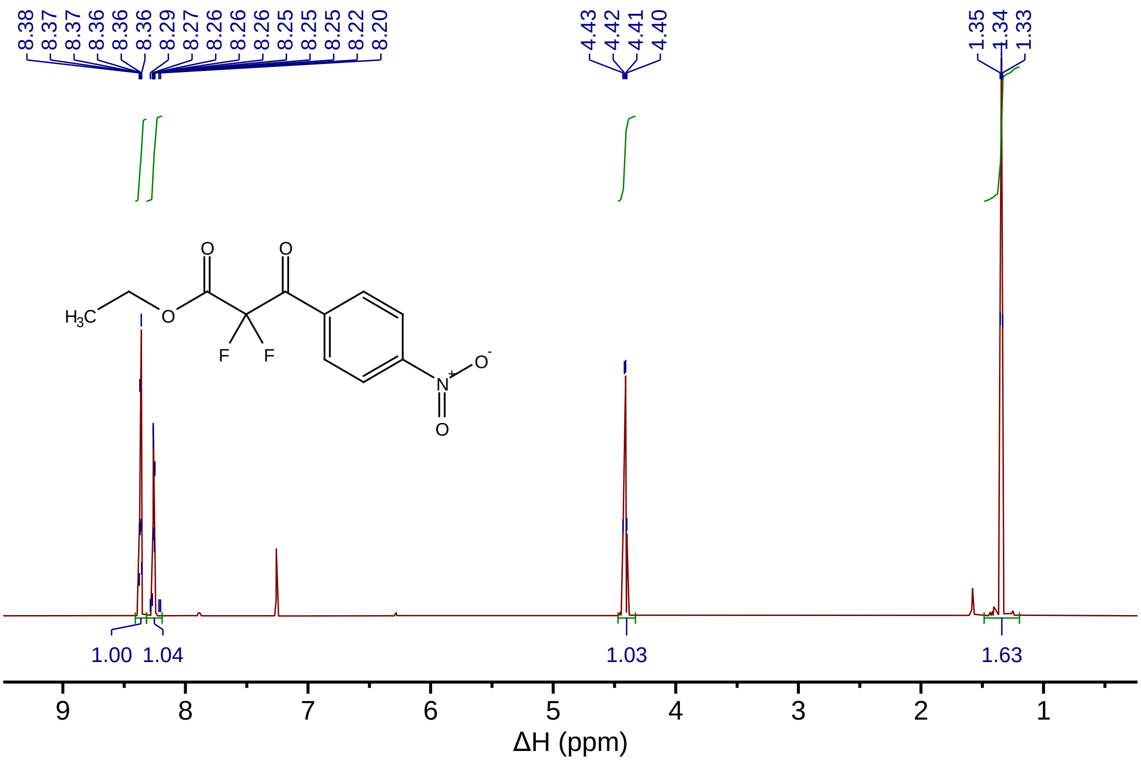


$^{19}$F NMR (376 MHz, $CDCl_3$)

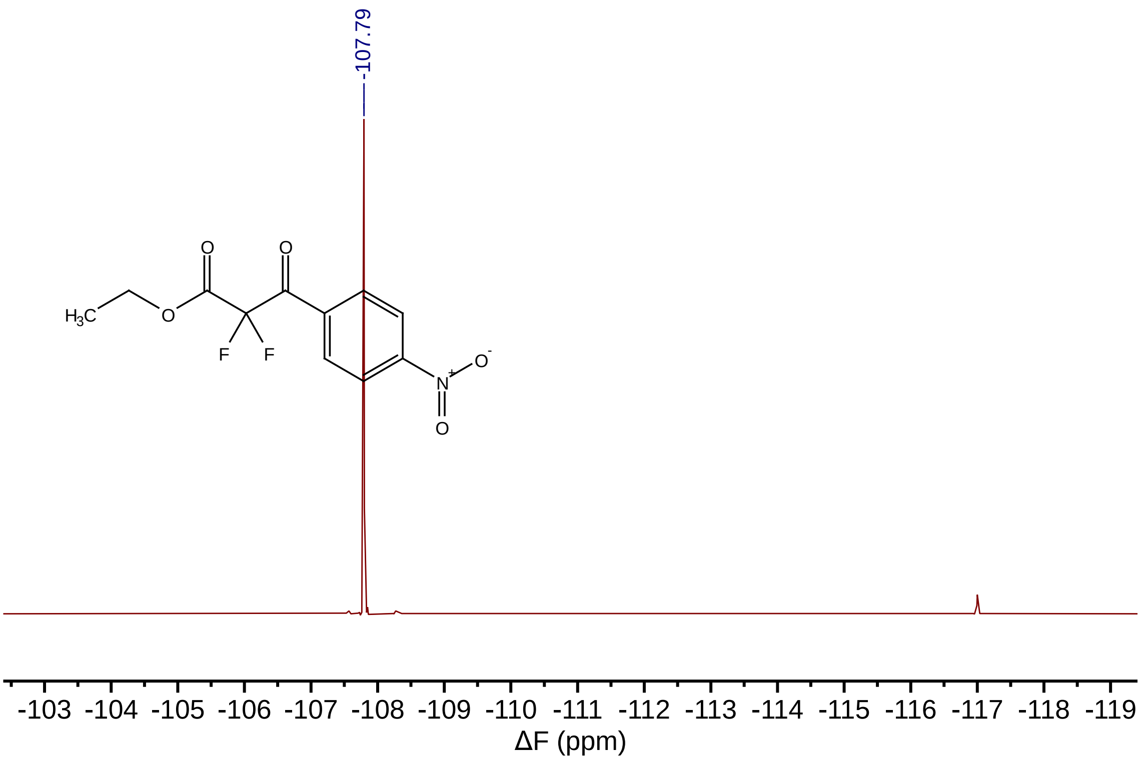

$^{13}C\{^{1}H\}$ NMR (176 MHz, $CDCl_3$)

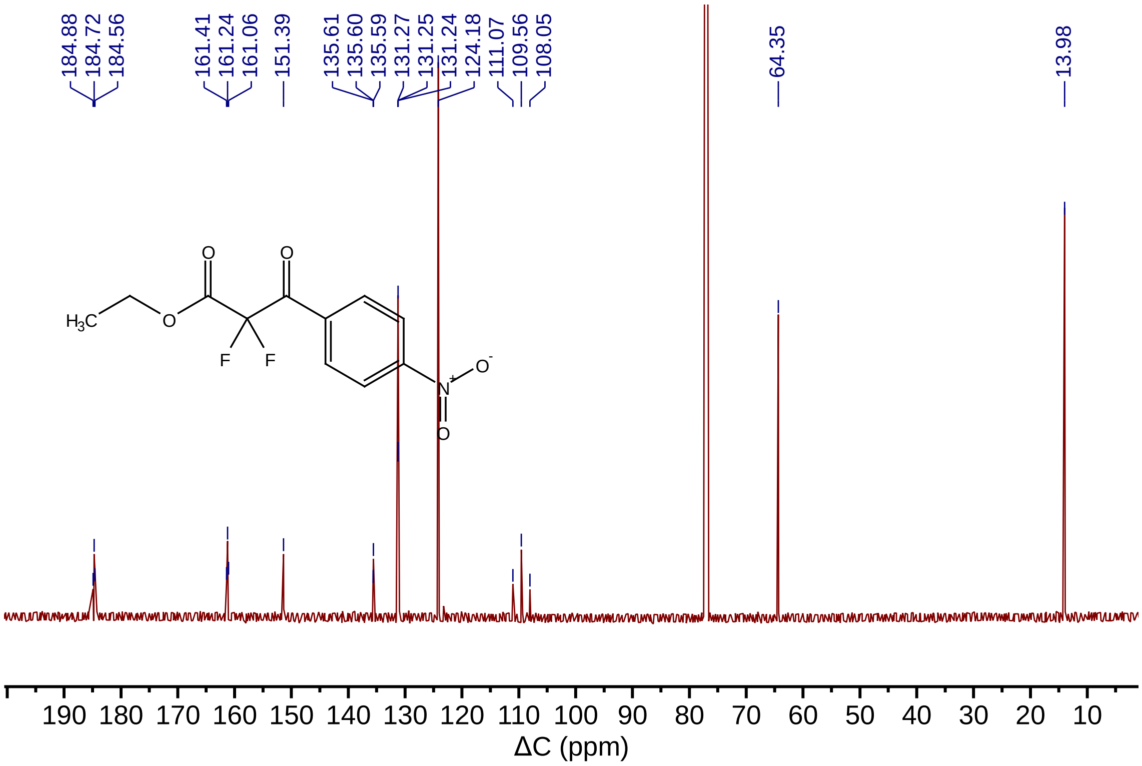

## 2.27 Ethyl 2,2-difluoro-3-(4-cyanophenyl)-3-oxopropanoate (5f)

$^1H$ NMR (400 MHz, $CDCl_3$)

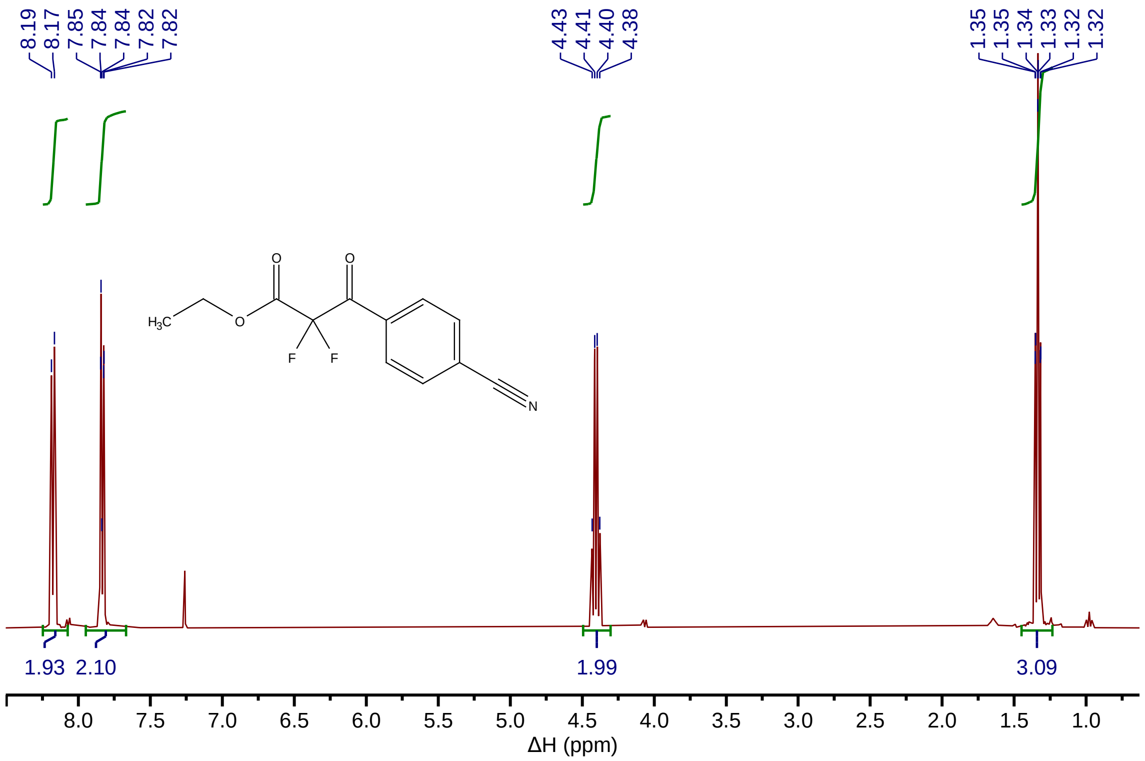


$^{19}F$ NMR (376 MHz, $CDCl_3$)

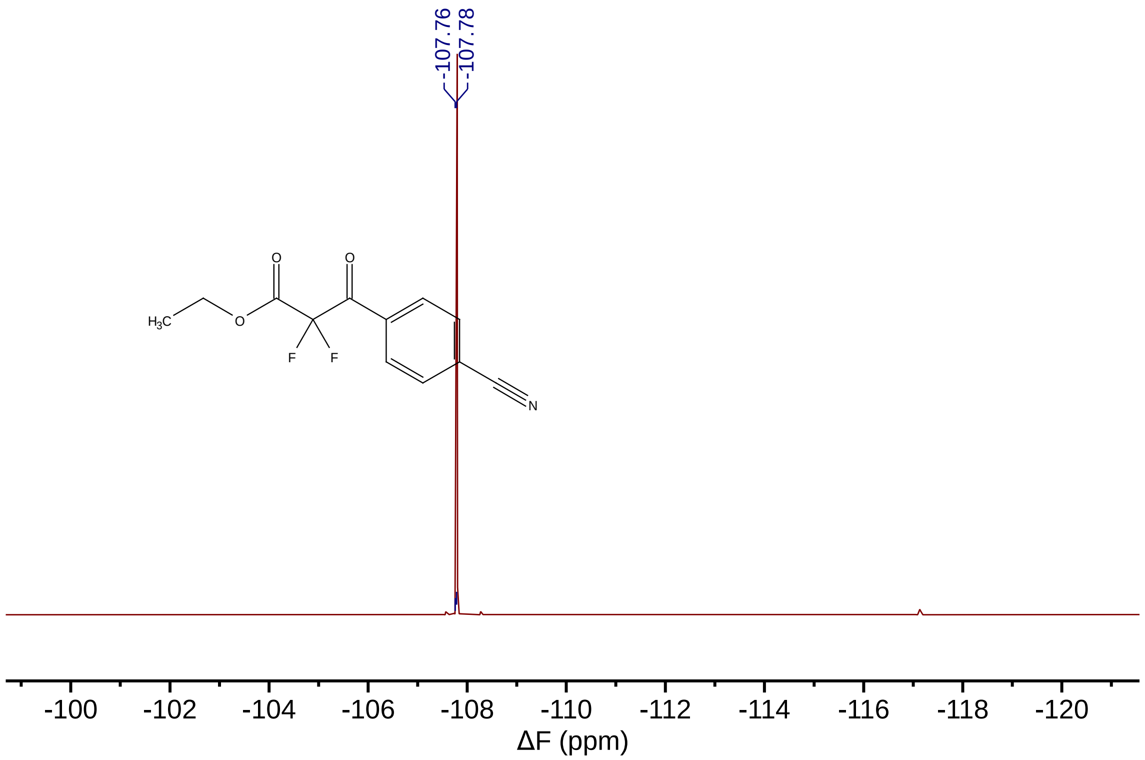

$^{13}C\{^{1}H\}$ NMR (101 MHz, $CDCl_3$)

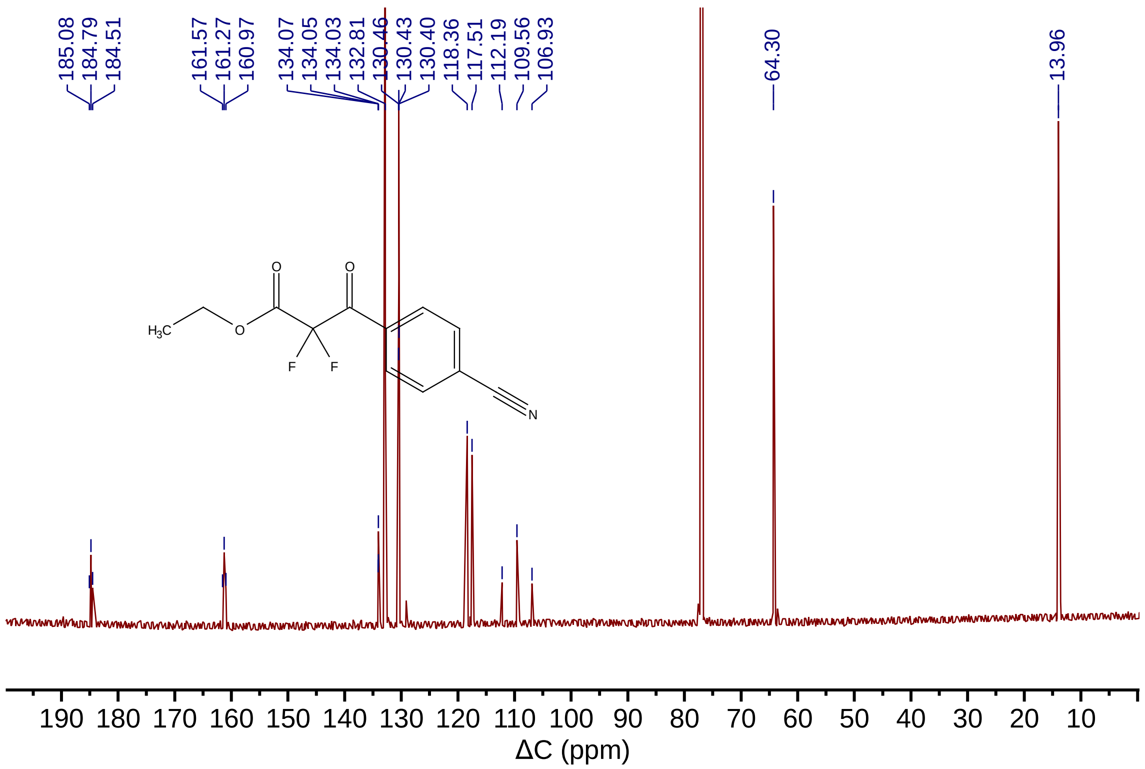

## 2.28 Ethyl 2,2-difluoro-3-(3-nitrophenyl)-3-oxopropanoate (5g)

$^{1}H$ NMR (700 MHz, $CDCl_3$)

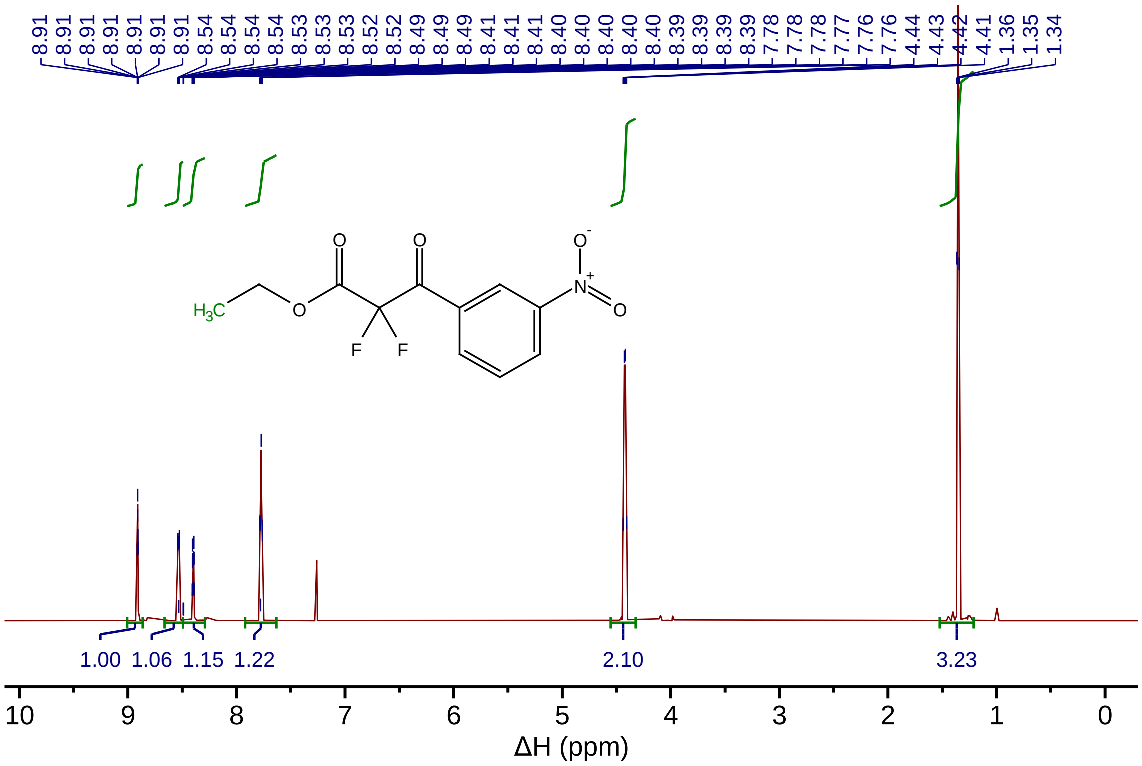

$^{19}F$ NMR (376 MHz, $CDCl_3$)

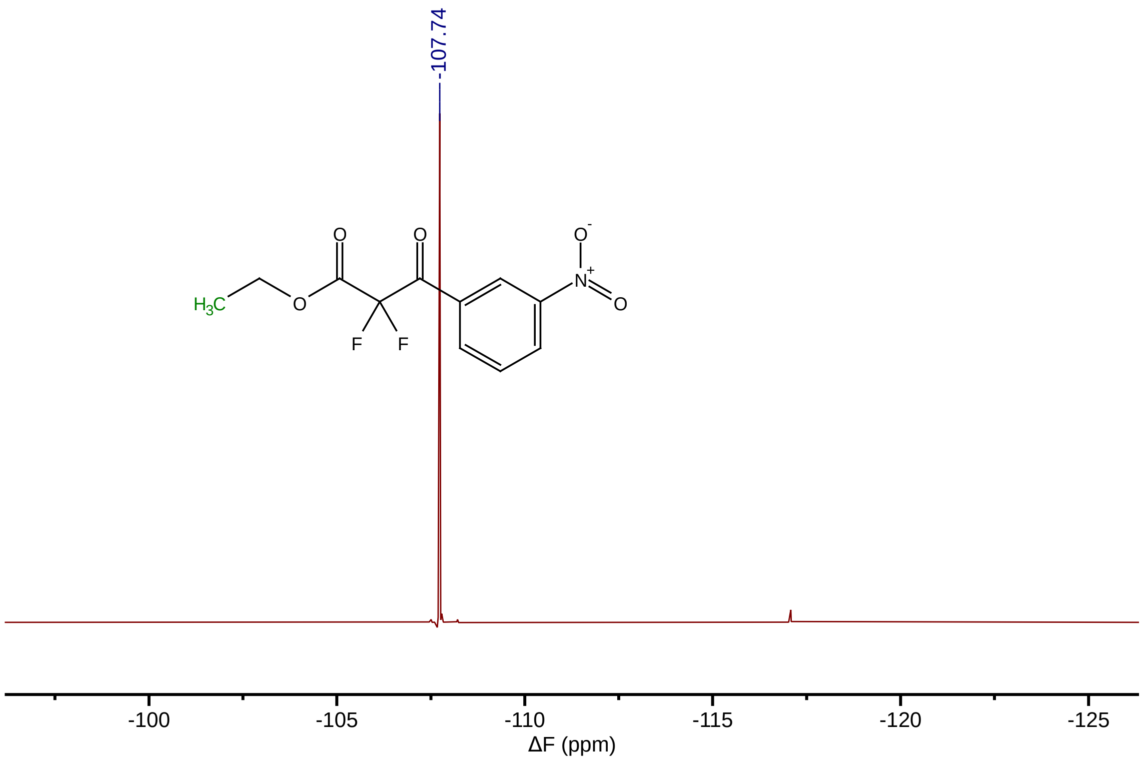

$^{13}C\{^{1}H\}$ NMR (176 MHz, $CDCl_3$)

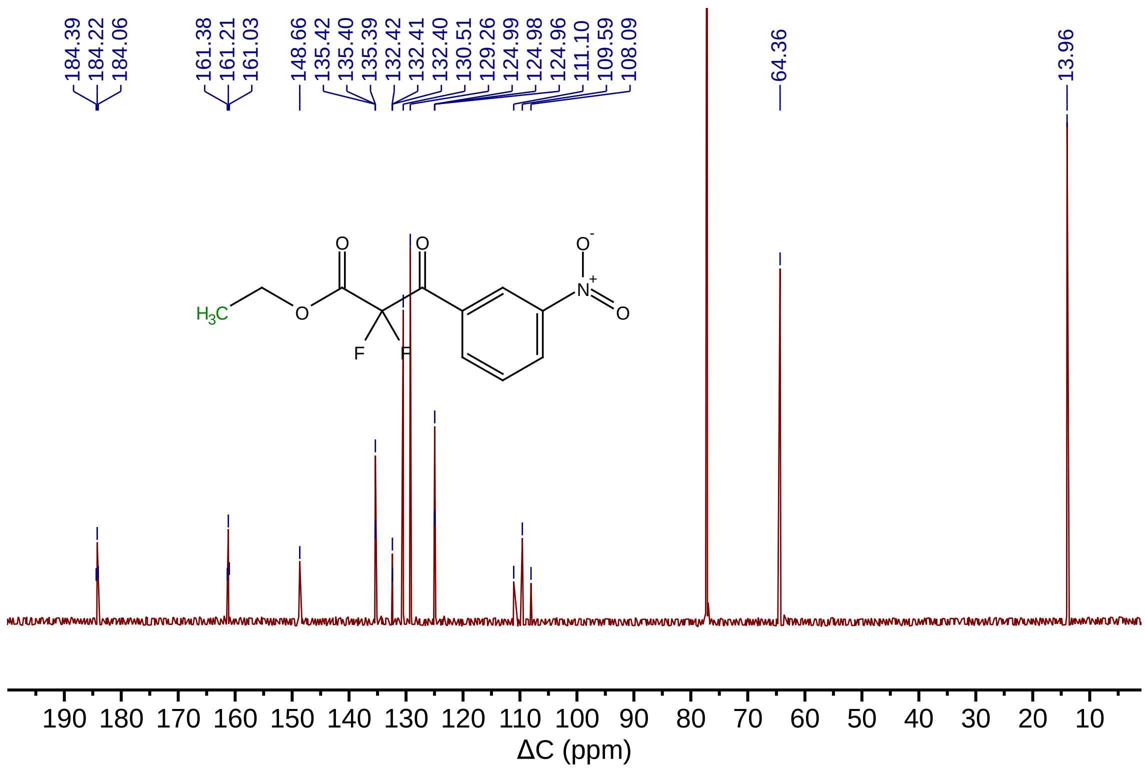

# 3 Single crystal X-ray crystallography

The X-ray single crystal data have been collected at temperature 120.0(2)K using Mo Kα radiation (λ =0.71073Å) on Bruker D8Venture (Photon100 CMOS detector, IμS-microsource, focusing mirrors, compounds **1hA**, **1hB**, **3a**, **3f**, **4c**, **4g** and **5e**) and Agilent XCalibur (compound **3i**, Sapphire-3 CCD detector, fine-focus sealed tube, graphite monochromator) diffractometers equipped with Cryostream (Oxford Cryosystems) open-flow nitrogen cryostats. All structures were solved by direct method and refined by full-matrix least squares on $F^2$ for all data using Olex2[21] and SHELXTL[22] software. All non-hydrogen atoms were refined in anisotropic approximation, hydrogen atoms in structures **1hA, 1hB, 4c** and **5e** were refined isotropically, the hydrogen atoms in other structures were placed in the calculated positions and refined in riding mode. Crystal data and parameters of refinement for **1hA**, **1hB**, **3a**, **3i**, **3f**, **4c**, **4g** and **5e** are listed in sections **3.1** to **3.8**, respectively, below. Crystallographic data for the structure have been deposited with the Cambridge Crystallographic Data Centre, CCDC-2288841-2288848.

### 3.1 Crystallographic data for 1h (polymorph A)

1,3-Bis(4-fluorophenyl)-3-phenylpropane-1,3-dione (enol) **1h**

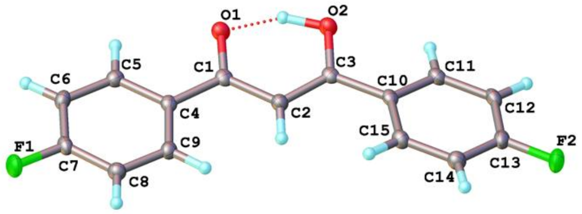


Crystal data and structure refinement for **1h**

| | |
|---|---|
| CCDC | 2288841 |
| Empirical formula | $C_{15}H_{10}F_2O_2$ |
| Formula weight | 260.23 |
| Temperature/K | 120.0 |
| Crystal system | monoclinic |
| Space group | $P2_1/c$ |
| a/ Å | 11.8429(6) |
| b/ Å | 8.9668(4) |
| c/ Å | 10.9377(5) |
| $\alpha$/° | 90 |
| $\beta$/° | 97.383(2) |
| $\gamma$/° | 90 |
| Volume/ Å$^3$ | 1151.88(9) |
| Z | 4 |
| $\rho_{calc}$g/cm$^3$ | 1.501 |
| μ/mm$^{-1}$ | 0.120 |
| F(000) | 536.0 |
| Crystal size/mm$^3$ | 0.41 × 0.23 × 0.08 |
| Radiation | MoK$\alpha$ ($\lambda$ = 0.71073) |
| 2Θ range for data collection/° | 5.716 to 57.99 |
| Index ranges | $-16 \le h \le 16$, $-12 \le k \le 12$, $-14 \le l \le 14$ |
| Reflections collected | 17075 |
| Independent reflections | 3054 [$R_{int}$ = 0.0620, $R_{sigma}$ = 0.0563] |
| Data/restraints/parameters | 3054/0/213 |
| Goodness-of-fit on F$^2$ | 1.048 |
| Final R indexes [I>=2$\sigma$ (I)] | $R_1$ = 0.0394, w$R_2$ = 0.0986 |
| Final R indexes [all data] | $R_1$ = 0.0528, w$R_2$ = 0.1055 |
| Largest diff. peak/hole [e/Å$^3$] | 0.32/-0.24 |

### 3.2 Crystallographic data for 1h (polymorph B)

1,3-Bis(4-fluorophenyl)-3-phenylpropane-1,3-dione (enol) **1h**

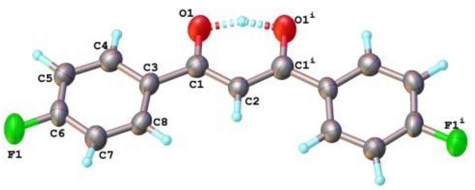


Crystal data and structure refinement for **1hB**

| | |
|---|---|
| CCDC | 2288842 |
| Empirical formula | $C_{15}H_{10}F_2O_2$ |
| Formula weight | 260.23 |
| Temperature/K | 120.0 |
| Crystal system | monoclinic |
| Space group | C2/c |
| a/ Å | 3.8574(15) |
| b/ Å | 11.914(5) |
| c/ Å | 25.388(10) |
| $\alpha$/° | 90 |
| $\beta$/° | 94.36 |
| $\gamma$/° | 90 |
| Volume/ $Å^3$ | 1163.4(8) |
| Z | 4 |
| $\rho_{calc}g/cm^3$ | 1.486 |
| $\mu/mm^{-1}$ | 0.119 |
| F(000) | 536.0 |
| Crystal size/$mm^3$ | 0.28 × 0.24 × 0.01 |
| Radiation | MoK$\alpha$ ($\lambda$ = 0.71073) |
| 2Θ range for data collection/° | 4.828 to 53.952 |
| Index ranges | −4 ≤ h ≤ 4, −14 ≤ k ≤ 14, −32 ≤ l ≤ 32 |
| Reflections collected | 7253 |
| Independent reflections | 1257 [$R_{int}$ = 0.1021, $R_{sigma}$ = 0.0998] |
| Data/restraints/parameters | 1257/0/108 |
| Goodness-of-fit on $F^2$ | 0.951 |
| Final R indexes [I>=2$\sigma$ (I)] | $R_1$ = 0.0553, $wR_2$ = 0.1169 |
| Final R indexes [all data] | $R_1$ = 0.1410, $wR_2$ = 0.1505 |
| Largest diff. peak/hole [e/$Å^3$] | 0.16/−0.27 |

### 3.3 Crystallographic data for 3a

2,2-Difluoro-1,3-diphenylpropane-1,3-dione **3a**

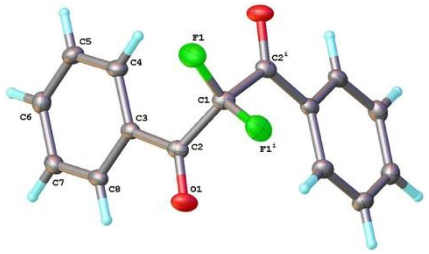


Crystal data and structure refinement for **3a**

| | |
|---|---|
| CCDC | 2288843 |
| Empirical formula | $C_{15}H_{10}F_2O_2$ |
| Formula weight | 260.23 |
| Temperature/K | 120.0 |
| Crystal system | monoclinic |
| Space group | C2/c |
| a/ Å | 19.9091(14) |
| b/ Å | 5.3345(4) |
| c/ Å | 13.0943(9) |
| $\alpha$/° | 90 |
| $\beta$/° | 123.898(2) |
| $\gamma$/° | 90 |
| Volume/ Å$^3$ | 1154.31(15) |
| Z | 4 |
| $\rho_{calc}$g/cm$^3$ | 1.497 |
| μ/mm$^{-1}$ | 0.120 |
| F(000) | 536.0 |
| Crystal size/mm$^3$ | 0.31 × 0.3 × 0.18 |
| Radiation | MoK$\alpha$ ($\lambda$ = 0.71073) |
| 2Θ range for data collection/° | 4.93 to 57.924 |
| Index ranges | −26 ≤ h ≤ 26, −7 ≤ k ≤ 7, −17 ≤ l ≤ 17 |
| Reflections collected | 11191 |
| Independent reflections | 1531 [$R_{int}$ = 0.0338, $R_{sigma}$ = 0.0206] |
| Data/restraints/parameters | 1531/0/87 |
| Goodness-of-fit on F$^2$ | 1.086 |
| Final R indexes [I>=2$\sigma$ (I)] | $R_1$ = 0.0480, w$R_2$ = 0.1373 |
| Final R indexes [all data] | $R_1$ = 0.0597, w$R_2$ = 0.1480 |
| Largest diff. peak/hole [e/Å$^3$] | 0.45/−0.39 |

## 3.4 Crystallographic data for 3f

2,2-Difluoro-1-(4-nitrophenyl)-3-phenylpropane-1,3-dione **3f**

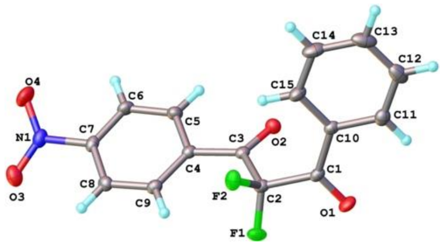


Crystal data and structure refinement for **3f**

| | |
|---|---|
| CCDC | 2288844 |
| Empirical formula | $C_{15}H_9F_2NO_4$ |
| Formula weight | 305.23 |
| Temperature/K | 120.0 |
| Crystal system | triclinic |
| Space group | P-1 |
| a/ Å | 7.1508(3) |
| b/ Å | 8.0322(3) |
| c/ Å | 12.1238(5) |
| $\alpha$/° | 77.7835(16) |
| $\beta$/° | 76.0100(16) |
| $\gamma$/° | 77.3328(17) |
| Volume/ Å$^3$ | 649.93(5) |
| Z | 2 |
| $\rho_{calc}$g/cm$^3$ | 1.560 |
| μ/mm$^{-1}$ | 0.133 |
| F(000) | 312.0 |
| Crystal size/mm$^3$ | 0.35 × 0.17 × 0.06 |
| Radiation | MoK$\alpha$ ($\lambda$ = 0.71073) |
| 2Θ range for data collection/° | 5.272 to 55.996 |
| Index ranges | $-9 \le h \le 9$, $-10 \le k \le 10$, $-16 \le l \le 15$ |
| Reflections collected | 12595 |
| Independent reflections | 3138 [$R_{int}$ = 0.0521, $R_{sigma}$ = 0.0453] |
| Data/restraints/parameters | 3138/0/199 |
| Goodness-of-fit on $F^2$ | 1.033 |
| Final R indexes [I>=2$\sigma$ (I)] | $R_1$ = 0.0415, $wR_2$ = 0.1082 |
| Final R indexes [all data] | $R_1$ = 0.0518, $wR_2$ = 0.1138 |
| Largest diff. peak/hole [e/Å$^3$] | 0.58/−0.25 |

## 3.5 Crystallographic data for 3i

2,2-Difluoro-1,3-bis(4-nitrophenyl)-propane-1,3-dione **3i**

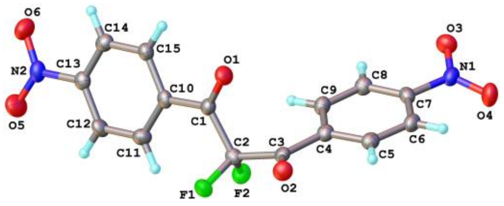


Crystal data and structure refinement for **3i**

| | |
|---|---|
| CCDC | 2288845 |
| Empirical formula | $C_{15}H_8F_2N_2O_6$ |
| Formula weight | 350.23 |
| Temperature/K | 120.0 |
| Crystal system | monoclinic |
| Space group | $P2_1/c$ |
| a/ Å | 13.4814(12) |
| b/ Å | 6.9481(5) |
| c/ Å | 15.0112(11) |
| $\alpha$/° | 90 |
| $\beta$/° | 96.591(7) |
| $\gamma$/° | 90 |
| Volume/ Å$^3$ | 1396.82(19) |
| Z | 4 |
| $\rho_{calc}$g/cm$^3$ | 1.665 |
| μ/mm$^{-1}$ | 0.147 |
| F(000) | 712.0 |
| Crystal size/mm$^3$ | 0.34 × 0.3 × 0.02 |
| Radiation | MoK$\alpha$ ($\lambda$ = 0.71073) |
| 2Θ range for data collection/° | 5.464 to 55 |
| Index ranges | $-17 \le h \le 17$, $-9 \le k \le 8$, $-19 \le l \le 19$ |
| Reflections collected | 17018 |
| Independent reflections | 3204 [$R_{int}$ = 0.0904, $R_{sigma}$ = 0.0730] |
| Data/restraints/parameters | 3204/0/226 |
| Goodness-of-fit on F$^2$ | 1.018 |
| Final R indexes [I>=2$\sigma$ (I)] | $R_1$ = 0.0841, w$R_2$ = 0.2604 |
| Final R indexes [all data] | $R_1$ = 0.1276, w$R_2$ = 0.2511 |
| Largest diff. peak/hole [e/Å$^3$] | 0.87/−0.34 |

### 3.6 Crystallographic data for 4c

Ethyl (4-chloro)benzoylacetate **3c**

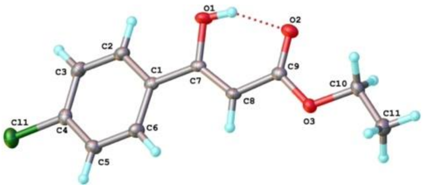


Crystal data and structure refinement for **3c**

| | |
|---|---|
| CCDC | 2288846 |
| Empirical formula | $C_{11}H_{11}ClO_3$ |
| Formula weight | 226.65 |
| Temperature/K | 120.0 |
| Crystal system | monoclinic |
| Space group | $P2_1/c$ |
| a/ Å | 5.5336(3) |
| b/ Å | 26.5949(14) |
| c/ Å | 7.2914(4) |
| $\alpha$/° | 90 |
| $\beta$/° | 102.921(2) |
| $\gamma$/° | 90 |
| Volume/ Å$^3$ | 1045.87(10) |
| Z | 4 |
| $\rho_{calc}$g/cm$^3$ | 1.439 |
| μ/mm$^{-1}$ | 0.348 |
| F(000) | 472.0 |
| Crystal size/mm$^3$ | 0.23 × 0.17 × 0.03 |
| Radiation | MoK$\alpha$ ($\lambda$ = 0.71073) |
| 2Θ range for data collection/° | 5.934 to 58.998 |
| Index ranges | $-7 \le h \le 7$, $-36 \le k \le 36$, $-10 \le l \le 9$ |
| Reflections collected | 21815 |
| Independent reflections | 2903 [$R_{int}$ = 0.0410, $R_{sigma}$ = 0.0280] |
| Data/restraints/parameters | 2903/0/180 |
| Goodness-of-fit on F$^2$ | 1.056 |
| Final R indexes [I>=2$\sigma$ (I)] | $R_1$ = 0.0394, w$R_2$ = 0.0878 |
| Final R indexes [all data] | $R_1$ = 0.0532, w$R_2$ = 0.0927 |
| Largest diff. peak/hole [e/Å$^3$] | 0.38/−0.24 |

## 3.7 Crystallographic data for 4g

Ethyl (3-nitro)benzoylacetate **4g**

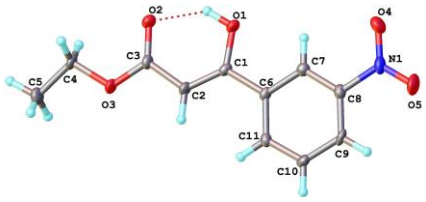


Crystal data and structure refinement for **4g**

| | |
|---|---|
| CCDC | 2288847 |
| Empirical formula | $C_{11}H_{11}NO_5$ |
| Formula weight | 237.21 |
| Temperature/K | 120.0 |
| Crystal system | triclinic |
| Space group | P-1 |
| a/ Å | 8.6967(10) |
| b/ Å | 8.7824(10) |
| c/ Å | 15.0932(17) |
| $\alpha$/° | 76.146(4) |
| $\beta$/° | 76.070(4) |
| $\gamma$/° | 76.820(5) |
| Volume/ Å$^3$ | 1068.4(2) |
| Z | 4 |
| $\rho_{calc}$g/cm$^3$ | 1.475 |
| μ/mm$^{-1}$ | 0.118 |
| F(000) | 496.0 |
| Crystal size/mm$^3$ | 0.34 × 0.17 × 0.08 |
| Radiation | MoK$\alpha$ ($\lambda$ = 0.71073) |
| 2Θ range for data collection/° | 4.858 to 56.998 |
| Index ranges | $-11 \leq h \leq 11$, $-11 \leq k \leq 11$, $-20 \leq l \leq 20$ |
| Reflections collected | 19986 |
| Independent reflections | 5398 [$R_{int}$ = 0.0527, $R_{sigma}$ = 0.0661] |
| Data/restraints/parameters | 5398/0/318 |
| Goodness-of-fit on F$^2$ | 1.030 |
| Final R indexes [I>=2$\sigma$ (I)] | $R_1$ = 0.0630, w$R_2$ = 0.1624 |
| Final R indexes [all data] | $R_1$ = 0.0940, w$R_2$ = 0.1859 |
| Largest diff. peak/hole [e/Å$^3$] | 0.68/−0.41 |

## 3.8 Crystallographic data for 5e

Ethyl 2,2-difluoro-3-(4-(nitrophenyl)-3-oxo-propanoate **5e**

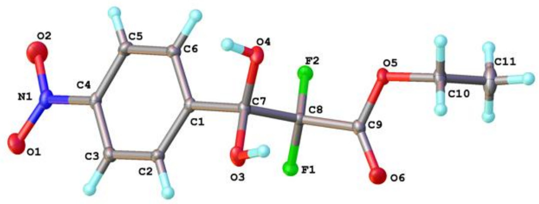


Crystal data and structure refinement for **5e**

| | |
|---|---|
| CCDC | 2288848 |
| Empirical formula | $C_{11}H_{11}F_2NO_6$ |
| Formula weight | 291.21 |
| Temperature/K | 120.0 |
| Crystal system | triclinic |
| Space group | P-1 |
| a/ Å | 7.0838(5) |
| b/ Å | 9.1649(7) |
| c/ Å | 9.4064(7) |
| $\alpha$/° | 92.399(3) |
| $\beta$/° | 97.285(3) |
| $\gamma$/° | 106.626(3) |
| Volume/ Å$^3$ | 578.46(7) |
| Z | 2 |
| $\rho_{calc}$g/cm$^3$ | 1.672 |
| μ/mm$^{-1}$ | 0.156 |
| F(000) | 300.0 |
| Crystal size/mm$^3$ | 0.43 × 0.21 × 0.1 |
| Radiation | MoKα (λ = 0.71073) |
| 2Θ range for data collection/° | 4.38 to 58 |
| Index ranges | -9 ≤ h ≤ 9, -12 ≤ k ≤ 12, -12 ≤ l ≤ 12 |
| Reflections collected | 11966 |
| Independent reflections | 3062 [$R_{int}$ = 0.0380, $R_{sigma}$ = 0.0318] |
| Data/restraints/parameters | 3062/0/225 |
| Goodness-of-fit on F$^2$ | 1.062 |
| Final R indexes [I>=2$\sigma$ (I)] | $R_1$ = 0.0333, w$R_2$ = 0.0896 |
| Final R indexes [all data] | $R_1$ = 0.0407, w$R_2$ = 0.0940 |
| Largest diff. peak/hole [e/Å$^3$] | 0.44/-0.24 |

# 4 References for Supplementary Information


[1] I. Urasaki and Y. Ogata, *Journal of the Chemical Society-Perkin Transactions 1* **1975**, 1285-1287.

[2] S. Nakaiida, S. Kato, O. Niyomura, M. Ishida, F. Ando and J. Koketsu, *Phosphorus Sulfur and Silicon and the Related Elements* **2010**, *185*, 930-946.

[3] E. Hasegawa, K. Ishiyama, T. Horaguchi and T. Shimizu, *Journal of Organic Chemistry* **1991**, *56*, 5236.

[4] H. S. P. Rao and N. Muthanna, *European Journal of Organic Chemistry* **2015**, *2015*, 1525-1532.

[5] a) Z. He, X. T. Qi, S. Q. Li, Y. S. Zhao, G. Gao, Y. Lan, Y. W. Wu, J. B. Lan and J. S. You, *Angewandte Chemie-International Edition* **2015**, *54*, 855-859; b) J. Zawadiak and M. Mrzyczek, *Spectrochimica Acta Part a-Molecular and Biomolecular Spectroscopy* **2012**, *96*, 815-819.

[6] H. Ishihara and Y. Hirabayashi, *Chemistry Letters* **1978**, 1007-1010.

[7] J. C. Hubaud, I. Bombarda, L. Decome, J. C. Wallet and E. M. Gaydou, *Journal of Photochemistry and Photobiology B-Biology* **2008**, *92*, 103-109.

[8] M. Lipp, F. Dallacker and S. Munnes, *Annalen Der Chemie-Justus Liebig* **1958**, *618*, 110-117.

[9] S. Choi, H. Oh, J. Sim, E. Yu, S. Shin and C. M. Park, *Organic Letters* **2020**, *22*, 5528-5534.

[10] S. Stavber, B. Sket, B. Zajc and M. Zupan, *Tetrahedron* **1989**, *45*, 6003-6010.

[11] a) T. J. Nash and G. Pattison, *European Journal of Organic Chemistry* **2015**, *2015*, 3779-3786; b) G. Stavber, M. Zupan, M. Jereb and S. Stavber, *Organic Letters* **2004**, *6*, 4973-4976.

[12] J. Qian, W. Yi, X. Huang, J. P. Jasinski and W. Zhang, *Advanced Synthesis & Catalysis* **2016**, *358*, 2811-2816.

[13] a) M. Gao, Y. K. Zhao, C. Zhong, S. S. Liu, P. K. Liu, Q. Yin and L. Hu, *Organic Letters* **2019**, *21*, 5679-5684; b) C. Tarigopula, G. K. Thota and R. Balamurugan, *European Journal of Organic Chemistry* **2016**, *2016*, 5855-5861.

[14] U. Košak, A. Kovač and S. Gobec, *Synlett* **2012**, 1609-1612.

[15] J. Lee, H. Jung, M. Kim, E. Lee, D. Im, W. Aman and J. M. Hah, *Bioorganic & Medicinal Chemistry* **2018**, *26*, 1628-1637.

[16] in *3-(4-Cyano-phenyl)-3-oxo-propionic acid ethyl ester (CAS 49744-93-6)*, *Vol. 2023* Santa Cruz Biotechnology.

[17] S. Korsager, D. U. Nielsen, R. H. Taaning and T. Skrydstrup, *Angewandte Chemie-International Edition* **2013**, *52*, 9763-9766.

[18] H. O. Burrus and G. Powell, *Journal of the American Chemical Society* **1945**, *67*, 1468-1472.

[19] P. Drouhin and R. J. K. Taylor, *European Journal of Organic Chemistry* **2015**, *2015*, 2333-

2336.
[20] a) H.-Y. Zhao, Z. Feng, Z. Luo and X. Zhang, *Angewandte Chemie-International Edition* **2016**, *55*, 10401-10405; b) G. Stavber and S. Stavber, *Advanced Synthesis & Catalysis* **2010**, *352*, 2838-2846.
[21] O. V. Dolomanov, L. J. Bourhis, R. J. Gildea, J. A. K. Howard and H. Puschmann, *Journal of Applied Crystallography* **2009**, *42*, 339-341.
[22] G. M. Sheldrick, *Acta Crystallographica a-Foundation and Advances* **2008**, *64*, 112-122.
